# Genes Encoding Cher-TPR Fusion Proteins Are Predominantly Found in Gene Clusters Encoding Chemosensory Pathways with Alternative Cellular Functions

**DOI:** 10.1371/journal.pone.0045810

**Published:** 2012-09-20

**Authors:** Francisco Muñoz-Martínez, Cristina García-Fontana, Miriam Rico-Jiménez, Carlos Alfonso, Tino Krell

**Affiliations:** 1 Department of Environmental Protection, Estación Experimental del Zaidín, Consejo Superior de Investigaciones Científicas, Granada, Spain; 2 Centro de Investigaciones Biológicas, Madrid, Spain; University of Liverpool, United Kingdom

## Abstract

Chemosensory pathways correspond to major signal transduction mechanisms and can be classified into the functional families flagellum-mediated taxis, type four pili-mediated taxis or pathways with alternative cellular functions (ACF). CheR methyltransferases are core enzymes in all of these families. CheR proteins fused to tetratricopeptide repeat (TPR) domains have been reported and we present an analysis of this uncharacterized family. We show that CheR-TPRs are widely distributed in GRAM-negative but almost absent from GRAM-positive bacteria. Most strains contain a single CheR-TPR and its abundance does not correlate with the number of chemoreceptors. The TPR domain fused to CheR is comparatively short and frequently composed of 2 repeats. The majority of CheR-TPR genes were found in gene clusters that harbor multidomain response regulators in which the REC domain is fused to different output domains like HK, GGDEF, EAL, HPT, AAA, PAS, GAF, additional REC, HTH, phosphatase or combinations thereof. The response regulator architectures coincide with those reported for the ACF family of pathways. Since the presence of multidomain response regulators is a distinctive feature of this pathway family, we conclude that CheR-TPR proteins form part of ACF type pathways. The diversity of response regulator output domains suggests that the ACF pathways form a superfamily which regroups many different regulatory mechanisms, in which all CheR-TPR proteins appear to participate. In the second part we characterize WspC of *Pseudomonas putida,* a representative example of CheR-TPR. The affinities of WspC-Pp for S-adenosylmethionine and S-adenosylhomocysteine were comparable to those of prototypal CheR, indicating that WspC-Pp activity is in analogy to prototypal CheRs controlled by product feed-back inhibition. The removal of the TPR domain did not impact significantly on the binding constants and consequently not on the product feed-back inhibition. WspC-Pp was found to be monomeric, which rules out a role of the TPR domain in self-association.

## Introduction

Protein-protein interactions are essential for life. In many cases single-domain proteins have evolved with the capacity to recognize other proteins or to oligomerize. An alternative evolutionary strategy consists in fusion proteins in which one domain is dedicated to binding other proteins. An example for such domains is the tetratricopeptide repeat (TPR) containing domain [1]. A TPR is composed of 34 amino acids which form a pair of antiparallel helices connected by a short loop [2]. Typically, a TPR domain possess multiple, repetitive TPRs. The TPR was discovered as late as in 1990 [3] and since then the number of reports on different types of TPR domain containing proteins keeps growing.

The primary function of TPR domains consists in the binding to other proteins which is reviewed in Allan and Ratajczak [2] and D'Andrea and Regan [1]. TPR domains are particularly involved in the formation of functional multiprotein complexes. More recently, it has become apparent that some TPR domains mediate self-assembly into higher order structures [4]–[14]. In addition, evidence was presented for a TPR domain that binds to RNA [15], [16]. TPR domain containing proteins are found in eukaryotes and prokaryotes and are involved in a large number of diverse processes as exemplified by a TPR protein which influences grain size in maize [17], a TPR protein involved in the axonal transport in neurons [18] or a chaperone protein [10]. In many cases the ligand of the TPR domain is unknown.

Bacterial signal transduction is primarily mediated by one- and two-component systems as well as by chemosensory pathways [19]. These pathways are present in most prokaryotic species and were proposed to have evolved from two-component systems [20]. Chemosensory pathways are established by a significant number of gene products and gene co-occurrence analyses have led to the identification of core proteins, namely the chemoreceptors, the sensor kinase CheA, the coupling protein CheW, the methyltransferase CheR and the methylesterase CheB [20]. Auxiliary proteins that were found in fewer genomes include the phosphatases CheC, CheX and CheZ as well as the glutamine deamidase/glutamate methylesterase CheD and CheV.

Chemoreceptor mediated signaling is based on the concerted action of the excitatory pathway and adaptational mechanism(s). The initial step of the excitatory pathway consists in the recognition of signal molecules by chemoreceptors, which produces a molecular stimulus that is transduced across the membrane to the signaling domain that forms a ternary complex with CheA and CheW. This molecular stimulus alters CheA autophosphorylation and in turn transphosphorylation activity towards the response regulator CheY [21]. Adaptational mechanisms are indispensable for chemosensing and correspond to a restoration of the pre-stimulus behavior in the presence of the stimulus. The canonical adaptation mechanism consists in the methylation and demethylation of chemoreceptors which is catalyzed by CheR methyltransferases and CheB methylesterases, respectively [21]. Ligand binding at the chemoreceptor increases CheR mediated methylation of residues at the signaling domain, which in turn modulates the capacity of the receptor to alter CheA autophosphorylation [22]. Auxiliary proteins CheCXZDV participate in some systems in this response [23].

Based on a bioinformatic analysis Wuichet and Zhulin [20] were able to establish 19 different types of chemosensory pathways. Matching these data with experimental evidence resulted in the definition of 3 different functional families of chemosensory pathways namely those that regulate flagellar motility (Fla), those that are involved in type IV pili mediated motility (Tfp) and those with alternative cellular functions (ACF). Interestingly, the Fla family is constituted by 17 of the 19 pathway types, whereas the Tfp and ACP functional families are each formed by a single type of pathway. The ACP pathways contain frequently multidomain response regulators in which the receiver domain is fused to different output domains. In the remaining 18 types of chemosensory pathways multidomain response regulators are almost absent [20].

Chemosensory pathways have been first described in the context of flagellum-mediated taxis, where it was shown that the phosphorylation of CheY induces a conformational change that permits an interaction with the flagellar motor [21]. An example for chemosensory pathways that are not related to flagellum-mediated taxis is *Myxococcus xanthus* [24]. This organism lacks flagellum genes but has 8 chemosensory gene clusters. Some of these clusters are involved in motility whereas others can be associated with alternative cellular processes [24]. *Pseudomonas aeruginosa* has 4 chemosensory clusters of which two are involved in flagellum-mediated taxis [25], one in type IV pili mediated taxis [26] as well as an ACF pathway that was shown to control biofilm formation via modulation of the cyclic di-GMP concentration [27].

The classification of CheR as a core protein is supported by experimental data which showed that mutation of the *cheR* genes abolished or impaired chemotactic behavior in many species [28]–[31]. Enterobacteria have a single, prototypal CheR, which is of less than 290 amino acids length [32]. The methylation process uses S-adenosylmethionine (SAM) as substrate that is converted into S-adenosylhomocysteine (SAH). However, Scott et al. [33] have reported that a CheR-TPR fusion protein, FrzF, methylates the FrzCD receptor of *M. xanthus*. FrzF is composed of a methyltransferase domain that is fused to a TPR domain. The authors show that full-length FrzF methylates a single amino acid, whereas a truncated version lacking the TPR domain methylated the protein at 3 amino acids. This implies that the presence of the TPR domain inhibits methylation activity. The mechanism of action of this CheR-TPR fusion protein is unknown, but Scott et al. [33] hypothesize that FrzF methylation activity is controlled by the TPR fusion, that may form a physical barrier preventing efficient methylation. The binding of a third, unknown protein to the TPR domain may relieve this inhibition.

There appear to be two families of bacterial methyltransferases: the prototypal CheR and the family of CheR-TPR fusions. Prototypal CheRs have been studied in depth which contrasts the scarce information available on the CheR-TPR family. We present here a first analysis of the family of CheR-TPR proteins. In the initial part of this article we have screened completed bacterial genomes sequences to retrieve CheR-TPR sequences, which were then submitted to bioinformatic analyses. In the second part we report the analysis of a representative example of a CheR-TPR protein as well as truncated versions thereof lacking the TPR domain.

## Results

### CheR-TPR are Widely Distributed in GRAM-negative Bacteria

**Figure 1:**
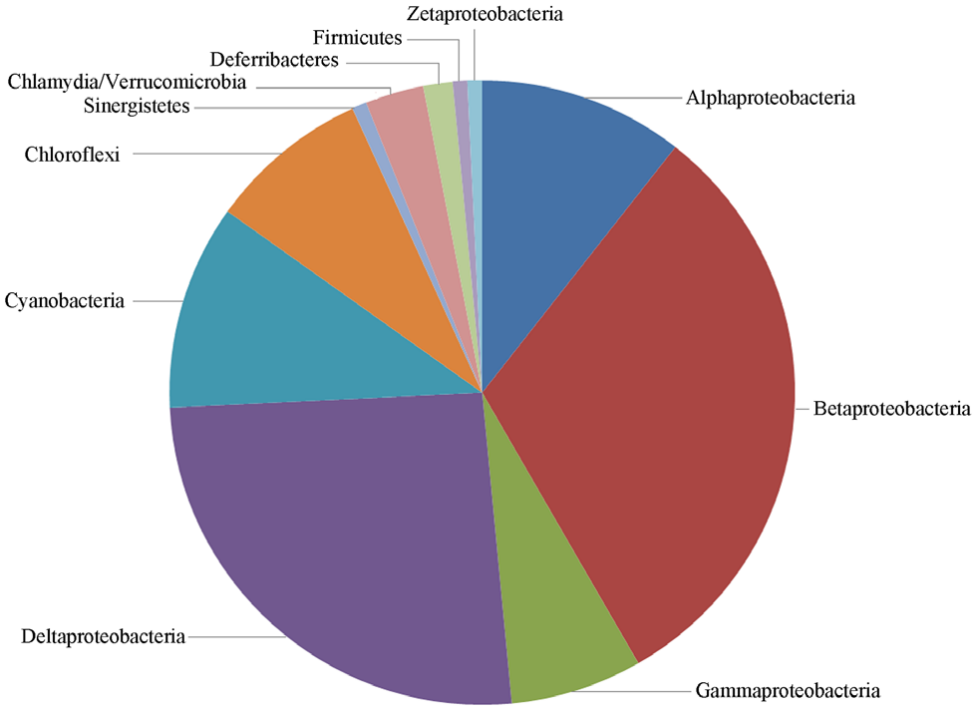
Phylogenetic distribution of species which contain genes encoding CheR-TPR fusion proteins. With the exception of Firmicutes, the remaining taxa represent GRAM-negative bacteria. The species and accession numbers of the individual CheR-TPR proteins are provided in Table S1.

**Figure 2:**
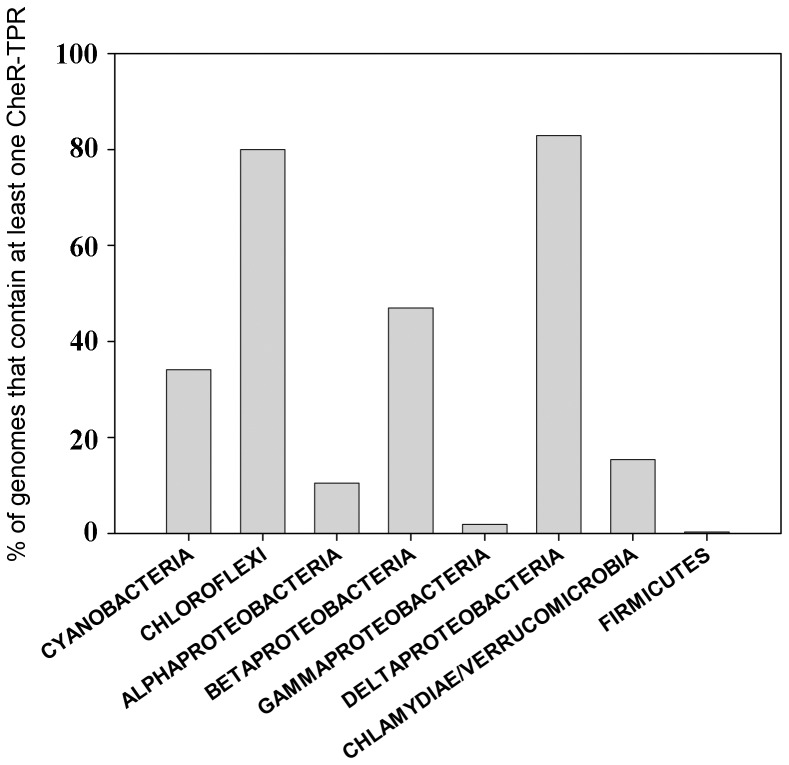
Relative abundance of strains within a taxon that contain at least one *cheR-TPR* gene. Number of genomes analyzed for each taxon were: Cyanobactria: 41; Chloroflexi: 15; Alphaproteobactria: 143; Betaproteobacteria: 91; Gammaproteobacteria: 464; Deltaproteobacteria: 41; Chlamydiae/Verrucomicrobia: 26; Firmicutes: 326.

The analysis of translated open reading frames of completed genome sequences resulted in the detection of 160 CheR-TPR sequences which were then manually curated to exclude false positive hits. The final list contained 132 CheR-TPR proteins that were encoded in 96 genomes. Detailed information on these strains and proteins is compiled in the Table S1. Ninety five of these genomes were of GRAM-negative bacteria whereas a single GRAM-positive strain (*Heliobacterium modesticaldum*) contained a CheR-TPR sequence. As shown in Figure 1, CheR-TPR proteins show a broad phylogenetic distribution and are well presented in alpha-, beta-, gamma- and delta-Proteobacteria as well as in Cyanobacteria and Chloroflexi. The data on the total abundance of CheR-TPR normalized with the number of genomes analyzed per taxon are shown in Figure 2. Around 80% of Chloroflexi and Deltaproteobacteria and around 50% of Betaproteobacteria contain at least one CheR-TPR protein. In contrast, CheR-TPR proteins are very rare in Gammaproteobacteria and Firmicutes. The large majority of this latter phylum is composed of GRAM-positive bacteria, like Bacilli and Clostridia, which confirms that CheR-TPR proteins are almost exclusively found in GRAM-negative species.

### Most Genomes have a Single CheR-TPR Gene

**Figure 3:**
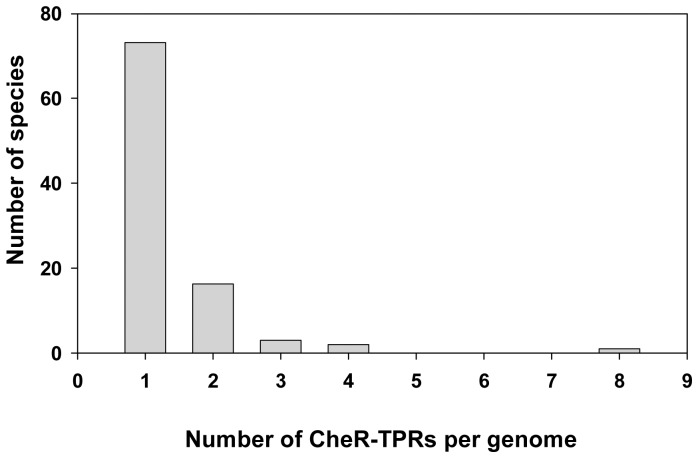
Classification of strains according to the number of *cheR-TPR* genes per genome.

**Figure 4:**
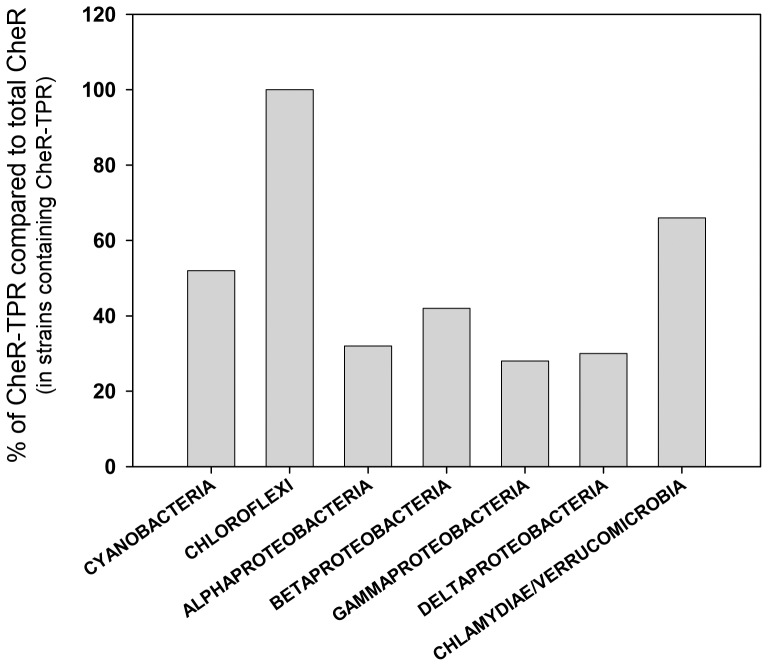
Ratio of *cheR-TPR* genes over total number of *cheR* genes for different taxons.

The 96 CheR-TPR containing genomes encode on average 3.7 CheRs and the large majority of genomes encode a single CheR-TPR (Figure 3). The myxobacterium *Stigmatella aurantiaca* is an outlier, since this species has 19 CheRs of which 8 are CheR-TPR fusions (Figure 3). Myxobacteria are characterized by complex signaling network, are devoid of flagellum mediated taxis but move on surfaces by gliding [34]. The ratio of prototypal CheR over CheR-TPR is around 3/1 for alpha-, beta-, gamma- and delta-Proteobacteria which is consistent with the above information that strains contain frequently a single CheR-TPR (Figure 4). Interestingly, the CheR-TPR containing Chloroflexi do not possess any prototypal CheR. Analysis of their genomes revealed an absence of flagellar genes.

### No Correlation between the Number of Chemoreceptors and CheR-TPRs

**Figure 5:**
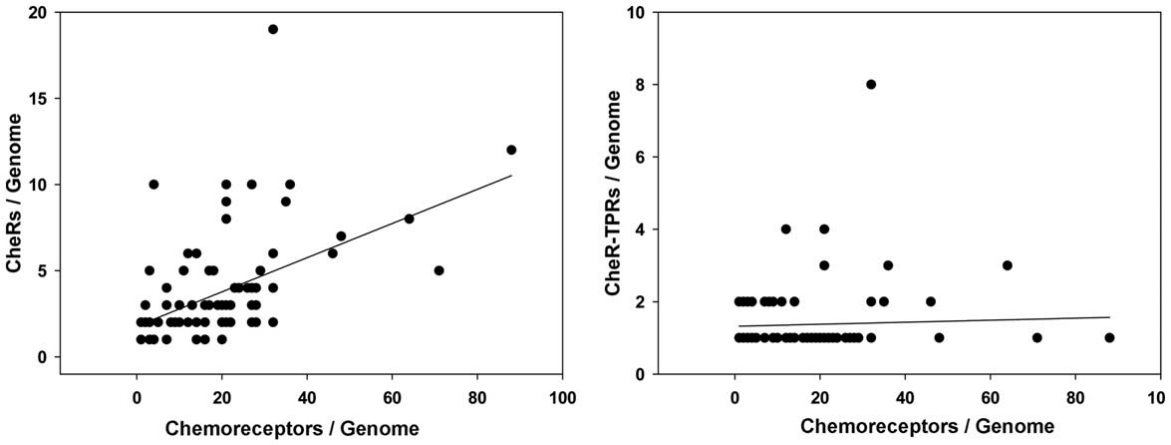
Plot of the number of total *cheR* genes (A) and *cheR-TPR* genes (B) over the number of chemoreceptor genes per strain. The linear regression of these data resulted in y = 0.099 x +1.79 (r^2^ = 0.24) (A) and y = 0.003x +1.321 (r^2^ = 0.0019) (B).

Chemoreceptors are the substrates of CheR methyltransferases. It has been shown that there is a weak correlation between genome size and the number of chemoreceptors [19], [35], [36]. In addition, the number of chemoreceptors depends on the bacterial lifestyle [35], [36]. We wanted to establish whether there is a correlation between the number of chemoreceptors and the number of total CheRs or CheR-TPRs. The plot of the number of total CheR/genome against the number of chemoreceptors/genome is shown in Figure 5A (for the 96 genomes with CheR-TPR). The r^2^ value of the linear regression was 0.24, indicative of a weak correlation. In contrast, the plot of CheR-TPR/genome against the number of chemoreceptors/genome (Figure 5B) showed no correlation (r^2^ of 0.0019). These data are consistent with the observation that most species contain a single CheR-TPR independent of the number of chemoreceptors (Figure 4). In this context a clear example are the 35 strains of Burkolderia and Methylobacteria (Table S1) which contain between 12–48 chemoreceptors but only a single CheR-TPR.

### CheR-TPR Possess a Small TPR Domain

**Figure 6:**
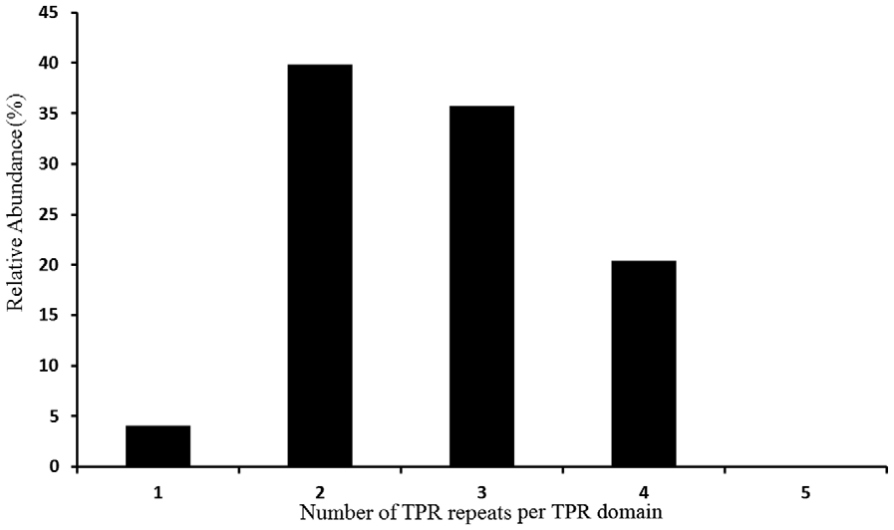
Classification of CheR-TPR fusion proteins according to the number of repeats per TPR domain.

The genome analysis of TPR-containing proteins in all kingdoms of life [1] has revealed that TPR domains with three repeats are most abundant, whereas almost no TPR domains containing one or two repeats were observed. The authors of this study concluded that a minimal number of 3 TPR is necessary to form a functional TPR binding domain. With the exception of 4 CheR-TPR sequences that contain TPR domains at its N-terminus, the TPR domain is present on the C-terminal part of the CheR-TPR fusion. For the latter family, the TPR domains were found to contain 1–4 TPRs (Figure 6). Interestingly, the 2 repeat-containing domain was found to be the most abundant form. Using the assumption of D’Andrea and Regan [1] that consecutive TPRs form binding domains our data are consistent with the notion that two TPRs can form a functional TPR domain. Compared to the totality of bacterial TPR containing proteins [1], it can be concluded that CheR-TPR are characterized by comparatively small TPR domain.

### *Cher-TPR* Genes are Predominantly Found in Gene Clusters which Contain Multidomain CheY Response Regulators

**Figure 7:**
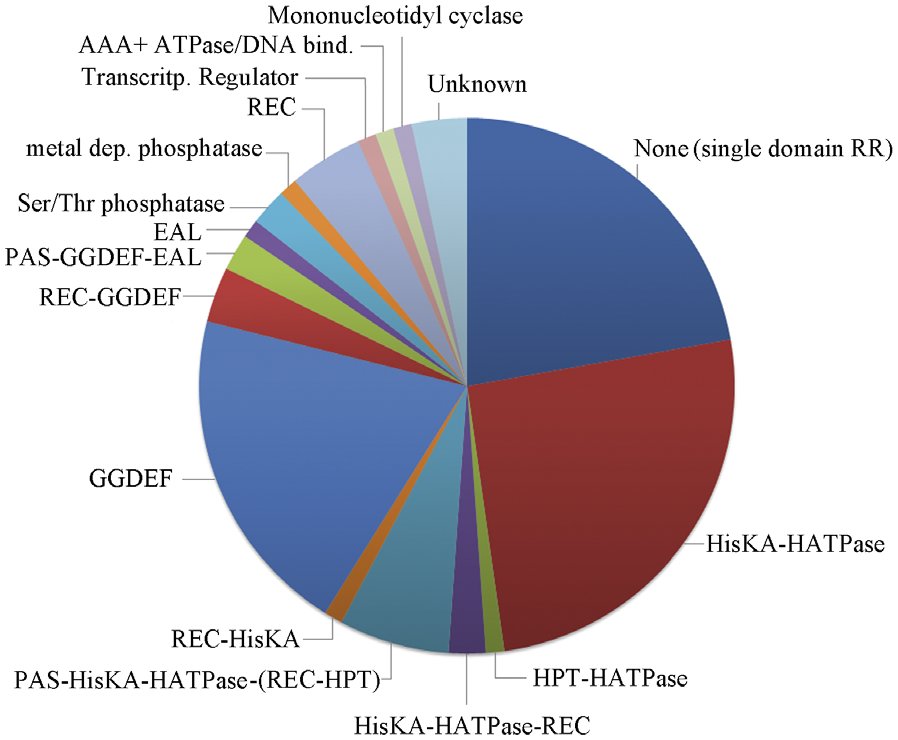
Architecture of response regulators present in *cheR-TPR* containing gene clusters. The segment shown in dark blue corresponds to single-domain response regulators that are composed of a single receiver domain (REC). The remaining response regulators are multidomain proteins and listed are the domain(s) which are fused to the REC domain. **HISKA**: histidine kinase A (dimerization/phosphoacceptor) domain, cl00080; **HATPase**: histidine kinase-like ATPases, cd00075: **HPT**: histidine phosphotransfer domain, cd 00088; **REC**: response regulator receiver domain, cd 00156; **PAS**: superfamily of per-arn-sim domains, cl02459; **GGDEF**: diguanylate-cyclase, cd01949; **EAL**: diguanylate phosphodiesterase, cd 01948; **Ser/Thr phosphatase**: family 2C, cl00120; **metal-dependent phosphatase**: cd 00077; **transcriptional regulator**: cd00383; **AAA+ ATPase**: cd 00009; **mononucleotidyl cyclase**: cd 07302, contain adenylate cyclases and guanylate cyclases. Detailed information of this analysis is found in Table S2.

Chemosensory pathways can be classified into three functional families. A frequent feature of the alternative cellular function (ACF) family is the presence of multidomain response regulators, whereas the response regulators that participate in flagellum-mediated taxis (Fla) pathways or type four pili-mediated taxis (Tfp) are with high frequency single domain response regulators [20]. We have therefore analyzed the genetic environment of the *cheR-TPR* genes. Most of the *cheR-TPR* genes are present in chemosensory gene clusters and most of these clusters contain a response regulator (Table S2). Only 20 response regulators were single-domain proteins, comprised of only a REC domain (Figure 7). To evaluate whether these 20 single-domain response regulators may be involved in flagellum- or type IV pili-mediated taxis, the corresponding genomes were analyzed for the existence of flagella (presence of FliM, FliC, FlgH and FliE homologues) or type IV pili genes (presence of PilQ and PilT homologues). With the exception of the Geobacter strains and the only GRAM-positive strain, *Heliobacterium modesticaldum,* all single-domain response regulators are present in species that lack flagellum genes. In analogy only half of the 20 strains contained genes of type IV pili.

However, the large majority of response regulators present in *cheR-TPR* containing gene clusters are multidomain proteins (Figure 7, Table S2). Interestingly, the REC domain of the response regulators is fused to a variety of different output domains such as histidine kinase, GGDEF, EAL, Histidine containing phosphotransfer domain (HPT), AAA, PAS, GAF, additional REC, HTH DNA-binding, protein phosphatase, mononucleotidyl cyclase or various combinations thereof. Almost all of the additional domains of these response regulators can be associated with signal transduction processes. Around 40% of these response regulator sequences contained a histidine kinase module, composed of HisKA and HATPase domains (Figure 7). This histidine kinase module is the central part of sensor kinases [37]. In sensor kinases, typically signal recognition by the sensor domain modulates the activity of the autokinase module. For response regulators fused to an autokinase module, it appears likely that REC domain phosphorylation may modulate autokinase activity.

The other large group of multidomain response regulators in *cheR-TPR* containing clusters has domains that are involved in the synthesis and hydrolysis of the second messenger c-di-GMP (Figure 7). Some 20 regulators contain a GGDEF domain (synthesis of c-di-GMP), another regulator contains an EAL domain (hydrolysis of c-di-GMP), and in another 2 cases the REC domain is fused to both domains. Further multidomain response regulators contain different types of protein phosphatases, which also play important roles in bacterial signal transduction [38]. Other additional domains share similarities with transcriptional regulators or DNA binding domains suggesting a role in transcriptional regulation. In some cases REC domains fused to histidine kinase, GGDEF or EAL domains contain in addition PAS or GAF sensor domains. It can be hypothesized that these sensor domains recognize signal molecules, which in turn may modulate the catalytic activities of the histidine kinase, GGDEF or EAL domains. Interestingly, the domain architecture of response regulators is almost identical to that of response regulators of the ACF family of chemosensory pathways (Table S1 of [20]). The same authors also show that some ACF pathways possess single domain response regulators. In addition, the phylogenetic distribution of CheR-TPR genes is very similar to that of the ACF family [20].

### CheR-TPRs are a Heterogeneous Family but Catalytic Residues of the CheR Domains are Conserved

CheR-TPR sequences were found to differ in size, which ranged from 320 to 690 amino acids (Figure S1). These differences are due to the different number of TPRs per domain (Figure 6) as well as to the differences in the length of the linker region (Figure S2), which was found to range between 30–340 amino acids. An important question in the analysis of this protein family consists in obtaining evidence for the functionality of the CheR domain. The analysis of the CheR structure of *S. typhimurium* in complex with SAH has resulted in the identification of two key catalytic residues, R98 and Y235 [32] (Figure S3). This is supported by site-directed mutagenesis studies which showed that bacteria harboring the R98A mutation failed to swarm [39]. The sequences of CheR with C-terminal TPR (Figure S4A) align well with the *E. coli* and *S. typhimurium* sequences and both catalytic residues are fully conserved. In contrast, the 4 CheR-TPR sequences with N-terminal TPR align poorly with the enterobacterial proteins, and catalytic residues are not conserved (Figure S4B). This may indicate that the CheR-TPR sub-family with C-terminal TPR corresponds to functional methyltransferases whereas the small subfamily with N-terminal TPR might correspond to inactive methyltransferases.

### Analysis of Total TPR Genes in CheR-TPR Containing Genomes

The target molecules of the TPR moieties of the CheR-TPR fusions are unknown. Since some TPR domains were found to interact with other TPR domains, we hypothesized that other TPR containing proteins may be the binding partners of CheR-TPRs. We retrieved and analyzed all TPR domain containing genes in the CheR-TPR containing genomes (Table S3). The abundance of TPR containing genes differed significantly and ranged from 5 to 206 genes, which translates into a relative abundance of 0.13–2.46% of the total number of genes. On average, these genomes contained 31 TPR genes which correspond to an average abundance of 0.58% of the total gene number. The individual taxons were characterized by a different abundance of TRP genes. TPR proteins were most abundant in Cyanobacteria and Deltaproteobacteria where they represent 1.17 and 1.27% of the total genes, respectively (Figure S5).

### Identification and Initial Characterization of the Chemoreceptor-TPR Family

The inspection of cellular TPR proteins provided some interesting clues as to potential targets for CheR-TPRs. CheRs methylate chemoreceptors and initial analyses were aimed at elucidating whether there may be chemoreceptor-TPR fusion proteins. Interestingly, sequence analysis with InterPro revealed that 9 out of the 10 cyanobacterial genomes analyzed contained chemoreceptor-TPR fusion proteins (Table S2). Furthermore, these strains were found to lack flagellum genes. An initial analysis of these sequences is shown in Analysis S1. These chemoreceptors contain an 68–82 amino acid fragment that is recognized by InterPro signature IPR011990 (TPR-like helical). In all cases the TPR was located at the N-terminal part of the receptor. To get inside into the protein topology, the transmembrane regions of these proteins were predicted. For all sequences two transmembrane regions separated by a stretch of 13–65 amino acids were identified in the central part of the receptor (Analysis S1). The C-terminal part of the receptor was identified as methylaccepting chemotaxis signaling domain (IPR004089). This indicates that both, the N- and C-terminal half of these receptors are located in the cytosol, whereas the loop connecting both transmembrane regions is located in the periplasm. The TPR domain and the signaling domain are thus present in the same cellular compartment. Apart from the TPR region, no other domain is annotated in the N-terminal half of the protein. To exclude the possibility that the annotation of the TPR domains are a false positive hit, the TPR containing fragments were submitted to three dimensional homology modeling algorithms. In all cases homology models were obtained (Analysis S1) that reveal TPR domain structures. A sequence alignment of chemoreceptor-TPR fusion showed an identity of 21.5% with strong conservation of the TPR and the signaling domain (Analysis S1). Analysis of all currently available chemoreceptor sequences revealed that the family of chemoreceptor-TPR fusion proteins is exclusively found in Cyanobacteria.

### Identification of TPR Proteins Involved in Non-flagellum Mediated Taxis

FrzF of *M. xanthus* was the first CheR-TPR protein described [33]. This species employs two different non-flagellar gliding motility mechanisms, namely the S- and A-motility [34]. A recent mutagenesis study has identified 4 TPR proteins (AgmU, AgnA, AglT, AgmK) as part of the motility machinery [40]. Further studies and genome analysis reveals the presence of other TPR proteins in *M. xanthus* motility such as Tgl [41], protein T (Q1D701) or Q1D897. Homology modeling of these proteins has confirmed their TPR structure (Analysis S2). The mechanism of action of these proteins is unknown but AglT and AgmK were found to be part of a protein complex that was found to bind to the FrzCD chemoreceptor, the substrate of the FrzF methyltransferase [42]. It is therefore possible that the TPR domains of FrzF and AgmK interact. However, our analyses show that AglT and AgmK homologues are exclusively found in the two myxobacterial strains *M. xanthus* and *Stigmatella aurantiaca* (Table S3).

We show that CheR-TPR are almost exclusively found in GRAM negative species. Since the twitching motility is also almost restricted to this group of bacteria [43] we investigated whether there may be a link between CheR-TPR and the twitching motility. The TPR protein PilF was found to play a role in twitching motility. The protein is of unknown function, is anchored to the inner membrane via an N-terminal transmembrane region and is present in the cytosol [44]. Its three dimensional structure is composed of a TPR domain containing 6 repeats. The binding partner for PilF has not been identified. We have searched for PilF homologues in the CheR-TPR containing strains (Table S3). However, only around 17% of strains were found to possess a PilF homologue (Table S1).

### Removal of Linker/TPR Domain Modifies Binding Energetics of SAM and SAH to WspC-Pp but Leaves Affinity Almost Unchanged

Following the bioinformatic studies we proceeded with the functional characterization of a representative member of the CheR-TPR family. WspC of *P. aeruginosa* is a CheR-TPR and forms part of the wsp pathway [27]. The *wsp* gene cluster is a highly conserved feature of *Pseudomonas* genomes [45]–[47]. The ORF PP1490 of *P. putida* KT2440 shares 55% sequence identity with WspC and was renamed named WspC-Pp. The DNA fragment encoding the full-length protein as well as two shortened versions, WspC-Ppmiddle and WspC-Ppshort were cloned into a protein expression vector. WspC-Ppmiddle comprises amino acids 1–355 of WspC-Pp and lacks the TPR domain, whereas WspC-Ppshort (amino acids 1–240 of WspC-Pp) is devoid of linker and TPR domain. WspC-Ppshort can thus be considered as prototypal CheR. The three proteins were expressed in *E. coli* and purified from the soluble fraction of the cell lysate.

**Figure 8:**
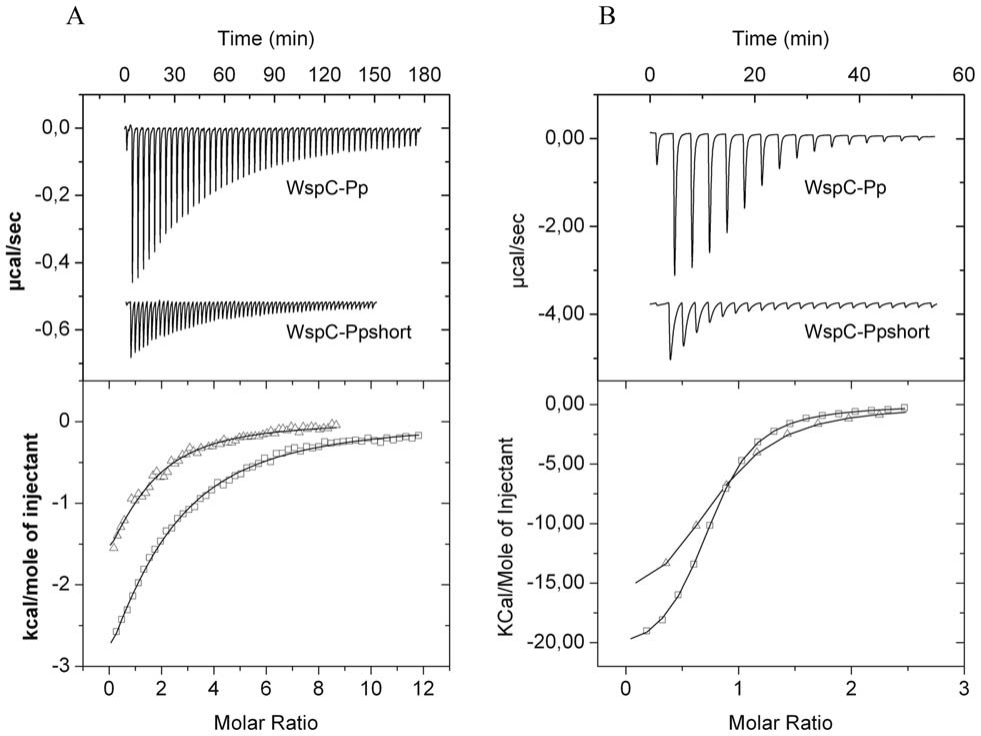
Microcalorimetric titrations of WspC-Pp and WspC-Ppshort with of SAM (A) and SAH (B). Upper panels: (A) Raw data for the titration of WspC-Pp (16 µM) and WspC-Ppshort (25 µM) with 4.8 µl aliquots of 1 mM SAM. (B) Titration of WspC-Pp (24 µM) and WspC-Ppshort (12 µM) with 1 mM SAH. Lower panels: Integrated, dilution-corrected and concentration-normalized raw data, which were then fitted using the “One binding site model” of the ORIGIN version from MicroCal (Northampton, MA, USA)., □ WspC-Pp and Δ WspC-Pp–short.

To study SAM and SAH binding, the three proteins were submitted to isothermal titration calorimetry studies [48] and the derived thermodynamic parameters are given in Table 1. The titration of WspC-Pp with SAM and SAH resulted in exothermic heat changes indicative of favorable enthalpy changes (Figure 8). WspC-Pp bound SAM with an affinity of 43±2 µM and removal of the TPR domain or the linker plus TPR domain produced only a slight increase in affinity (Table 1). WspC-Pp binds the product SAH (1.7±0.1 µM) much tighter than SAM, which is a feature that has been reported previously for prototypal methyltransferases [49], [50]. In analogy to the experiments with SAM, the shortened versions of WspC-Pp bound SAH which an almost identical affinity as compared to the full-length protein.

**Table 1:** Thermodynamic parameters for the binding of S-adenosylmethionine (SAM) and S-adenosylhomocysteine (SAH) to WspC-Pp and its shortened versions WspC-Ppmiddle (WspC-Pp lacking TPR domain) and WspC-Ppshort (WspC-Pp lacking TPR domain and linker).

Ligand 1	Ligand 2	*K*_D_ (µM)	*K*_A_ (M^−1^)	Δ*H* kcal/mol	TΔS kcal/mol
WspC-Pp	SAM	43±2	(2.3±0.1) 10^4^	−7.43±0.6	−1.49±0.6
WspC-Ppmiddle	SAM	35±2	(2.9±0.1) 10^4^	−3.04±0.4	3.04±0.4
WspC-Ppshort	SAM	41±4	(2.5±0.2) 10^4^	−3.53±0.4	2.46±0.5
WspC-Pp	SAH	1.7±0.1	(5.9±0.1) 10^5^	−21.7±0.1	−13.8±0.1
WspC-Ppmiddle	SAH	1.6±0.1	(6.3±0.2) 10^5^	−14.8±0.2	−6.88±0.2
WspC-Ppshort	SAH	2.0±0.1	(4.9±0.1) 10^5^	−18.6±0.2	−10.8±0.2

Although the linker and TPR domains did not alter significantly the equilibrium constants of binding it became apparent that removal of TPR/linker or TPR domains altered significantly the thermodynamics of binding, namely the ratio of enthalpy to entropy changes. This was particularly pronounced for SAM where an unfavorable entropy change was measured for the full-length protein, whereas ligand binding to the shortened versions was characterized by favorable entropy changes which were in their magnitude comparable to the enthalpy changes. The same tendency is observed for SAH, although to a lesser extent. It can be concluded that the linker/TPR domains have a major impact on the thermodynamics of substrate/product binding, which, however, is not reflected in the affinity of binding.

### WspC-Pp is Monomeric in the Absence and Presence of SAM and SAH

**Figure 9:**
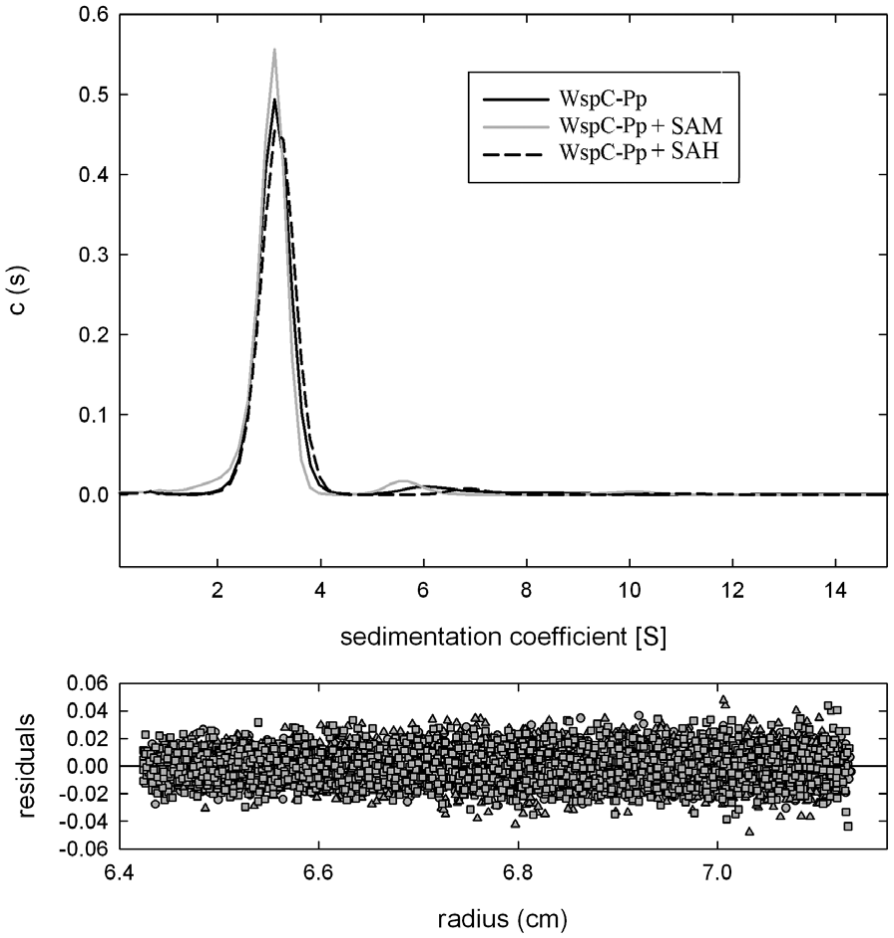
Analyses of WspC-Pp by sedimentation velocity ultracentrifugation. Protein concentration was of 1 mg/ml. Analyses were conducted in the absence and presence of either SAM (added to a final concentration of 500 µM) and SAH (added to a final concentration of 50 µM).

There is a significant number of cases where the TPR domain mediates protein self-assembly [4]–[14]. Simms et al. [51] have shown that the *S. typhimurium* CheR is monomeric is solution. To determine the oligomeric state of WspC-Pp, the protein was analyzed by analytical ultracentrifugation techniques. In sedimentation velocity studies a major peak centered at 2.7 S and a very minor peak at 5.0 S were observed (Figure 9). The addition of SAM and SAH at saturating concentrations did not alter significantly the sedimentation velocity of the protein. Protein concentrations for these analyses were varied from 0.25 to 2 mg/ml and essentially the same results were obtained at each concentration. The Svedberg coefficient of 2.7 is consistent with a monomeric form of the protein. To further characterize the oligomeric species observed in sedimentation velocity studies, WspC-Pp was submitted to equilibrium ultracentrifugation studies. Data analyses revealed a mass of 50 000±1200 for WspC-Pp which is close to the sequence derived mass of 49 052 Da.

## Discussion

Chemoreceptor based signaling is one of the major signal transduction mechanisms in bacteria, as evidenced by the fact that bacteria contain on average 14 chemoreceptor genes [35]. Chemoreceptor methylation by CheR methyltransferases was found to be essential for efficient chemosensing (28–31). The observation that an unusual CheR, the TPR containing FrzF, methylates the FrzCD chemoreceptor [33], [52] has motivated the present study. Here we show that CheR-TPR sequences show a wide phylogenetic distribution in GRAM-negative bacteria but are almost absent from GRAM positive bacteria. Compared to TPR domains of other proteins, CheR-TPR fusions are characterized by a relatively small TPR domain in which the most frequent form is the 2 repeat containing domain. Assuming that repeated TPR sequences form functional binding domains, D’Andrea & Regan [1] have proposed that at least 3 TPRs are necessary to form a TPR binding domain. Here we show that domains with only 2 TPR are most frequently found in CheR-TPR. Using the same assumption we conclude from our data that a domain harboring 2 TPRs is sufficient to form a TPR binding domain.

Data strongly suggest that CheR-TPR proteins are functional methyltransferases. This is evidenced by the conservation of catalytic amino acids (Figures S3 and S4) and by the fact that SAM and SAH bind to WspC-Pp with affinities similar to those of prototypal enzymes [49], [50]. This notion is also supported by the methylation of the FrzCD chemoreceptor by FrzF [33]. In addition, Bantinaki et al. [53] have developed a model for the concerted action of the components of the wsp pathway in which WspC functions as methyltransferase. The effects on the phenotype observed following mutation of the *wspC* gene or its overexpression were consistent with this model [53].

Chemosensory pathways have been discovered due to their role in flagellum-mediated taxis, but subsequent studies have revealed that these pathways also regulate type IV pili mediated motility [26], or alternative cellular functions such as biofilm formation [27], development [24], cell-cell interaction [54] and flagella biosynthesis [55]. Chemosensory pathways consist of core and auxiliary proteins [20] and can be classified into three functional families, namely Fla (flagellum mediated taxis), Tfp (type four pili mediated taxis) and ACF (alternative cellular functions) [20]. The characteristic feature of the ACF family is the presence of multidomain response regulators. Here we show that the large majority of CheR-TPR genes are present in operons that contain multidomain response regulators (Table S2). Interestingly the architecture of these response regulators (Table S2) almost coincides with that of response regulators identified for the ACF family (Table S1 of [20]). Both studies report response regulators in which the REC domains is fused to a Histidine kinase, GGDEF, EAL, HPT, AAA, PAS, GAF, additional REC, HTH DNA binding, protein phosphatase or combinations thereof. It therefore appears that the CheR-TPR family can be associated with the ACF family for chemosensory systems. So far only a limited number of ACF chemosensory pathways have been identified [24], [26], [27], [54], [55], but in the light of the diversity of the response regulators, the ACF family should be considered as a superfamily which regroups a large number of different regulatory mechanisms. The fact that the response regulator architectures in ACF pathways coincides with those present in CheR-TPR (Table S2) indicates that CheR-TPRs are a central element of ACF pathways. This conclusion is supported by the similarities in the phylogenetic distribution of CheR-TPR and ACF family pathways [20]. ACF pathways were detected in Archae, Chloroflexi, Cyanobacteria and Alpha-, Beta-, Gamma- and Deltaproteobacteria, whereas CheR-TPR (Figure 1,2) are found in Chloroflexi, Cyanobacteria and Alpha-, Beta-, Gamma- and Deltaproteobacteria. Interestingly, all chemosensory pathways of Chloroflexi have been classified as ACF [20]. This is in agreement with our observation that 80% of Chloroflexi have at least 1 *cheR-TPR* gene and that CheR-TPR containing Chloroflexi do not possess any prototypal CheR. Our conclusion that CheR-TPRs are primarily involved in ACF pathways is also confirmed by WspR, the response regulator of the wsp pathway, which is a REC-GGDEF fusion [56].

The search for potential targets of the CheR-TPR was based on the possibility that TPR domains may interact with each other. The totality of TPR proteins encoded in the CheR-TPR containing genomes were thus inspected (Table S3). This search resulted in the detection of the chemoreceptor-TPR protein family (Analysis S1). Since chemoreceptors are the substrates of CheR methyltransferases, it appears plausible that chemoreceptor-TPR proteins are the substrate of CheR-TPR. For all family members the sequence-based annotation as TPR protein was confirmed by 3D homology modeling (Analysis S1). The topology predictions show that the TRP domain and the methylaccepting signaling domain are present in the cytosol. It can be hypothesized that a potential interaction between both TPR domains leads to a recruitment of CheR-TPR to a chemoreceptor. In this context parallels exist to chemoreceptors that possess C-terminal pentapeptides, which bind CheR proteins tightly. CheR binding to these pentapeptides is essential for efficient receptor methylation [57]. Since genome analyses have shown that many bacterial species contain receptors with and without pentapeptide as well as multiple CheR paralogues [58], CheR binding to a pentapeptide containing receptors would result in the targeting of a given receptor by a given CheR. A similar mode of interaction may be plausible for the TPR-chemoreceptor fusions.

The analysis of all cellular TPR proteins revealed that at least 7 different proteins are predicted or confirmed to be involved in the gliding motility of *M. xanthus* (Analysis S2). Two of them, AglT and AgmK, form part of a protein complex that binds to the FrzCD receptor [42]; the substrate of the FrzF CheR-TPR methyltransferase. It may be plausible that the TPR domains of these proteins interact. However, AglT and AgmK were only found in the CheR-TPR containing myxobacterial strains (Table S3) and this mode of interaction would only correspond to a class-specific and not to a general mechanism of action. We would like to note that the target of the majority of TPR proteins is unknown [1] and additional experimentation is necessary to identify the CheR-TPT target proteins.

The bioinformatic study was complemented with a functional analysis of WspC-Pp. We were able to show that WspC-Pp recognizes SAM and SAH with affinities of 43 and 1.7 µM, respectively (Table 1). These values are in the same range as the affinities of prototypal CheR, which were 10.9 and 0.23 µM, respectively [49], [50]. A characteristic feature of prototypal CheR is the control of their catalytic activity by product-feedback inhibition, since the product SAH binds tighter than SAM. We are able to conclude that a similar feedback inhibition also occurs in WspC-Pp. Importantly, the removal of the TPR domain modulated binding energetics but did not influence binding affinity (Table 1). It can therefore be ruled out that the TPR domain is involved in a regulation of substrate/binding affinity and, consequently, does not interfere with product-feedback inhibition.

It has been shown that prototypal CheR are monomeric proteins (51). TPR domains are frequently involved in mediating protein self-association [4]–[14] and in some cases the self-association of TPR represents an additional level of regulation permitting the fine-tuning of biological processes [8]–[10]. We therefore assessed the oligomeric state of WspC_Pp in the absence and presence of SAM or SAH (Figure 9). The analytical ultracentrifugation studies presented here demonstrate that WspC-Pp is monomeric in solution. This demonstrates that WspC-Pp is, like the prototypal CheR, a monomeric enzyme which rules out that the TPR mediates protein self-association.

## Materials and Methods

### Sequence Retrieval and Analysis

To retrieve all bacterial CheR genes the Interpro database was searched for sequences which are identified by IPR000780 (chemoreceptor methyltransferase, CheR-type). In total 1499 genomes were used for this analysis. This search resulted in the identification of 2898 sequences. To identify those CheR sequences that contain an additional TPR domain, the retrieved CheR sequences were then screened for the presence of TPR domains. This search was done using the “batch search” tool (http://pfam.sanger.ac.uk/search#tabview=tab1) of Pfam. The criterion for this search was the presence of members of the TPR clan (CL0020) and the E-value threshold for this search was set at 1.0 e^−4^. From the multiple families which form the TPR clan CL002, CheR proteins were found to be fused to TPR domains that belong exclusively to 3 families, namely TPR1 (PF00515), TPR2 (PF07719) and TPR4 (PF07721). The resulting list was then manually curated to confirm the presence of TPR domains. The criteria for the confirmation of a CheR-TPR was the identification of TPR by Interpro (presence of TPR domains IPR011990 or IPR013026) and by the “Search for conserved domains” tool of NCBI (http://www.ncbi.nlm.nih.gov/Structure/cdd/wrpsb.cgi) using the presence of cd00189 domains as criterion. The curated list contained 132 sequences from 96 different genomes. In order to establish whether these sequences belong to a *che*-like gene cluster, their respective genomic contexts were investigated using the “Gene” database of NCBI (http://www.ncbi.nlm.nih.gov/gene). This allowed the identification of the *cheY* type response regulator genes in *cheR-TPR* containing gene clusters. The domain organization of response regulators was extracted from the conserved domains database of NCBI. Data of the retrieved CheR-TPR sequences, information of the host bacterium as well as links to the relevant databases from which information was extracted are provided in Tables S1 and S2.

### Identification and Characterization of Chemoreceptor-TPR Fusion Proteins

Initially the CheR-TPR containing genomes were analyzed for sequences that match InterPro signatures IPR011990 (TPR-like helical) and IPR004089 (methylaccepting chemotaxis protein [MCP] signaling domain). Subsequently, all currently available chemoreceptor sequences were retrieved by a search for hits in all currently available genome sequences using IPR004089. This search resulted in the detection of 29 425 sequences. To verify whether these sequences contain a TPR domain, a series of batch searches were conducted using the “batch search” tool (http://pfam.sanger.ac.uk/search#tabview=tab1) of Pfam. The criterion for this search was the presence of members of the TPR clan (CL0020) and the E-value threshold for this search was set at 1.0 e^−4^. The transmembrane regions of chemoreceptor-TPR fusion proteins were determined using the TMHMM Server v. 2.0 (http://www.cbs.dtu.dk/services/TMHMM-2.0/). The sequence fragments of chemoreceptor predicted to form a TPR domain, flanked by 5 amino acids at each side, were submitted to the three-dimensional homology modeling server CPHmodels 3.2 (http://www.cbs.dtu.dk/services/CPHmodels/). Multiple sequence alignments were carried out using the CLUSTALW algorithm of the NPSA server (http://npsa-pbil.ibcp.fr/cgi-bin/npsa_automat.pl?page=/NPSA/npsa_clustalw.html). The GONNET matrix was used and the gap opening and gap extension penalties were set at 10 and 0.1, respectively.

#### Identification and analysis of TPR proteins in CheR-TPR containing strains

Each of the CheR-TPR containing strains (Table S1) was searched for sequences that match IPR011990 (TPR-like helical). The number of hits and the links to the corresponding internet pages have been introduced into Table S3. Retrieved sequences were inspected manually for TPR protein involved in gliding or twitching, which led to the identification of PilF, AgmU, AgnA, AglT, AgmK, protein T, Tgl and Q1D897. Three dimensional models of these proteins were generated using the CPHmodels 3.2 server. To precisely determine the presence of proteins homologous of PilF, AglT and AgmK, the CheR-TPR containing genomes were submitted to a BLAST search using the sequence of *P. aeruginosa* PilF (Q51526), *M. xanthus* AglT (Q1D2U6) and *M. xanthus* AgmK (Q1D2V1). The criteria for considering a protein as homologue was a sequence identity of more than 30% as well as a size corresponding to ±30% of the search protein. Links of proteins retrieved are provided in Table S3.

### Strains and Plasmids

The strains and plasmids used in this study are provided in Table 2.

**Table 2:** Strains and plasmids used in this study.

Strains	Features	Reference
*P. putida* KT2440	mt-2 pWW0 cured, TolS^−^	46
*E. coli* BL21 (DE3)	F^−^, *ompI, hsdS_B_* (r^−^_B_ m^−^_B_)	62
**Plasmids**	**Features**	**Reference**
pET28b(+)	Km^R^, protein expression vector	Novagen
pET28b-WspC-Pp	Km^R^, pET28b(+) derivative	This work
pET28b-WspC-Ppshort	Km^R^, pET28b(+) derivative	This work
pET28b-WspC-Ppmiddle	Km^R^, pET28b(+) derivative	This work

#### Cloning, expression and purification of WspC-Pp, WspC-Ppshort and WspC-Ppmiddle

DNA sequences encoding full-length WspC-Pp of *Pseudomonas putida* KT2440 (pp1490, Q88MS8), its shortened versions, WspC-Pp-short (amino acids 1–240 of WspC-Pp) and WspC-Pp-middle (amino acids 1–355 of WspC-Pp) were amplified by PCR using the oligonucleotides indicated in Table 3 and genomic DNA of *P. putida* KT2440 as template. The resulting products were digested with *NdeI* and *BamHI* and cloned into pET28b(+) (Novagen) linearized with the same enzymes. The resulting plasmids pET28b-WspC-Pp, pET28b-WspC-Ppshort and pET28b-WspC-Ppmiddle were verified by sequencing the insert and flanking regions.

**Table 3:** Oligonucleotides used in this study.

Name	sequence	Construction of
WspC-Pp-f	5′-AGGGATGCCCATATGAACGAACAGCGTTTC-3′	pET28b-WspC-Pp
WspC-Pp-r	5′-TCGCGAATTGGATCCTCATCGTTCAGACTC-3′	pET28b-WspC-Pp
WspC-Ppshort-f	5′-AGGGATGCCCATATGAACGAACAGCGTTTC-3′	pET28b-WspC-Ppshort
WspC-Ppshort-r	5′-TTGGCAGTGGATCCTCATGCCGCCGCCAA-3′	pET28b-WspC-Ppshort
WspC-Ppmiddle-f	5′-AGGGATGCCCATATGAACGAACAGCGTTTC-3′	pET28b-WspC-Ppmiddle
WspC-Ppmiddle-r	5′-AGTAGGATCCTCATGCCGACGGCGGGTACT-3′	pET28b-WspC-Ppmiddle

For protein expression *E. coli* BL21 (DE3) was transformed with each of the three plasmids constructed above. The resulting strains were grown in 2l Erlenmeyer flask containing 500 ml of LB medium supplemented with 50 µg/ml of kanamycin at 30°C. At an OD_660_ of 0.6 protein production was induced by the addition of 0.1 mM IPTG and growth was continued at 16 degrees C overnight prior to cell harvest by centrifugation at 10 000×g for 30 min. Cell pellets were resuspended in buffer A [20 mM Tris/HCl, 0.1 mM EDTA, 500 mM NaCl, 10 mM Imidazole, 5 mM β-mercaptoetanol, and 5% (vol/vol) glycerol, pH 8.0] and broken using French press at 1000 psi. After centrifugation at 20 000×g for 1 hour, the supernatant was loaded into 5 ml of HisTrap HP column (Amersham Bioscience) equilibrated with buffer A and eluted with an imidazole gradient of 45–500 mM in buffer A.

#### Isothermal titration calorimetry

Measurements were done on a VP-microcalorimeter (MicroCal, Northampton, MA, USA) at 25°C. Protein was dialyzed into 20 mM Tris/HCl, 150 mM NaCl, 2 mM MgCl_2_, 0.1 mM EDTA, and 1 mM DTT, pH 7.5 and placed into the sample cell of the instrument. The ligand solutions were made up in the dialysis buffer and placed into the injector syringe. Typically 10–30 µM of protein was titrated with 3.2–8 µl aliquots of SAM (1 mM) or SAH (0.5–1 mM). In all cases, heat changes resulting from the titration of buffer with the respective ligands were subtracted from the titration data. Integrated, dilution-corrected and concentration-normalized peak areas of raw data were fitted with the “One binding site” model of the MicroCal version of ORIGIN. The parameters Δ*H* (reaction enthalpy), *K*_A_ (binding constant, *K*_A_  = 1/*K*_D_), and *n* (reaction stoichiometry) were determined from the curve fit. The change in free energy (Δ*G*) and in entropy (Δ*S*) was calculated from the values of *K*_A_ and Δ*H* using the equation Δ*G*  = −*RT* ln *K*_A_  =  Δ*H*−TΔS, where R is the universal molar gas constant and T is the absolute temperature.

#### Analytical ultracentrifugation studies

An Optima XL-I analytical ultracentrifuge (Beckman-Coulter) was used to perform the analytical ultracentrifugation experiments of WspC-Pp (0.25–1.0 mg/ml) in the absence and presence of ligands. The detection was carried out by means of a UV-visible absorbance detection system. Experiments were conducted at 20°C using an AnTi50 eight-hole rotor and Epon-charcoal standard double sector centerpieces (12-mm optical path). Absorbance scans were taken at the appropriate wavelength (280–295 nm). Sedimentation velocity experiments were performed at 48000 rpm using 400 µl samples in buffer consisting of 50 mM Tris/HCl pH 8.0, 300 mM NaCl and 1 µM DTT. Differential sedimentation coefficient distributions, *c(s)*, were calculated by least squares boundary modeling of sedimentation velocity data using the program SEDFIT [59]. From this analysis, the experimental sedimentation coefficients of the proteins were corrected for solvent composition and temperature with the program SEDNTERP [60] to obtain the corresponding standard *s* values. Short column (85 µl) sedimentation equilibrium was conducted at two speeds (12000 and 15000 rpm). Following the equilibrium scans, the solutions were centrifuged at high speed (40000 rpm) to deplete the meniscus and obtain the corresponding baseline offsets. The measured equilibrium concentration (signal) gradients of the proteins were fitted by the equation that characterizes the equilibrium gradient of an ideally sedimenting solute (HeteroAnalysis program, [61]), yielding the corresponding whole cell signal average molecular weights (MW), using 0.732 ml/g as the partial specific volumes of CherR1 (calculated from the amino acid composition using SEDNTERP [60].

## Supporting Information

Figure S1 **Distribution of CheR-TPR sequences in function of protein length.** The lengths of translated proteins were classified into groups comprising 10 amino acids. The relative abundance of sequences in these groups is shown.
(TIF)

Figure S2 **Distribution of CheR-TPR sequences in function of linker length.** The segment between the CheR domain and the TPR domain was determined for each CheR-TPR sequence and its length classified into groups comprising 10 amino acids. The relative abundance of sequences in these groups is shown.
(TIF)

Figure S3 **Three dimensional structure of the CheR methyltransferase from** ***Salmonella typhimurium*** **.** The structure is deposited in the protein data bank with accession code 1af7. A) Structure of the entire protein, bound SAH as well as catalytic residues R98 and Y235 are shown in ball-and-stick mode. B) Zoom of the active site.
(TIF)

Figure S4 **Sequence alignment of a selection of CheR-TPR sequences with the TPR domain on the C-terminal part of the protein (A) and with the TPR domain on the N-terminal part of the protein (B).** In both alignments the sequences of CheR from *E. coli* and *S. typhimurium* have been included. The residues R98 and Y235 (see Figure S3) identified in these latter proteins as catalytic residues are shaded in yellow. Both residues are conserved in (A) but not in (B).
(PDF)

Figure S5 **Relative abundance of TPR genes compared to total number of genes per genome.** Shown are the means and corresponding standard deviations for the different taxa. TPR genes were identified by a search in Pfam using clan CL0020 (Tetratrico peptide repeat superfamily) and an E-value threshold of 1.0 e^−4^. CheR-TPRs are characterized by a detection using InterPro signature IPR000780 (MCP methyltransferase, CheR type).
(TIF)

Table S1 **Presence of CheR-TPR genes in different bacterial genomes.**
(XLS)

Table S2 **Analysis of the genetic environemnt of cheR-TPR genes.** Shown is the architecture of the response regulator (RR) present in CheR-TPR containing clusters.
(XLS)

Table S3 **Analysis of total TPR proteins encoded by the CheR-TPR containing genomes.**
(XLS)

Analysis S1 **Analysis of chemoreceptor-TPR fusion proteins.** Sequences were retrieved from InterPro by a search of the CheR-TPR containing genomes (Table S1) for matches of InterPro signatures IPR011990 (TPR-like helical) and IPR004089 (methylaccepting chemotaxis protein [MCP] signaling domain). Shown are protein sequences and the fragment recognized by IPR011990 is shown in red. The transmembrane regions as predicted by the TMHMM Server v. 2.0 (http://www.cbs.dtu.dk/services/TMHMM-2.0/) are shaded in yellow. The sequence fragment predicted to be located in the cytosol is underlined. Shown are also images from the InterPro output of each sequence. The sequence fragments shaded in red containing 5 additional amino acids at each side were submitted to the three dimensional homology modeling server CPHmodels 3.2 (Nielsen et al. (2010) CPHmodels-3.0 Nucleic Acids Research 38, doi:10.1093/nar/gkq535). Shown are the resulting homology models and the protein database ID of the templates used for modeling. In each case the model showed the typical structure of a TPR domain containing 2–3 TPR. For reference, the three dimensional structure of the TPR domain of pdb entry 2C2L is shown below. This template was used to generate some of the homology models. At the end of this document a sequence alignment of members of the chemoreceptor-TPR family is shown. The alignment was made using the CLUSTALW algorithm of the NPSA server (http://npsa-pbil.ibcp.fr/cgi-bin/npsa_automat.pl?page=/NPSA/npsa_clustalw.html). The GONNET matrix was used and the gap opening and gap extension penalties were set at 10 and 0.1, respectively. The TPR regions are shaded in yellow. Amino acids in red are fully conserved, those in green strongly similar and those in blue weakly similar.
(DOCX)

Analysis S2 **TPR proteins shown or proposed to be involved in non-flagellum mediated motility.** Shown are domain annotations in Interpro and homology models created using CPHmodels 3.2 (Nielsen et al. (2010) CPHmodels-3.0 Nucleic Acids Research 38, doi:10.1093/nar/gkq535).
(DOCX)
