# Supplementary figures and images for: Genes Encoding Cher-TPR Fusion Proteins Are Predominantly Found in Gene Clusters Encoding Chemosensory Pathways with Alternative Cellular Functions
Source: PLoS One. 2012 Sep 20;7(9):e45810. doi: 10.1371/journal.pone.0045810 (PMC3447774; doi:10.1371/journal.pone.0045810)

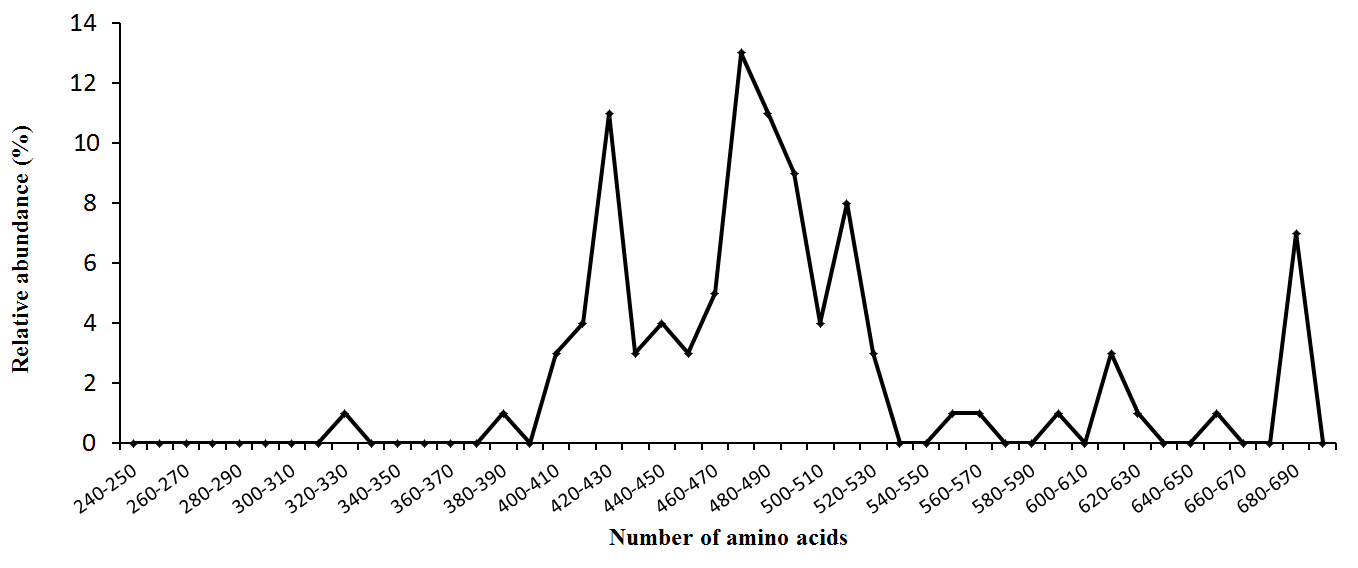

Supplement: Figure S1 — Distribution of CheR-TPR sequences in function of protein length. The lengths of translated proteins were classified into groups comprising 10 amino acids. The relative abundance of sequences in these groups is shown. (TIF) [file pone.0045810.s001.tif]

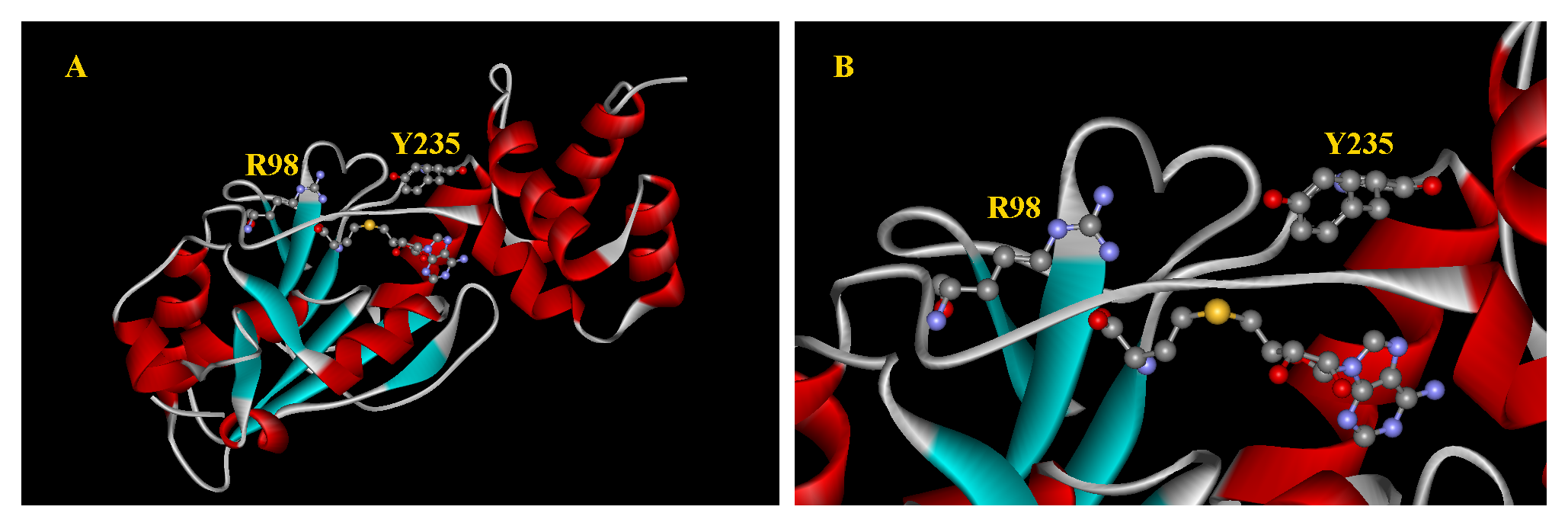

Supplement: Figure S3 — Three dimensional structure of the CheR methyltransferase from Salmonella typhimurium . The structure is deposited in the protein data bank with accession code 1af7. A) Structure of the entire protein, bound SAH as well as catalytic residues R98 and Y235 are shown in ball-and-stick mode. B) Zoom of the active site. (TIF) [file pone.0045810.s003.tif]

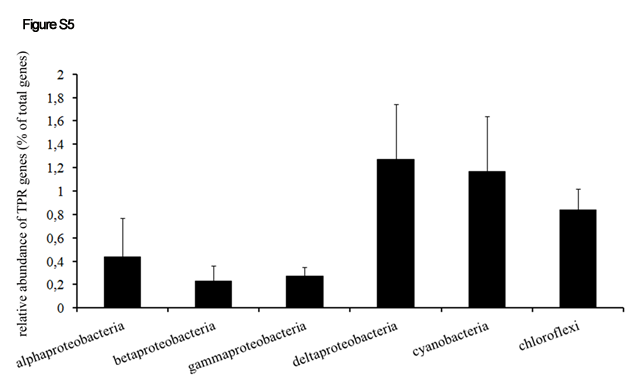

Supplement: Figure S5 — Relative abundance of TPR genes compared to total number of genes per genome. Shown are the means and corresponding standard deviations for the different taxa. TPR genes were identified by a search in Pfam using clan CL0020 (Tetratrico peptide repeat superfamily) and an E-value threshold of 1.0 e−4. CheR-TPRs are characterized by a detection using InterPro signature IPR000780 (MCP methyltransferase, CheR type). (TIF) [file pone.0045810.s005.tif]
