# Supplementary material for: Genes Encoding Cher-TPR Fusion Proteins Are Predominantly Found in Gene Clusters Encoding Chemosensory Pathways with Alternative Cellular Functions
Source: PLoS One. 2012 Sep 20;7(9):e45810. doi: 10.1371/journal.pone.0045810 (PMC3447774; doi:10.1371/journal.pone.0045810)
Supplement: Figure S4 — Sequence alignment of a selection of CheR-TPR sequences with the TPR domain on the C-terminal part of the protein (A) and with the TPR domain on the N-terminal part of the protein (B). In both alignments the sequences of CheR from E. coli and S. typhimurium have been included. The residues R98 and Y235 (see Figure S3) identified in these latter proteins as catalytic residues are shaded in yellow. Both residues are conserved in (A) but not in (B). (PDF) [file pone.0045810.s004.pdf]

Figure S4A

```
                     10        20        30        40        50        60        70        80        90       100       110       120
                      |         |         |         |         |         |         |         |         |         |         |         |
E. coli      ------------------------------------------------------------------------------------------------------------------------
S. typhi     ------------------------------------------------------------------------------------------------------------------------
Q12AR4       ------------------------------------------------------------------------------------------------------------------------
Q2LR61       ------------------------------------------------------------------------------------------------------------------------
Q2YC83       ------------------------------------------------------------------------------------------------------------------------
B8FNR6       ------------------------------------------------------------------------------------------------------------------------
B5ECK8       ------------------------------------------------------------------------------------------------------------------------
C6DYF7       ------------------------------------------------------------------------------------------------------------------------
A9WGY5       ------------------------------------------------------------------------------------------------------------------------
B9LL63       ------------------------------------------------------------------------------------------------------------------------
A7NNV5       ------------------------------------------------------------------------------------------------------------------------
A5URZ9       ------------------------------------------------------------------------------------------------------------------------
Q2W5V8       ------------------------------------------------------------------------------------------------------------------------
D3NZL7       ------------------------------------------------------------------------------------------------------------------------
C7C9P3       ------------------------------------------------------------------------------------------------------------------------
B7KRG4       ------------------------------------------------------------------------------------------------------------------------
A9W6M0       ------------------------------------------------------------------------------------------------------------------------
C5AVV0       ------------------------------------------------------------------------------------------------------------------------
B1ZEP6       ------------------------------------------------------------------------------------------------------------------------
B1LWP1       ------------------------------------------------------------------------------------------------------------------------
B7JYH8       ------------------------------------------------------------------------------------------------------------------------
E0UB22       ------------------------------------------------------------------------------------------------------------------------
C6DZA2       ------------------------------------------------------------------------------------------------------------------------
B0TG84       ------------------------------------------------------------------------------------------------------------------------
A5G943       ----------------------------------------------------------------MSLHILVINENEALCDFLSHLLSEDGHEVSCVRGYREALQAARSERPELIILEVST
B9LZW0       ----------------------------------------------------------------MPLQILVINENKAFCDYLVNLLATAGHEVSCVQLYADALQAAAANRPAAIILEIST
B5EF82       ----------------------------------------------------------------MPLQILLISDNASFADYLNHLLGEAGHAVTTLDDPSQGFREVRRQKPELIILAVEP
C6E726       ----------------------------------------------------------------MPLQILLISDNASFADYLNHLLGEAGHAVTTLHDPSQGFRAVRRQKPELIILAVEP
A9WBT1       ------------------------------------------------------------------------------------------------------------------------
B9LEA3       ------------------------------------------------------------------------------------------------------------------------
B8G3A2       ------------------------------------------------------------------------------------------------------------------------
A7NRP0       ------------------------------------------------------------------------------------------------------------------------
A5UPC3       ------------------------------------------------------------------------------------------------------------------------
B7K4M9       ------------------------------------------------------------------------------------------------------------------------
E0UAE6       ------------------------------------------------------------------------------------------------------------------------
B2J4R2       ------------------------------------------------------------------------------------------------------------------------
B1Z0W8       ------------------------------------------------------------------------------------------------------------------------
Q0B461       ------------------------------------------------------------------------------------------------------------------------
Q1BLP9       ------------------------------------------------------------------------------------------------------------------------
A0AYP9       ------------------------------------------------------------------------------------------------------------------------
B1K4W3       ------------------------------------------------------------------------------------------------------------------------
A9AP36       ------------------------------------------------------------------------------------------------------------------------
A4JLK2       ------------------------------------------------------------------------------------------------------------------------
A1UYE3       ------------------------------------------------------------------------------------------------------------------------
A2S0C7       ------------------------------------------------------------------------------------------------------------------------
A3MB25       ------------------------------------------------------------------------------------------------------------------------
Q3JJX9       ------------------------------------------------------------------------------------------------------------------------
A3P8B0       ------------------------------------------------------------------------------------------------------------------------
A3NMV6       ------------------------------------------------------------------------------------------------------------------------
Q2T7Z6       ------------------------------------------------------------------------------------------------------------------------
B2JNG9       ------------------------------------------------------------------------------------------------------------------------
B2T7T3       ------------------------------------------------------------------------------------------------------------------------
D8J0F2       ------------------------------------------------------------------------------------------------------------------------
A6STX7       ------------------------------------------------------------------------------------------------------------------------
Q0JZJ6       ------------------------------------------------------------------------------------------------------------------------
B3RBS6       ------------------------------------------------------------------------------------------------------------------------
Q46V25       ------------------------------------------------------------------------------------------------------------------------
Q1LG87       ------------------------------------------------------------------------------------------------------------------------
Q2KV72       ------------------------------------------------------------------------------------------------------------------------
B7V0Y7       ------------------------------------------------------------------------------------------------------------------------
Q02RH8       ------------------------------------------------------------------------------------------------------------------------
A6V180       ------------------------------------------------------------------------------------------------------------------------
Q88MS8       ------------------------------------------------------------------------------------------------------------------------
A5W896       ------------------------------------------------------------------------------------------------------------------------
B0KRK9       ------------------------------------------------------------------------------------------------------------------------
Q1IDW8       ------------------------------------------------------------------------------------------------------------------------
Q98PC8       ------------------------------------------------------------------------------------------------------------------------
D8FCI6       ------------------------------------------------------------------------------------------------------------------------
C6BU38       ------------------------------------------------------------------------------------------------------------------------
Q60AM0       ------------------------------------------------------------------------------------------------------------------------
A0L7P7       ------------------------------------------------------------------------------------------------------------------------
Q2W2W8       ------------------------------------------------------------------------------------------------------------------------
Q1NKB1       ------------------------------------------------------------------------------------------------------------------------
Q1NWT4       ------------------------------------------------------------------------------------------------------------------------
Q1NLW5       ------------------------------------------------------------------------------------------------------------------------
Q1D4W0       ------------------------------------------------------------------------------------------------------------------------
E3FYQ2       ------------------------------------------------------------------------------------------------------------------------
A7H826       ------------------------------------------------------------------------------------------------------------------------
Q1D226       ------------------------------------------------------------------------------------------------------------------------
Q08NE7       ------------------------------------------------------------------------------------------------------------------------
Q1D8X4       ------------------------------------------------------------------------------------------------------------------------
Q08UZ6       ------------------------------------------------------------------------------------------------------------------------
A7H7I5       ------------------------------------------------------------------------------------------------------------------------
Q1CX10       ------------------------------------------------------------------------------------------------------------------------
E3FZL3       ------------------------------------------------------------------------------------------------------------------------
A7HCQ0       ------------------------------------------------------------------------------------------------------------------------
E3FRV5       ------------------------------------------------------------------------------------------------------------------------
Q08UV4       MIQFAPVSEREPDCINVRSHDVRRCRRGTLPHRARAGLDLRAAHPGGRRDDAAVAGDAGGGCAALCPGRGRGARPSVPRHPSGDLAGGLQRGHGPALRLAARGGAAARAGGGGSAGAAAA
A7HCX6       ------------------------------------------------------------------------------------------------------------------------
C6C1V4       ------------------------------------------------------------------------------------------------------------------------
D2Z5P5       ------------------------------------------------------------------------------------------------------------------------
D0LTR4       ------------------------------------------------------------------------------------------------------------------------
Q08XU2       ------------------------------------------------------------------------------------------------------------------------
A0L8P6       ------------------------------------------------------------------------------------------------------------------------
D0LUD0       ------------------------------------------------------------------------------------------------------------------------
D3PC59       ------------------------------------------------------------------------------------------------------------------------
D3P9S2       ------------------------------------------------------------------------------------------------------------------------
```

```
                     130       140       150       160       170       180       190       200       210       220       230       240
                       |         |         |         |         |         |         |         |         |         |         |         |
E. coli       -------MTSSLPCG--------QTS------------------------LLLQMTERLALS--------------DAHFRRISQLIYQRAGIVLADHKRDMVYNRLVRRLRSLGLT-DF
S. typhi      -------MTSSLPSG--------QTS------------------------VLLQMTQRLALS--------------DAHFRRICQLIYQRAGIVLADHKRDMVYNRLVRRLRALGLD-DF
Q12AR4        -------------------------------------------------------MHGQVMP---------------AQWSQLSELVAGTMGLHFPRERWDDLKRGLIDAAQEFGFEDAA
Q2LR61        -------------------------------------------------------MDRFISD---------------EQWRRLAEWITFHMGLHMPRERLPDLKRRLLSAAREFHFDSIE
Q2YC83        -------------------------------------------------------MQITLPH---------------VLVSQLSDLIAMKMGLYYPQKRWGDLERAIAAAAPELDMPDTE
B8FNR6        -------------------------------------------------------MAQNIEK---------------NLFARLSELIQNKIGLFFPPERWHDLERGVRAAALELGFEDTT
B5ECK8        --------------------------------------------------------MSAHSN---------------EAMHKFAHMVTTKLGLHFPPERLAELARKMAPLAKDAGWEELD
C6DYF7        --------------------------------------------------------MSAHSN---------------AAMHKFARMVTIKLGLHFPPERLAELARKMAPLAKGAGWEELD
A9WGY5        ----------------------------------------------------------MLML-------------SDELYHRFATLLRQRAGLCYPEQRRADLTHGLQRLALHLGVE---
B9LL63        ------------------------------------------------------------ML-------------SDELYHRFATLLRQRAGLCYPEQRRADLTHGLQRLALHLGVE---
A7NNV5        ---------------------------------------------------------MNTTF-------------SDDLFRRFRDLLLTRSGLYFPDHRRSDLHHCLTMVLPATGYA---
A5URZ9        ---------------------------------------------------------MSTTL-------------TSDLFRRFRDLLLTRSGLYFPDHRQNDLHHCLMSVLPATGCT---
Q2W5V8        ----------------------------------------------MSLPSSGAPPAVAALL-------------DDPAYSVLKALIIDTTGMAFYRDKDSDLARILAERMAEIGIG---
D3NZL7        ---------------------------------MAGCRHGRGCGQGWCRMMSSAAAGIEAIR-------------RDPHFPRLKAMVIDATGLAYYADKDAAFAERINRRLPFGRIT---
C7C9P3        -----------------------------------------------MTAPIERSRRVASAT-------------VDPGFPALKDRVIARTGHNYYEDKDDLLYDRLRKRMRSRGVA---
B7KRG4        -----------------------------------------------MTAPVERSRRVASAT-------------VDPGFPALKDRVIARTGHNYYEDKDDLLYDRLRKRMRSRGVA---
A9W6M0        -----------------------------------------------MTAPVERSRRVASAT-------------VDPGFPALKDRVIARTGHNYYEDKDDLLYDRLRKRMRSRGVA---
C5AVV0        -----------------------------------------------MTAPVERSRRVASAT-------------VDPGFPALKDRVIARTGHNYYEDKDDLLYDRLRKRMRSRGVA---
B1ZEP6        -----------------------------------------------MTDSAERARRAAGAM-------------ADPGFPALKERVIARTGHNYYEDKDDLLYDRLRKRMRMCGVG---
B1LWP1        -----------------------------------------------MARPGNPRPDLG----------------VDPAYPPLKARIIARTGHHYYIDKDELLIERLHRRFRAAAVA---
B7JYH8        -------------------------------------------------------MAKKLGF-------------TSQLREAFLGLISQKTGLEIRKQNQDVLQENILARTQTLKLH---
E0UB22        -------------------------------------------------------MTTSEFL-------------SSELKQEFIDLIAKQTGIEIREQNYAGLSENIYSRLKTLKLD---
C6DZA2        ------------------------------------------------------------MV-------------VMTLLRDFMELVATRTGLNFRDRDQAVFRNKVQERITRYRG----
B0TG84        ---------------------------------------------------------------------------MACGMAELQQLISERTGWRFDQSRRDNLLQVLQARMEAHGIA---
A5G943        TAIGAVETADRLHRAEESRHVPVIVISDYPELEFELLNLFDFILTPVDTARLREDIETIAQGGKKRSLPSQSEPLSEQDYLLYYEYLLGHSGLHFERRNLKILERGLIKRMSALKID-SY
B9LZW0        SDMGAVEMISRLQRAVETRHVPIIVISDVPELEFELLNLFDFLPQPLDTVRLAEDLEAIARG-RKQSLPRQTETLGDDTYRLYSQYLLQESGLHFDRRNQKLLERGLLNRMAALKIS-SF
B5EF82        AKIAPLAVEHRVHTP-KPRVIPVIVISECLRLEAELLHVFDFIPKPLQIKRLFDDLTSLAQR---SAPSCTPHEIDDDLCCEFSKHILSCTGLHFEQRNRAALLRGLAKRMHALRIG-SY
C6E726        AKLAPLAVEHRVHTP-EPRVIPVIVISECLRLEAELLHVFDFIPKPLQIKRLFDDLTFLSQQ---NAPSCTQHEIDEELCCVFSKHILSCTGLHFEQRNRAALLRGLAKRMYALRIG-SH
A9WBT1        -------------------------MT---------------------------------LS--------------TEALVPLRDLLARYCGVLLDDARLVTLRSAVERRAAAAAR--TP
B9LEA3        -------------------------MT---------------------------------LS--------------TEALVPLRDLLARYCGVLLDDARLVTLRSAVERRAAAAAR--TP
B8G3A2        -------------------------MT---------------------------------LP--------------PESLGSLREILAHYCGILLDDSRLTHLQTAVERRALALKR--LP
A7NRP0        MRTNGMNMAHDLFVP--------PPVR---------------------------------LS--------------PEAFDRLRTLLADYSGVYLDTAQQRVLEAGLAQRVAALGE--TL
A5UPC3        -------------------------MR---------------------------------LS--------------QEAFDRLRNLLADYSGVYLDTAQQRVLESGLAQRLAALEE--TL
B7K4M9        -------------------------------------------------------MNSEQTE--------------------IARLLKERVGLEESKIGTRQLSKAIANRQKACGCN-DL
E0UAE6        -------------------------------------------------------MTPEEKQ--------------------IEALLQQKIGLKKDSINARQITNALENRRLACDLP-DL
B2J4R2        ---------------------------------------------------------MVLSA--------------------IATLLNQAIGLDPDTLGSNRIARAVENRRLGCKLP-DM
B1Z0W8        -------------------------------------------------------MKESDS------------------R--FRGWLLRETGIDPDSLGNDFLSRALTERVHALQG--DG
Q0B461        -------------------------------------------------------MKESDS------------------R--FRGWLLRETGIDPDSLGNDFLSRALTERIHALQG--DG
Q1BLP9        -------------------------------------------------------MNMSES------------------R--FRGWLLRETGIDPDSLGNDFLMRALADRVHALQA--DG
A0AYP9        -------------------------------------------------------MNMSES------------------R--FRGWLLRETGIDPDSLGNDFLMRALADRVHALQA--DG
B1K4W3        ---------------------------------------------------------MSES------------------R--FRGWLLRETGIDPDSLGNDFLMRALADRVHALQA--DG
A9AP36        -------------------------------------------------------MRDFDS------------------R--FRGWLLRETGIDPDSLGNDFLIRAVTERVHAVLAA-DG
A4JLK2        -------------------------------------------------------MNAFAV------------------L--FRDWLRRETGIDPDSLGHDFLSRALTERVHALKS--DG
A1UYE3        -------------------------------------------------------MNVFDP------------------R--FHAWLSRETGIDPASLGNDFVARALAERIAATQPG-AG
A2S0C7        -------------------------------------------------------MNVFDP------------------R--FHAWLSRETGIDPASLGNDFVARALAERIAATQPG-AG
A3MB25        -------------------------------------------------------MNVFDP------------------R--FHAWLSRETGIDPASLGNDFVARALAERIAATQPG-AG
Q3JJX9        -------------------------------------------------------MNVFDP------------------R--FHAWLSRETGIDPASLGNDFVARALAERIAATQPG-AG
A3P8B0        -------------------------------------------------------MNVFDP------------------R--FHAWLSRETGIDPASLGNDFVARALAERIAATQPG-AG
A3NMV6        -------------------------------------------------------MNVFDP------------------R--FHAWLSRETGIDPASLGNEFVARALAERIAATQPG-TG
Q2T7Z6        -------------------------------------------------------MNVFDP------------------R--FHAWLSRETGIDPASLGNDFVARALAERIAATQPG-AG
B2JNG9        ----------------------------------------------------MNAANAFMP------------------R--FEAWLLRETGIDASSLGVAALERAVLERVRVAWIG-AD
B2T7T3        -------------------------------------------------------MNAHHDNAP------------LYRR--FAELLHRTIGLDAASLGHSAIERAVDQRAAAWCA--DG
D8J0F2        -------------------------------------------------------MKSLQA---------------------IADKLKAAMGLDMATVGASLIERVVRERMAVLEME-DD
A6STX7        -------------------------------------------------------MTTLNR---------------------IASLLRHSMGLDVASVGMMLIERAVSERMLTLGMT-QD
Q0JZJ6        -------------------------------------------------------MSTATH---------------------IEALLKARIGLDAGAIGTGAVARAVRERQQARQAP-DT
B3RBS6        -------------------------------------------------------MSTAAH---------------------IEALLKARIGLDAGAIGSGAVARAVRERQQARQAP-DT
Q46V25        -------------------------------------------------------MSTATH---------------------IEALLRARIGLDARSIGPGALERALRERQAALQAH-DE
Q1LG87        -------------------------------------------------------MTDAPHP-----------------STLIEALLKERIGLDAASVGRGTIERAVEERRQAMGAD-DP
Q2KV72        -------------------------------------------------------MIQPDFP----------------------ELLKRRIGLDTACIGEAAVSRAVRLRMQASGVD-DE
B7V0Y7        -------------------------------------------------------MN-DRFE-----------------R-----LLKSRIGLDASSVGSAVIERAVRQRMSGLALH-DE
Q02RH8        -------------------------------------------------------MN-DRFE-----------------R-----LLKSRIGLDASSVGSAVIERAVRQRMSGLALH-DE
A6V180        -------------------------------------------------------MN-ERFE-----------------R-----LLKSRIGLDASSVGSAVIERAVRQRMSSNALH-DE
Q88MS8        -------------------------------------------------------MNEQRFF-----------------R-----FLRERIGLDVESVGAPMVERALRQRCVAAGAM-DL
A5W896        -------------------------------------------------------MNEQRFF-----------------R-----FLRERIGLDVESVGAPMVERALRQRCVAAGAM-DL
B0KRK9        -------------------------------------------------------MNEQRFF-----------------R-----FLRERIGLDVESVGAPMVERALRQRCVAAGAM-DL
Q1IDW8        -------------------------------------------------------MSEQRFF-----------------R-----FLRERIGLDVESVGAPMIERALQQRSIAVLAT-DL
Q98PC8        -------------------------------------------------------MTGAIET-----------------------LLTETIGLDSTSVGPTVVRQAIKDRMAACRIT-NP
D8FCI6        -------------------------------------------------------MKNNTVES----------------------LIEEKIGLDAEAIGRKLVEKAVSARMEQCRIS-SV
C6BU38        -------------------------------------------------------MNVAPFLK----------------------ILNRAMGLAPDSLSRSGIELALRARMRETATT-EK
Q60AM0        -------------------------------------------------------MRLNGDVG-------------------WLWDWLAAAGLEPRALGEAAVLCALKRRLDAAGCT-AA
A0L7P7        -------------------------------------------------------MDHAVLHP-------------------LMALIKQRTGLQLD---NDSLLFKLVQQGCLAHRC-TV
Q2W2W8        -------------------------------------------------------MNAAELAP-------------------FKDLIRTRCGLLLEGVGEEPLVIAIRKRMAEAGVA-GL
Q1NKB1        -----------------------------------------------------MNPQLNDITR-------------------FKQLLLTGCGLQFDDLRERTLREAVGRRISHRQAA-GA
Q1NWT4        -----------------------------------------------------MNPQLNDITR-------------------FKQLLLTGCGLQFDDLRERTLREAVGRRISHRQAA-GA
Q1NLW5        -----------------------------------------------------MNPQLNDITR-------------------FKQLLLTGCGLQFDDLRERTLREAVGRRISHRQAA-GA
Q1D4W0        -------------------------------------------------------MLTA----------------SQKVLQQLAALLLERAGLKITPDGFHSLRLALSARMPVLG--LEE
E3FYQ2        -------------------------------------------------------MLNV----------------SQKALQQLAALLLERAGLKITPDGYHSLRLALSTRMPVAG--ISD
A7H826        -------------------------------------------------------MRPI----------------TPDDLSQLAALLKERVGLHVRQDGHSALRLAVGARLDELGGAVPD
Q1D226        -------------------------------------------------------MT------------------DGAPWSGVRAFLSARTGMALSGPQVRRLDERLAARSQGLT---PH
Q08NE7        -------------------------------------------------------MQGP----------------SSQPASLLHAFISARSGMALSGTQRRRLDERLALLQGSLT---EQ
Q1D8X4        -------------------------------------------------------MSEGV--------------LDDATLARVEDVLQSACGVTLARSLRRSLETALGRAARSRGLEPDA
Q08UZ6        -------------------------------------------------------MNEVVRAD----------ALDEATLSRVEDVLREASGLTLTASVRRSLSTALVRAAEARKLAPPV
A7H7I5        -------------------------------------------------------MTATG-------------APSAAGVEDVERALRDACGITLTEGLRTTLAEALRRAARATGLDPDT
Q1CX10        -------------------------------------------------------MNWGP--------------WSHPGYAAVLALVEERAGLVPPSCP-ASAEEGIARAMERAGLKDFD
E3FZL3        -----------------------------------------------------MSLEPAP--------------WSRLGYAHIFAHVQQRAGLQLPTCI-PAAEEGIARAMARAGEEDLG
A7HCQ0        -------------------------------------------------------MTLRP--------------AQAPATERLRALVREQAGLDLSGVRGTLLAAALTREAHAGGLPDGE
E3FRV5        -------------------------------------------------------MEWPKAVNR------------------FATFVERRLGLAPVPNGGRELEALLAEKSARSG-----
Q08UV4        GRARAGHAAAAAGGGRGGQPGGARHAGWAIAGGAGHLAAVDRSGLGPVGDGGGGVMEWPKAVNR------------------FATFVERRLGLAPVPNGGRELEALLAEKSARSG-----
A7HCX6        ----------------------------------------------------MTARDRTEQLER------------------FRRAVGARLGLRFEDDKLELLAEVAAERVEATGAR--S
C6C1V4        ------------------------------------------------------MINFLTDDR----------------VSELADMVRNRFGLNFSSERWKDLRSAVVRFQREDSSFKTP
D2Z5P5        -------------------------------------------------------MSEAFGR------------LSDPDLDTVVSLLSRRFGLVMGWDEIKARDKAASLIGELSRKLGET
D0LTR4        -------------------------------------------------------MSEPPPVP-----------LESELRGAFATLLEQQFGLVFPSSRQPLIDDGVRAALRRLDESAPR
Q08XU2        -------------------------------------------------------MTVPGG-------------LDPLLFARARQLVSERTGFRDDAIAPESLERVVRAELARGRT----
A0L8P6        -------------------------------------------------------MATALAD----------------DERALAQLVRQRLGITVRDHQILPFRRGIQQTLDRFGLS---
D0LUD0        -------------------------------------------------------MHDP-------------------LWHTLAALFHGWTGMLVPDTMRATAVYELTRMAAQRGRE---
D3PC59        -------------------------------------------------------MNSENFN-------------------MLCEKIEKTSGFRVYGLYEDNLKNIVSNYAKQMMLN---
D3P9S2        -------------------------------------------------------MTINYSEN-------------------IKSLVKSFSGLNLTGSIEEKFNETLSKWLQHYKTP---
```

```
                  250       260       270       280       290       300       310       320       330       340       350       360
                    |         |         |         |         |         |         |         |         |         |         |         |
E. coli     GHYLN----------------LLESNQHS-----GEWQAFINSLTTNLTAFFREAHHFPLLADHAR------------RRSGE---YRVWSAAASTGEEPYSIAMTLADTLGT-APGR-W
S. typhi    GRYLS----------------MLEANQNS-----AEWQAFINALTTNLTAFFREAHHFPILAEHAR------------RRHGE---YRVWSAAASTGEEPYSIAITLADALGM-APGR-W
Q12AR4      A----------------CIDWLLSAPPTRA-----QLQVLAGHLTVGETYFFRDRQTLEVLTESILPELIRARRGG-------EQRLRIWSAGCCSGEEPYSLAILLHEALPDFG-NWQV
Q2LR61      P----------------FVSWLLSTKLTQR-----QIETLASHLTVGETYFFRETASLEALEKYILPELIESRRS--------TRSLRVWSAGCATGEEPYTIAIMLSRLIPDIS-KWQV
Q2YC83      T----------------AARHLLSTSLTRR-----QIEILACRLTVGETYFFREQKCLNVLEQSILPELIRKCKES-------NRQLRIWSAGCCTGEEPYSIAILLDRLFEKHGEEWNA
B8FNR6      S----------------CIQGLLGSPVTKV-----QLDTLVAHLTVGETFFLRDESHFNALQHQILPDIFEKCAKK-------GKPPRFWSAACCTGEEPYTMAMVVDRMKPYWT-AKDF
B5ECK8      G----------------YLHWLMSQPLSLE-----QTKALALALTIGETYFLRDPKSYRAFQQHLLPKLLAAKGS--------DKSLKIWSAGCSSGEEPYSIAILLTRALADLA-DWKV
C6DYF7      G----------------YLHWLMSQPLSQE-----QTKALALALTIGETYFLRDPKSYRAFQQHLLPKLVAAKGS--------DKTLRIWSAGCSSGEEPYSIAIMLTRALADLA-DWKV
A9WGY5      -----------------SFAQLLAMVE----SRPSAWDELIAELTIGETYFFRNAAQFAALRDIILPDLMQRRA--------TMRYLRLWSAGCATGEEPYSLAITLHEVLPA-NPPWQV
B9LL63      -----------------SFAQLLAMVE----SRPSAWDELIAELTIGETYFFRNAAQFAALRDIILPDLMQRRA--------TMRYLRLWSAGCATGEEPYSLAITLHEVLPA-NPPWQV
A7NNV5      -----------------TLEALYAAAV----VDDKTLHTVIEGVTVGETYFFRNTSQFTALRDRILPDLIARRE--------VIRSLRFWSAGCATGEEPYSLAILLHEALPD-PDAWQI
A5URZ9      -----------------SLEALYTAAV----ASERVLHTIIESVTVGETYFFRNASQFAALRDRIIPDLIARRE--------AIRSLRFWSAGCSTGEEPYSLAILLRDTLPD-FDNWQI
Q2W5V8      -----------------DCAGYLDRLHC-LGQGKAEMDALIAELTIGETYFFRHKEQFDALRDLILPEAIERNR--------TVRRLRIWSAGCATGPEPYSVAIMLEREFGERIAGWHV
D3NZL7      -----------------TVGGYLAALKA-EGPGGAEYQGLINELAVGETFFFRYIEQFDALRAVAIPECLRRNQ--------GSRLLRIWSAGCSIGPEAYSVEILLKQQFAVQLEGWQV
C7C9P3      -----------------DCAAYSSLLD--GAAGREEWAALEAEITIGETFFFRYAEQFAALRKTILPELIEARS--------EIRRLRIWSAGCSTGAEPYSLAILVHDLLGEALADWSV
B7KRG4      -----------------DCAAYSSLLD--GAAGREEWAALEAEITIGETFFFRYAEQFAALRKTILPELIEARS--------EIRRLRIWSAGCSTGAEPYSLAILVHDLLGEALADWSV
A9W6M0      -----------------DCAAYSSLLD--GAAGREEWAALEAEITIGETFFFRYAEQFAALRKTILPELIEARS--------EIRRLRIWSAGCSTGAEPYSLAILVHDLLGEALADWSV
C5AVV0      -----------------DCAAYSSLLD--GAAGREEWAALEAEITIGETFFFRYAEQFAALRKTILPELIEARS--------EIRRLRIWSAGCSTGAEPYSLAILVHDLLGEALADWSV
B1ZEP6      -----------------DCTAYRVLLE--GAAGRDEWAALEAEITIGETFFFRYAEQFAALRKSILPELIAARS--------EIRRLRIWSAGCSTGAEPYSLAILVHDLLGEALAEWSV
B1LWP1      -----------------DCAAYAALLSS-GAEGEAEWARLEAEITVGETFFFRYAEQFNALRATILPALIAARA--------SERVLRIWSAGCSTGAEPYSLAILVHELLGDARSDWRV
B7JYH8      -----------------TVEDYYHLLTSETVQSQKEWKQLITLLTNNESYFFRDLGQFKLLRNTILPELIERNQ--------RTKILRICSAGCSTGPEPYSLAILLKELIPN-WQQWNL
E0UB22      -----------------SPQAYYYLLATPNHNSDQEWQQFVCLMTNKESYFFRDKGQFILLRNKILPELIQRKQ--------KSQHLRILSAGCSTGQEPYSLAILLRQMIPD-FAGWHI
C6DZA2      -----------------SEWDYYELLQDETPESAREWCVLAALLTTGESYFFRDKGQFALLKNHILPELIKRK---------RQRRLRIWSAGCSSGEEPYSLAMVVKELLPV-LDNWQI
B0TG84      -----------------DHEAYHRLLLDKGPAGTEEWRRLIDAVTVGETFFFRDRGQFALLRERILPTLLAERR--------DKKALRLWSAGCSTGEEAYSLAILVDELVGA-APGWTI
A5G943      REYYD----------------YLILHQERR----QELKKLLPFLTIGETYFFRYHAHFAALRKLLLD-ELAT------KKPGEKIKLRLWSAGCSTGEEPYSMAMTIMETVPG-WQDMDI
B9LZW0      EEYYH----------------YLVLHQESR----QELKKLLPFLTIGETYFFRYHAHFEALGQILAE-KRSG------LAQGERLKLRLWSAGCSTGEEPYSLAITVMEAISD-WKKHDI
B5EF82      RQYLA----------------YLKLHGEDR----HELQKLLQFLTVGETYFFRYPAHFAALRERFIP-PPPV------DRP-----IRIWSAGCSTGEEPYSIAITLMEALPD-WRDRDI
C6E726      RDYLA----------------YLKLHGEDR----HELQKLLQFLTVGETYFFRYPAHFAALRERFNP-PPPV------DRP-----IRIWSAGCSTGEEPYSIAITLMEALPD-WRDRDI
A9WBT1      ARYLA----------------DLVLDADR-----TELQQLAELLLNHETIFFRNRPHMDALRKTLLP-SLHA------TLPPGM-PIKIWSAGCATGEEPYSIAITALEALGD-PLPRPV
B9LEA3      ARYLA----------------DLVLDADR-----TELQQLAELLLNHETIFFRNRPHMDALRKTLLP-SLHA------TLPPGM-PIKIWSAGCATGEEPYSIAITALEALGD-PLPRPV
B8G3A2      DQYLA----------------DLLTNRDR-----TELQFLAEPFLNHETIFFRNRPHMEALRKTLLP-TLHA------TLPPGV-PIKIWSAGCATGEEPYSIAITALETLGE-PLPRPV
A7NRP0      ESYER----------------HISAPAGR-----NELHRLAEMVVNHETFFFRNAPQMRALRETLLF-ELHR------RKPPGE-PIRIWSAGCATGEEAYSLAITVLETFGL-ALIRPV
A5UPC3      EAYER----------------RITAPAGR-----DELQRLTELVVNHETCFFRNAPHMKALRDTLLF-EMHR------RKPAGE-PIRIWSAGCATGEEAYSLAITALETFGS-TMIRPV
B7K4M9      -------------------PSYLKHLERSQ----REFQELVEEIVISETWFFRHYEAFNLLRKYVRS-EWLP--------NHPQEKLRVLSLPCSTGEEAYSIAMTLFEVPLS-PHQ--F
E0UAE6      -------------------KSYVQFLETSS----QEWWELIDQIVVPETWFFRHREAFKFLHKYVKT-EWLKPKCRTRSAPQPVSILRVLSLPCSTGEEPYSIAITLLEAGLT-SNQ--F
B2J4R2      -------------------ESYWVRLQTST----LELEELIELLIVPETWFFRDGKPFDYLKTYVTD-QWRL--------LPNRNILQLLSVPCSTGEEPYSMAIALLEAGLH-PKQ--F
B1Z0W8      ERLPSAARTP---VTPEALDAYWQHLNASA----DERRALIELFVVPETWFFRDREAFATLARLALE-RIAA--------T-PGRVIRVLSAPCSTGEEPYSAAMALLDAGLD-PAS--F
Q0B461      ERLPSAARPA---VTPEALDAYWQQLNASA----DERRALIELFVVPETWFFRDREAFATLARLALE-RLAA--------T-PGRVIRVLSAPCSTGEEPYSAAMALLDAGLD-PAS--F
Q1BLP9      ERVPSAARPP---VTPEALDAYWQQLNASA----DERRALIELFVVPETWFFRDREAFATLARLAAE-RLAA--------M-PGRVIRVLSAPCSTGEEPYSAAMALLDAGLE-PAS--F
A0AYP9      ERVPSAARPP---VTPEALDAYWQQLNASA----DERRALIELFVVPETWFFRDREAFATLARLAAE-RLAA--------M-PGRVIRVLSAPCSTGEEPYSAAMALLDAGLE-PAS--F
B1K4W3      ERVPAAARPP---VTPEALDAYWQQLNASA----DERRALIELFVVPETWFFRDREAFATLARLAAE-RLAA--------M-PGRVIRVLSAPCSTGEEPYSAAMALLDAGLE-PAS--F
A9AP36      ERLPSSVRPP---VTPEALDAYWQRLNGCA----DERRALIELFVVPETWFFRDREAFATVARLAVQ-RVAA--------S-PSRVVRVLSAPCSTGEEPYSVAMALLDAGLE-PDD--F
A4JLK2      ERLPSAARAP---VTTEALRAYWARLHACA----DERRALIELFVVPETWFFRDGAAFAALARRAAE-RIAC--------A-PGRVIRVLSAPCSTGEEPYSAAMALLDAGLD-PAS--F
A1UYE3      -GEAAQAGRPQPAITDEAVDAYWQCLNASA----DERRALIELFVVPETWFFREREAFAALARLGAQ-RLFA--------E-PARALRILSAPCSTGEEPYSAAMALLDAGID-PSR--F
A2S0C7      -GEAAQAGRPQPAITDEAVDAYWQCLNASA----DERRALIELFVVPETWFFREREAFAALARLGAQ-RLFA--------E-PARALRILSAPCSTGEEPYSAAMALLDAGID-PSR--F
A3MB25      -GEAAQAGRPQPAITDEAVDAYWQCLNASA----DERRALIELFVVPETWFFREREAFAALARLGAQ-RLFA--------E-PARALRILSAPCSTGEEPYSAAMALLDAGID-PSR--F
Q3JJX9      -GEAAQAGRPQPAITDEAVDAYWQCLNASA----DERRALIELFVVPETWFFREREAFAALARLGAQ-RLFA--------E-PARALRILSAPCSTGEEPYSAAMALLDAGID-PSR--F
A3P8B0      -GEAAQAGRPQPAITDEAVDAYWQCLNASA----DERRALIELFVVPETWFFREREAFAALARLGAQ-RLFA--------E-PARALRILSAPCSTGEEPYSAAMALLDAGID-PSR--F
A3NMV6      -GEAAQAGRPQPAITDEAVDAYWQCLNASA----DERRALIELFVVPETWFFREREAFAALARLGAQ-RLFA--------E-PARALRILSAPCSTGEEPYSAAMALLDAGID-PSR--F
Q2T7Z6      DGAAAPAGRPQQSITDEALDAYWQRLNASA----DERRALIELFVVPETWFFREREAFGALARLGAQ-RLFA--------Q-PARVLRILSAPCSTGEEPYSAAMALLDAGID-PAR--F
B2JNG9      AHDTSVCVDP------ADIDAYWQQLTTSR----DERQALIEALVVPETWFYRDREAYVALARLANE-RLVR--------E-PARLLRVLSLPCSTGEEPYSAAMALLDDGIG-ENR--F
B2T7T3      -------------HAEATLADYWEAAQCSP----SMVQALIETVVVPETWFYRDADAFKALARLAHE-RLAE--------RGNALPLRVLSLPCSTGEEPYTIAMTLLDAGFD-PAQ--L
D8J0F2      AL-------------------YLSLLLSSA----AELQALIELAVVPETWFFRDREAILAVARLASD-KLAA--------Q-PAAVIRILSLPCSTGEEPYSLAMALLDAGLT-PGQ--F
A6STX7      A--------------------YLLALQSAP----GELQELIELVIVPETWFFRDREAILSLARMARQ-QIAE--------K-PDRCVRVLSLPCSTGEEPYSVAMALLDAGVP-SVN--F
Q0JZJ6      -------------------QAYWDLLHGTA----DEFQALIEAVVVPETWFFRHREALLALGRLAAE-RAFA--------D-GRRTLRALSLPCSTGEEPYSIAMAMLDAGVP-AGR--F
B3RBS6      -------------------EAYWQLLQGTA----DEFQALIEAVVVPETWFFRHREALLALGRFAAE-RAFA--------D-GKRTLRLLSLPCSTGEEPYSIAMALLDAGVP-PGR--F
Q46V25      -------------------AAYWNLLHSQP----DEFQALIEAVVVPETWFFRHREALMALGRFAAE-RVFG--------D-HARHLRVLSLPCASGEEPYSIAMALLDAGIP-AAR--F
Q1LG87      -------------------ATYWNLLHAVP----DELQALIESVVVPETWFFRHREALLALARFAAQ-RVFG--------A-GEPMLRILSLPCSTGEEPYSIAMALLDAGIP-GDR--F
Q2KV72      -------------------ASYWTRLLSSE----EEMQQLIEAVVVPETWFFRYPESLKALAGLALR---------------HRGELRILSLPCSTGEEPYSIAMALLDAGLK-PEA--F
B7V0Y7      -------------------DEYWMRLNGSP----GEVQALIEAVVVPETWFFRYPESFTTLARLAFE-RLPS--------LGGGRALRILSLPCSTGEEPYSIVMALLDAGLS-EYL--F
Q02RH8      -------------------DEYWMRLNGSP----GEVQTLIEAVVVPETWFFRYPESFTTLARLAFE-RLPS--------LGGGRALRILSLPCSTGEEPYSIVMALLDAGLS-EYL--F
A6V180      -------------------DEYWMRLNGSP----GEVQALIEAVVVPETWFFRYPESFTTLARLAFE-RLPS--------LGGGRALRILSLPCSTGEEPYSIVMALLDAGLS-EYL--F
Q88MS8      -------------------DDYWLRLQQSA----DEQQALIEAVIVPETWFFRYPESFTALASLAHK-RLAQ--------LAGARPLRLLSLPCSTGEEPYSLAMALFDAGMA-PGA--F
A5W896      -------------------DDYWLRLQQSA----DEQQALIEAVIVPETWFFRYPESFTALASLAHK-RLAK--------LAGARPLRLLSLPCSTGEEPYSLAMALFDAGMA-PGA--F
B0KRK9      -------------------DDYWLRLQQSA----DEQQALIEAVIVPETWFFRYPESFTALASLAHK-RLAQ--------LAGARPLRLLSLPCSTGEEPYSLAMALLDAGIA-PGA--L
Q1IDW8      -------------------DDYWLHLQQSA----EEQQALIEAVIVPETWFFRYPESFGALVGLAHK-RVTE--------LAGTRPLRILSLPCSTGEEPYSIAMALLDGGVS-PAA--F
Q98PC8      -------------------QAYREHLTGSS----QELQELINAVVIPETWFFRDREAFTMLARHARV-HKR------------AQSIKVLSLPCSTGEEAYSVAMAMFDAGFE-AHE--F
D8FCI6      R-------------------AYAEALENSD----DEWKRLIEMVVVPETWFFRNREAFHYLVRFVTD-DWLP--------RGSGRPLSVLSIPCSTGEEPYSIAMALLDAGIQ-EDR--F
C6BU38      -------------------S-YLSLVRASD----EELAELVEEIVVPETWFFRDNKPFELLFETVAG---------------CSDEFRVLSAPCSTGEEPYSIAMTLMGAGLK-N----F
Q60AM0      --------------------AYPARLAADA----EERARLLDAVLVPETWFFREPPAFEALAESAAR-HRLQ--------R-RSVPFHVLSLGCSTGEEPWSIVIALREAGMR-KGD--F
A0L7P7      -------------------PAFLLRLEQDR----EIFYELINRLTINETYFYRESEHLQLLIEHLLP-PLLNQQRS--------EPIRILSVGCSSGEEPYSIAMALTERFGP-DQAHLF
Q2W2W8      -------------------AGYLARLVAAE----DEFQEFVTLLTINETYFYREPEQLMLLVDRLMP-RLLADESAG-------LPITILSAGCSSGEEPYSIAIALLEKFGE-AAARMV
Q1NKB1      -------------------EDYYRLLAADA----GEMQALVELVTINETYFMREPEYLKLLLDRLIP-ELLAASGG--------DGLKIFCAGCSSGEEPYSLAILLDQRYG--SAARHF
Q1NWT4      -------------------EDYYRLLAADA----GEMQALVELVTINETYFMREPEYLKLLLDRLIP-ELLAASGG--------DGLKIFCAGCSSGEEPYSLAILLDQRYG--SAARHF
Q1NLW5      -------------------EDYYRLLAADA----GEMQALVELVTINETYFMREPEYLKLLLDRLIP-ELLAASGG--------DGLKIFCAGCSSGEEPYSLAILLDQRYG--SAARHF
Q1D4W0      PE---------------HYIQRLTG-AGGE----EELRSLLPLVTVGHTEFFRDAKQFRALEKSVLP-DLVSRSRR----E--MRKVSIWSAGCATGEEPYSLAMVLAELGAL-S--LEV
E3FYQ2      PE---------------VYVQRLRD-IGGE----QELRSLLPLVTVGHTEFFRDPKQFRALERFILP-QLLAKARR----E--MRRVCIWSAGCATGEEAYSVAMVMAELGAL-A--VEV
A7H826      AR---------------AYLALLRSPAEGD----EELRRLLPLVTVGKTSFFRDERQFRALE-ALLP-ELVARASG----G--GRRVSIWSAGCATGEEPYSIAMTAESLGAA-P--EHL
Q1D226      -----------------QYLMFLQS-PSGV----TDLEDLIAAVVVNKTDLFRDEVQLAAFRAQVLS-PLVARNR---------RPLRVWSAGCATGEEVATLLVLLAEAGAN----SDS
Q08NE7      -----------------QYLLHLQS-PSGA----GDLASLISVIAVHKTDLFRDEVQLASFRTHVLE-PLVARSSG--------RPLRVWSAGCATGEEVATLLILLDEAGAD----PTS
Q1D8X4      --------------------FLRKLLVREA----AAVECFIEHAVIGETYFFRHPEHLRTLARLAQT-HPAPC-------------FQVWSAGCASGEEPYSIAMALMAEGLP-E--GRF
Q08UZ6      --------------------FLKALLARDP----VVVETFIEYAVIGETYFFRHPEHLRELTRVIAA-HGEP--------------VHIWSAGCATGEEPYSITMALLAAGVP-V--ENI
A7H7I5      --------------------FRARLRARDA----RCVAELVEAAVVGETYFFRHPEQLEVVRQLVLE-RAPRER------P-----LRIWSAGCATGEEPYTLAMLLRDAGRA-G--CRD
Q1CX10      --------------------AYRVRLAEEP----SALDDLLIELTVGETYFFRTPEHFEHLRSVVLP-E-LRER------HGPDHTARMWSAACSSGEEPYSIAALLLGEGWD----EHM
E3FZL3      --------------------LYLGRLASDP----GAFDDLLNELTIGETYFFRTHEHFDFLRHQALP-E-LRRL------RGPEHTVRVWSAGCASGEEPYSLAVLLMEEGYG----HRM
A7HCQ0      --------------------AVARALLEGA----RPLAPFIEELTVGETWFFREPAQWAFIEEVVLP-EGLRRR------EGGVERFRAWSAGCSTGEEPYTLAIVLHDRGVR----ERV
E3FRV5      ---------------LEAYLAKLEAAGNQD----AELRLLAERLTVGETYFFRHLAQLQALVAEVLP-RVQREG----------RPARVLCAGCSSGEEAYSVAILCRENALV-EPER-L
Q08UV4      ---------------LEAYLAKLEAAGNQD----AELRLLAERLTVGETYFFRHLAQLQALVAEVLP-RVQREG----------RPARVLCAGCSSGEEAYSVAILCRENALV-EPER-L
A7HCX6      ---------------LDAYVARLSGEAGRD-----EIRPLAARLTVGETYFFRNANDMEAFSGLVLPERVRRRGA--------DRRLRILSAGCASGEEPYTLAMLVRSAPQL-AGWD-V
C6C1V4      -------------------AECLEYILSPTVG-TDDLEQFINRLTIGETFFFRDTNALRVLEYEILRKISGRGS-------GLNGAARIWSTACSTGEEPYTLAMICRRSGVS------A
D2Z5P5      P-----------------EEVFRLIRKRSD---KAFMDMISPHITVGETFFFRDRKALDAFGREIVPRLASR----------AKRPINVWSAGCSTGEEPYTLAMIMDHSILGLAGG--F
D0LTR4      --------------------ALLERMERDD---AEAWEAMATAMTIGETYFFRDPSHFDLLRLIMRG--APR-----------NRRMRLWSSACANGAEPYSMAILALEEFGAAAAER-V
Q08XU2      ------------------LVDLLTDLQNPE---TPFAQTVVRASLVGETYFYRHPEHFRFVARDGVPGVLRR----------NPSRLRGWSAGCATGEEAYSLASCLMASAPP--G-MPV
A0L8P6      ------------------SYAALTDLIRHASGNDPIMESLVAGVTVGESYFYRDPLQISFLENRFLTPLLKQRK------ALGHPALRIWSAGCSDGQELYTLMLILDRLIPK-HETWQV
D0LUD0      ------------------PLALLAALDGDA----EARQELLDRIGLGTTWFAREQSGIAALVAKLAPMAQRR------------RPLRVWSAGCSSGEEPYTLAMSFADAGVD------A
D3PC59      ------------------PEEIIDLAISDS----KILDKIIEALLINETYFFRHEFQFDMIRKHILPDIKKS-----------KEAIRIWSAGCSNGCEPYSVAILIDKYFSELRDN--V
D3P9S2      -------------------QTVFDLLKNDR----TALFDFLAEITINESFFYRNKSQFTILKEIIDH--IP------------SKTIKILSVGCSNGCEPYTIAMEILEQFGKTDN---I
```

```
                   370       380       390       400       410       420       430       440       450       460       470       480
                     |         |         |         |         |         |         |         |         |         |         |         |
E. coli     KVFASDIDTEVLEKARSGIYRHEELKN-LTPQQLQRYFMRGTGP----HEGLVRVRQELANYVDFAPLNLLAK-QYTVPG----PFDAIFCRNVMIYFDQTTQEILRRFVPL-LKPDGL
S. typhi    KVFASDIDTEVLEKARSGIYRLSELKT-LSPQQLQRYFMRGTGP----HEGLVRVRQELANYVEFSSVNLLEK-QYNVPG----PFDAIFCRNVMIYFDKTTQEDILRRFVPL-LKPDGL
Q12AR4      TITATDINPRFLQKAVAGVYGEWSFRN-APAGLKQHYFHRTEDGH-------YTIVPQIRKLVNFAHLNLAED-AYPSLETDTNAMDVIFCRNVLMYFTPLQTRKVIRNLHHA-LVEGGW
Q2LR61      TLLATDINARSLERASQGIYREWSFRG-DFQWLKDQYFTRTLDGR-------YRISEKIRKMVTFNYLNLATD-TYPSLYNNTNAMDIIFCRNVLMYLEPSLAKRVVGRFYRA-LIDEGW
Q2YC83      AILATDINPVFLDRAAEGLYREWAFRG-TPGWIKERYFKRKKNGL-------FEIVPHIRKRVTFSRLNLVED-VYASQTNGTRAVDVIFCRNVLMYFSSPAVKRIGQGFYRS-LVDYGW
B8FNR6      SILATDINSRFLEKARKGIYTNWSLRN-APNWMVEKYFTVHGNNR-------FEVEKGLKKAIRFTNLNLVDP-VYPSAVTHTEDVNVVFCRNVLMYFNQKLRDTVIERIAWA-LADNGW
B5ECK8      TLLGTDINPQALERARCGTYSKWSFRN-APGWLMN-YFTQLPDGN-------YQIEPHIRKMVRFAHLNLVDP-GEK-TWSQAQGMDFIFCRNVMLYFHPEQIRETVARLHAA-LNDGGW
C6DYF7      TLLGTDINPQALERARCGTYSKWSFRN-APGWLMD-YFTQPPDGG-------YRIEPHIREMVRFAHLNLVDP-GEK-AWSLAQGMDFIFCRNVMLYFHPEQIRETVARLHAA-LNDGGW
A9WGY5      SILATDINRRFLNRAREARYGNWSFRE-TPDDLRDRYFVAE-----PE-KGLWRLRDDIRRTVTFAQLNLAEP-TYPAPHLGIVAFDLIFCRNVMIYFDEETTRQVVQRLYDA-LVPGGW
B9LL63      SILATDINRRFLNRAREARYGNWSFRE-TPDDLRDRYFVAE-----PE-KGLWRLRDDIRRTVTFAQLNLAEP-TYPAPHLGIVAFDLIFCRNVMIYFDEETTRQVVQRLYDA-LVPGGW
A7NNV5      TILATDINTAFLNRARAGVYGAWSFRE-TDPALRDRYFER------LE-GTRWRIRSDLRRQVLFARLNLVED-EYPAVMNGTTMQDVILCRNVLIYFDDDTIRAVVRRLYRA-LTPGGW
A5URZ9      TILATDINAGFLERARAGVYGAWSFRE-TEPTVRDRYFEP------VE-VARWRIRSDIRRQVLFTRLNLVED-EYPAVMNGTSMQDVILCRNVLIYFDDETIRAVVRRLYRA-LTPGGW
Q2W5V8      TVLGTDINQKFLSRAREGRYGDEWAFRT-MSDSLRAACFEK---V--GN---QWQIRPEFKRNVSFQYHNLIKS-PFPSLADNIAGFDIIICRNVIIYFSQATVESLVPCFRES-LNDGGW
D3NZL7      HIVGTDINDAFLEQARRGAYGDWAVRG-LAPETLDACFDR---Q--DR---LWSVKPKYRQWTSFMPFNLVEG-AIPSYPHGIGHFDIILCRNVMIYFDEATRHRLLGDLHKA-LADGGW
C7C9P3      TILGTDLSAGALAVAREAEFGPWALRT-LDTEERARWFRRTPARPGLP-HGGYALRPAFRRMVRFERQNLLT--LIDGSDRTQGGFDLILCRNVLIYFSTDHVTRIVRALGER-LNPQGW
B7KRG4      TILGTDLSAGALAVAREAEFGPWALRT-LDTEERARWFRRTPARPGLP-HGGYALRPAFRRMVRFERQNLLT--LIDGSDRTQGGFDLILCRNVLIYFSTDHVTRIVRALGER-LNPQGW
A9W6M0      TILGTDLSAAALAVAREAEFGPWALRT-LDTEERARWFRRTPARPGLP-HGGYALRPAFRRMVRFERQNLLT--LIDGSDRTQGGFDLILCRNVLIYFSTDHVTRIVRALGER-LNPQGW
C5AVV0      TILGTDLSAGALAVAREAEFGPWALRT-LDTEERARWFRRTPARPGLP-HGGYALRPAFRRMVRFERQNLLT--LIDGSDRTQGGFDLILCRNVLIYFSTDHVTRIVRALGER-LNPQGW
B1ZEP6      TILGTDLSAAALATARAAEFGPWALRT-LDTEERARWFRRTPARPGLP-HGGYALRPAFRRMVRFERQNLLG--LIDGSDRIQGSFDLILCRNVLIYFSPDHVNRIVRALGER-LDARGW
B1LWP1      SILGTDISVEALATARAAEYGRWALRT-MPPEDRLRYFTRLPAAPGIRREGGYALRPEYRGSVRFERGNLLN--LVEPGPPQGEPFDLILCRNVLIYFDARTVAAVVRGLGRR-LRPEGW
B7JYH8      FILGVDLSQDALEIAKTACYSSWSFRK-VDYSIQDKYFNKI--------GEQYYLNDEIKKMLTFKQCNLVQD-PFWESSYGLSNMDLIICRNVFIYFSQPTIAKILDKFYYC-LQPLGY
E0UB22      KILGIDINQDAITQARKGVYNAWSFRQ-VDEEIKEKYFNFT--------NEQYELNQSIKSLVKFERINLAKD-IFPQMDTDLRDMDLIICRNVFIYFATYSAIVNVIEKFYNT-LQIGGY
C6DZA2      QIIGTDINSKVIDQAGKGIYTEWSFRQ-TEQRLRDKFFSKR--------EGCWEIDPDIKAMVTYRCCNLVLD-PFPDLSGELNNMDLIVCRNVFIYFHPEAVGQVVTKFADT-LTEGGF
B0TG84      DVIGTDINEASLHIARAGRYSEWSLRA-TSPERRQEHFRRVDPGRGRRAGPVWEVDPAIRSLVQFYRLNLLDG-PLPDRFAPVASTDLILCRNVLIYFRPEAIRQVLTDFFRC-LNPGGF
A5G943      KILATDIDNRALKRARDGIYGPWAMRV-IEKRYLDRYFDK--------IGKGYRLKDEVKSLVDFSHLNLQTA-EFPSSAGEFSELDAVFCRNVIIYFTLATTREIIEKFSAC-LKPAGY
B9LZW0      SILATDIDNRALKRAQEGIYSPWAMRV-MEKRNLDRYFEK--------SGKKFRIRDEVKSLVRFAHHNLTSS-TPDQEEQDRERFDAVFCRNVTIYFTIDITRQIIEKISNC-LKPGGY
B5EF82      RIIATDINNRSLKLAREGVYSSWSLRI-TQGEQIGRYFDR--------VGQSFLIKDEVKRLVHFCHLNLSGP-GRDEMWQDLSALDAIFCRNVLIYFTPQAADQVLRCLAGA-LKVSGQ
C6E726      RIVATDINNRSLKLAREGVYSPWSLRI-TQGEQIGRYFDR--------VGQSFLIKDEVKRLVHFRHLNLSGP-GHDEMWHELSELDAIFCRNVLIYFTPQAADEVLRRLADA-LKISGQ
A9WBT1      SILATDLSASALAKARQGVYRGRTISN-LTPAQRARFFTT--------TDAGLSIHERVRQLVTFVQHNLLEP-FPAAAYG----THILFCQNVTIYFSLDTCRSLMARFYDA-LADGGI
B9LEA3      SILATDLSASALAKARQGVYRGRTISN-LTPAQRARFFTT--------TDAGLSIHERVRQLVTFVQHNLLEP-FPAAAYG----THILFCQNVTIYFSLDTCRSLMARFYDA-LADGGI
B8G3A2      EILATDLSATAIEKARAGVYRGRTLTN-LTPLQRLRFFTP--------VGNALTIHERVRQLVTFMQHNLLDP-FPPAVRG----THILFCQNVTIYFSLDTCRTLMARFYDI-LADGGT
A7NRP0      EIWATDLSELALEKARTGFYRGRSLNN-VTPMLLNRYFVR--------HGDGFLVSDAVRALVRFEQLNLLET-FPPTAYG----VDAIFCQNVTIYFRPETRRSLIERFHRC-LPVHGL
A5UPC3      EIWATDLSDLALEKARAGFYRGRSLNN-VAPSLLSRYFVK--------RGDGFLVADAVRALVHFEQLNLLEP-FPPTAYR----VDAIFCQNVTIYFQPETRRSLIERFYRC-LPSHGL
B7K4M9      EIDGMDISETALAKAKQGIYGSNSFRT-HDFPEDKSFFTIGN--------DLYEVPPSVRNQVKFRQNNLLNT-WL--LQE---PYQIIFCRHLLIYLDNASRNRAINILEKI-LCDRGL
E0UAE6      QIDALDISHRALEKAKKAVYGENSFRE-DLTLPKNHYFHQIQ--------ESYQLSELVRETVQFKEGNLLGP-HLP-VHK---PYQIIFCRHLLIYLDVSARSRAIDRLDQL-LVPRGF
B2J4R2      AIDAIDISHRSLAKAKRGVYTKNSFRG-DAWTERERYFQQTA--------EGYELCQSVRELVNFQQGNVMTS-LAL-TQK---QYDIIFCRNLLIYLQSEACSQVLAAIDRL-LRPGGL
B1Z0W8      MIDALDLSVRAIEQARLGCYGRNAFRG-TATEFRARYFTPAA--------DGWLLDERVRARVQFRQANLIEP-GVDTG-I---RYDFVFCRNVLIYFDRDAQDRVIGSLESW-LADDGM
Q0B461      MIDALDLSVRAIEQARLGCYGRNAFRG-TATEFRARYFTPAA--------DGWLLDERVRARVQFRQANLIEP-GVDTG-I---RYDFVFCRNVLIYFDRDAQDRVIGSLDSW-LADDGM
Q1BLP9      TIDAIDLSARAIEQARLGCYGRNAFRG-TATEFRTRYFTPTR--------DGWLLDERVRACVQFSHANLVEP-VADTG-I---RYDFVFCRNVLIYFDRDAQDRVIRSLDAR-LADDGM
A0AYP9      TIDAIDLSARAIEQARLGCYGRNAFRG-TATEFRTRYFTPTR--------DGWLLDERVRACVQFSHANLVEP-VADTG-I---RYDFVFCRNVLIYFDRDAQDRVIRSLDAR-LADDGM
B1K4W3      TIDAIDLSARAIEQARLGCYGRNAFRG-TATEFRTRYFTPTR--------DGWLLDERVRACVQFSHANLVEP-VADTG-I---RYDFVFCRNVLIYFDRDAQDRVIRSLDAR-LADDGM
A9AP36      AIDAIDLSARAIEHARVGSYGRNAFRG-TSTEFRARYFTPVA--------DGWLLDERVRACVQFRQANLLEP-LSDTG-I---RYDFVFCRNVLIYFDREVQDRVIRSLDAC-MTEDGM
A4JLK2      AIDALDLSARAIEHARAGCYGRNAFRG-TQTAFRTRFFTPTA--------DGWLLDEQVRSRVQFRQANLMEA-CADTG-V---RYDFVFCRNVLIYFDRDAQDRAIRQLASR-LADDGM
A1UYE3      EIDALDISARAIAHAQRGRYGRNSFRG-HVLGFRDLHFKAAA--------DGWVLDERVRACVRFRQANLLDL-LGCAG-E---PYDFVFCRNVLIYFDRDAQDRVVRIVEEQ-LDARGI
A2S0C7      EIDALDISARAIAHAQRGRYGRNSFRG-HVLGFRDLHFKAAA--------DGWVLDERVRACVRFRQANLLDL-LGCAG-E---PYDFVFCRNVLIYFDRDAQDRVVRIVEEQ-LDARGI
A3MB25      EIDALDISARAIAHAQRGRYGRNSFRG-HVLGFRDLHFKAAA--------DGWVLDERVRACVRFRQANLLDL-LGCAG-E---PYDFVFCRNVLIYFDRDAQDRVVRIVEEQ-LDARGI
Q3JJX9      EIDALDISARAIAHAQRGRYGRNSFRG-HVLGFRDLHFKAAA--------DGWVLDERVRACVRFRQANLLDL-LGCAG-E---PYDFVFCRNVLIYFDRDAQDRVVRIVEEQ-LDARGI
A3P8B0      EIDALDISARAIAHAQRGRYGRNSFRG-HVLGFRDLHFKAAA--------DGWVLDERVRACVRFRQANLLDL-LGCAG-E---PYDFVFCRNVLIYFDRDAQDRVVRIVEEQ-LDARGI
A3NMV6      EIDALDISARAIAHAQRGRYGRNSFRG-HVLGFRDLHFKAAA--------DGWVLDERVRACVRFRQANLLDL-LGCAG-E---PYDFVFCRNVLIYFDRDAQDRVVRIVEEQ-LDARGI
Q2T7Z6      EIDALDISARAIAHAQRARYGRNSFRG-HALGFRDRHFKAAA--------DGWVLDERVRACVRFRQENLLDL-LGRAG-E---PYDFVFCRNVLIYFDRDAQDRVVRIVEER-LDERGI
B2JNG9      TVEAFDISARVIDHARAGRYGRNSFRG-LPLAFRDRHFTALE--------NGWQLDERVRQTVRFAQANLFDL-QANAQ-A---PYDFIFCRNVLIYFDREAQDRAIRLLDAS-LADGGT
B2T7T3      RIDAMDISDRSLAVAQRAVYGRNSFRG-NAFAFRDAHFTRTE--------DGWRLAPRIVEAVRFSRANLMQL-DASSL-G---VYDFVFCRNVLIYFDRDAQQKALQALTSV-LAGNGT
D8J0F2      VIDAYDIRTRSLEIAAAGLYGRNSFRG-QELAFRDRHFDLEQ--------GQWRLHERIRSQVRFAPGNLLAP-DFLRHSQ---PYDFIFCRNVLIYFERDVQRQVVQLLASV-MKPDAL
A6STX7      HIDAIDICHRSLAIAERGVYGRNSFRG-KLLDYRDRHFDTSK--------DEARLSELVRQQVNFRHGNMFAG-DFLLNEK---PYDFVFCRNVLIYFDREMQQQAVEVLDRL-LHPQGT
Q0JZJ6      AIDAVDISARALARARQGEYGANAFRS-APLDFRDRYFTATP--------SGYVLDARVRAQVRLLQGNLVDP-GLLAGER---PYDFVFCRNLLIYFDAPGQRHAVQTLARL-AAADGI
B3RBS6      YIDAVDISARALARARQGQYGANAFRS-APLDFRDRYFTATP--------TGYVLDSRVRAQVRLLQGNLVEP-GLLSGEA---PYDFVFCRNLLIYFDAAGQRQAVQTLVRL-TMADGL
Q46V25      RIDALDISARALARARAGIFGSNAFRG-APLDFRERYFTPCP--------GGYALDARVRELVQLRLGNIVDA-NLLAGEA---PYDFVFCRNVLIYFDADVQRRAVQTLARL-TASDGM
Q1LG87      RIDAIDISARALERARAAVYGDNAFRS-QPLDFRDRYFMSVTG-----TQATYQLLPKVRNQVRLLCGNLVDP-DLLRHEA---PYHFAFCRNLMIYFDPATQCEAIRTLRRL-TRPDGM
Q2KV72      RIDGVDVSERALAVAQAGRYGRNAFRG-GEQGFRLRYFDAHG-----------QLSRAVMNCVRFMQGNLFDP-VLQAGLP---GYDVLFCRNLLIYFDSTTQARALATMTRM-VSTDGA
B7V0Y7      EVDALDVSARVIERASLGVYGRNSFRG-DELGFRDRHFSEVA--------EGYQLAEQVRRKVRFRCGNLLDP-GLLAGEV---PYDFVFCRNLLIYFDRPTQSEVVEVLKRL-LRSDGA
Q02RH8      EVDALDVSARVIERASLGVYGRNSFRG-DELGFRDRHFSEVA--------DGYQLAEQVRRKVRFRCGNLLDP-GLLAGEA---PYDFVFCRNLLIYFDRPTQSEVVEVLKRL-LRPDGA
A6V180      EVDALDVSARVIERARLGVYGRNSFRG-DQLGFRDRHFSEVA--------DGYQLAEQVRRKVRFRCGNLLEP-GLLAGEA---PYDFVFCRNLLIYFDRPTQSEVVEVLKRL-LRPDGA
Q88MS8      LVDGMDISPSSVAKAGQAVYGRNAFRG-SELGFRERYFDALD--------EGHRLHERVRQQVSLRVGNVLDP-ALASRDG---LYDFVFCRNLLIYFDVPTQQRVFEVLKRL-LHPQGV
A5W896      LVDGMDISPSSVAKAVQAVYGRNAFRG-SELGFRERYFDALD--------EGHRLHERVRQQVSLRVGNVLDP-ALASRDG---LYDFVFCRNLLIYFDVPTQQRVFEVLKRL-LHPQGV
B0KRK9      LIDAMDISPSSVAKARLAVYGRNAFRG-SELGFRERYFDALD--------EGHRLHERVRQQVSLQVGNVLDP-ALASRDG---LYDFVFCRNLLIYFDVPTQQRVFEVLKRL-LHPQGV
Q1IDW8      RIDGMDISPNSVAKGLQGDYGRNSFRG-SDLAFRERHFSKFA--------ELHRINERVRQQVNLQVGNVLDP-ALKSRHG---LYDFVFCRNLLIYFDVPTQQRVFEVLKHL-THGQGV
Q98PC8      KIDGVDVSSRNIATAERAIYGRNSFRG-SHLEFKDRYFAAEG--------GLRPIAGVLKQVRFRVGNLFGS-RASFGQE---VYDILLCRNLLIYFSRELQDRALICLKDL-LAKDGL
D8FCI6      SIAGIDISEKALERARDGVYGQASFRG-KDLSFRDHYFRRKG--------DVYRLLDPVRGKVFFKTGNVMER-HFATG-L---TYDVIFCRNLMIYMSPAARNQTLKTMSRL-LDDKGI
C6BU38      RVDGVDISERALHKARGAVYGDNSFRS-ELPIYAQSWFRKCE--------QGRMLAPEIKDSVNFHAGNIIDG-CLPNG-----RYDVIFCRNLLIYLDDGSRKCLAALLNEK-LKDDGL
Q60AM0      RVEALDISARAIEAARAGIYGGRSFRSPGDGTWRSRYFDELGD-------GRYRVKDGLRGDVEYRVAHLGDP-GWGGG-R---RYQAVFCRNVLIYLRAELRARIMDQCRDA-LDPGGL
A0L7P7      RLQGCDIDTTVLQRARQGQYGSFSFRL-LDPQLKARYFEPLPE-------QQFQIKPVIAQQVNFSLLNLMDA-NYPSMLH---GQDIIFFRNVSIYFDQQHRREIQSKLRDL-LNFGGA
Q2W2W8      RILGGDIDHHALARARAGRYTAFSFRS-LAPELRRRYFQPAGR-------DAMVVDERVKAMVSFHHLNLLAE-TFPPALG---ALDVVFFRNVSIYFDVLTRRTIQQAFRRV-MTPRGH
Q1NKB1      RIVAADIDGQAIAAARRGIYGDGAFRG-PSESWRHRYFQPQPK-------GGWQLAREIRQRVEFEVRNLLTP-GSPLLLE---PPEIILYRNVSIYFPPPVQREIFSQLATR-LAPGGC
Q1NWT4      RIVAADIDGQAIAAARRGIYGDGAFRG-PSESWRHRYFQPQPK-------GGWQLAREIRQRVEFEVRNLLTP-GSPLLLE---PPEIILYRNVSIYFPPPVQREIFSQLATR-LAPGGC
Q1NLW5      RIVAADIDGQAIAAARRGIYGDGAFRG-PSESWRHRYFQPQPK-------GGWQLAREIRQRVEFEVRNLLTP-GSPLLLE---PPEIILYRNVSIYFPPPVQREIFSQLATR-LAPGGC
Q1D4W0      DLWATDLNLAAVEAAKQGRFTSRRAIS-INQARLTRFFKP--------VEEGYEALPALREYIRFDGQNLAVP-VFDKVA--LSSLDLILCRNVIIYFDLPTIRGLMDRFLAA-LRPGGL
E3FYQ2      DLWATDLNLAAVEAARMGRFSVRRSSG-ISSERLERFFHP--------VEDGMEVQPTLREYVRFEGQNLAAP-AFDAVT--PGSLDLILCRNVIIYFDLPTIRALMDRFLVA-LRPGGL
A7H826      ELLATDVNPEAVAHAARGAYDARRARE-IPAPLRERHFDRD-------PHDRYHVRASLRRMIAAIRPHNLVS-GVMRPP--EGGWDVIFCRNVIIYFDTPTTQRVLTQFHHA-LAPGGY
Q1D226      TVLGTDIAGDTLRRARWLAYGREQLRR-VPPELRSRYFVR--------SGAKEALTPALRERASFQCHNLMES-PYPVAPG-GGGFDVIFCRNVLIYFTAEAFQRTVEALAGS-LAPGGA
Q08NE7      SVLGTDISEQALLRARTLTFHPEQVRR-LPASVRDRYFLS--------EGARSFLAPELRGRALFQLHNLMEA-PYPAH---GEGFDIIFCRNVLIYFTTEAFDRVVEALAER-LAPGGT
Q1D8X4      RILATDVSGRALQRAREGVYGPWSLRR-IEPEQEKRFLVANG--------DDYSVIPQVRHAVEFRRHNLAVD-PPP-----FMGLGAIFCRNVLIYFPTELAQEVLKRFISA-LAPGGL
Q08UZ6      HVLATDISARSLSRARVGTYSPWSVRR-VEPAMEKRFLTAHP--------GGMSVCTQARQPVEFRRHNLVSD-LAP-----VSNQDAVFCRNVLIYFPPELVRQVLTKLVGA-LAPGAL
A7H7I5      RILATDVSRALAVAAEATYGPWSLRR-LDPVARARHLAGEM--------PVVRVREEVRAAVELRRHNLFSE-PAP-----PGPFDLVVCRNVLIYFDPETARAIAARLLEA-VAPGGF
Q1CX10      AVHATDVSRGALARARKAHYGDWSLRG-GWADRMRAHLRAEG--------RRYVLSPEVRKRVRFSYLNLALD-TWPSADSGIWKLDVIFCRNVLIYFNRPTIEAVARRLHDA-LDEGGY
E3FZL3      EVHATDISRAALAHAQQASYSAWSLRS-TGAERMRPFLRMED--------KRYVLASEVRHRVRFHYLNLALD-TWPSPESGISGMDIIFCRNVLIYFTRPTIAAVARRLYES-LADGGF
A7HCQ0      HLVGTDLSEKALARARRGEYSLWSLRG-AADGRARRHLTERD--------GRFLVAPEIAARVRFFPLNLARP-EYPSAARGLSDLDLVLCRNVLMYLDRAHVAEAAGRLFET-LTPGGW
E3FRV5      SITGVDVNPRAIERARKARYAAWSLRS-CPEALRERWFHALP--------NGDFELRPSARERVLFEERNLLEE-DPAFWASG--SFDVILCRNVTLYFTPEVTRTVMARLERA-LTPGGA
Q08UV4      SITGVDVNPRAIERARKARYAAWSLRS-CPEALRERWFHALP--------NGDFELRPSARERVLFEERNLLEE-DPAFWASG--SFDVILCRNVTLYFTPEVTRTVMARLERA-LTPGGA
A7HCX6      RVLGIDVNPAAIARARSGRYSPWSLRQ-TTPALRARFFRSE--------GRDARVVDDVRALVSFEERNLVEE-DPELWAPG--SFDVVFCRNVVMYFAPEVFRRVVARIAGA-LVPGGY
C6C1V4      EIFGTDIDSKALIKAREGCYRKWSFRS-EDTGFRDIFFRKVG--------SNSFLLDSSIKRMVNLSRLNLMDA-SVPVALQ---DMDVILCRNVMIYFSSAGVNSVLDKLWDC-LTPGGW
D2Z5P5      RVYGTDLNESSLKKARQGMYGRWSFRG-MSDIEISRYFDIVG--------DDRFKVKDRYRSRVSFESANLVSS-SPLRRED---KMDVIFCRNVMMYFDEANRKKVLHSFRKA-LSPDGW
D0LTR4      EILGTDIDRERLATAREGVYRPWSLRN-MPPQVRERWFAETEAG------GYALCQPARALVRFEHLNLIDS-DDDEARWP-HDVDVAFCRNVLVYFGSAAIARTAAGLRDA-LRLEGW
Q08XU2      EVLGTDLNEASLAHARRATYGTWSRRD-SGPHLFPLYRPTGE--------REVVILEGVRNVTSFAVTNLMG--PLPDLFG---HFEFILCRNVLTYFTPAAREVAIGHIVRA-LAPGGY
A0L8P6      HALGTDINKEALARAQQGRYGDWAMRG-LPDALKQSFFSQDG--------NYWQLQTHRFRAQIKFDYLNLIEG-GYPSILQGTGQQDLILCRNVFIYFDNPTVDAIVARFSRC-LNPGGL
D0LUD0      RILATDLNRALRHARDARYSRRAIAR-LPDAWQTRYFDYLD--------DETARVIEALRERVSFARHNLRSDETLPPGWR---ELDAVVCRNVLIYFQRYEAVEMVHKLVAH-CRVGGY
D3PC59      SIIGTDLSSHAIEIARRGIYSKWHVRN-LDKSYLSKYFILDD--------NKFILKEDIRDLVRFKKHNLLKDDYFLN-------CDIVFCRNVLIYFNEQNIKYVVERFIDESLSENGY
D3P9S2      KIVGIDINQNQIEYAKKGIYQKWYLRN-TPNDIIEKYFIKLD--------ENNYKLKDIVKNSVTLLHKNLFEFDDA--------EYDIVYCRNILMYFDKKDTDKTIKKIDEL-TNEYGT
```

```
                    490       500       510       520       530       540       550       560       570       580       590       600
                      |         |         |         |         |         |         |         |         |         |         |         |
E. coli      LFAGHSENFS----HLERRFTLR--GQTVYAL----------------------------------------------------------------------------------------
S. typhi     LFAGHSENFS----NLVREFSLR--GQTVYAL----------------------------------------------------------------------------------------
Q12AR4       LVMSPSEDSS----SLFPNFVPVNFPSAILYQK---------------------------------------------------------------------------------------
Q2LR61       VLPSVTEGFH----LLFTRFVAVTFPGVTLYQKRMDQRGTLE------------------------------I-----------------------------------------------
Q2YC83       LIVSPVEMSR----ASFPQFRLHTVSGTGLYQK---------------------------------------------------------------------------------------
B8FNR6       FFVGPSEAAF----VEHPNLAHIRYPGAIIFQKQAAR-----------------------------------------------------------------------------------
B5ECK8       LFLGPTEVDH----QALEGFTCLHCDGTLVLQK---------------------------------------------------------------------------------------
C6DYF7       LFLGPTEVDH----QALEGFTCLHCDGTLVLQK---------------------------------------------------------------------------------------
A9WGY5       LVVGHAEPNV----VLYRQFETHNAPGTILYRK---------------------------------------------------------------------------------------
B9LL63       LVVGHAEPNV----VLYRQFETHNAPGTILYRK---------------------------------------------------------------------------------------
A7NNV5       LIVGHAEARS----DFFAQFEVVNCPGTVIYRK---------------------------------------------------------------------------------------
A5URZ9       LIVGHAEARS----DFFARFEVINCPGTVIYRK---------------------------------------------------------------------------------------
Q2W5V8       LIMGHAEPNQ----RLFADFRTVNTPGAVLYQR---------------------------------------------------------------------------------------
D3NZL7       LVVGHAEAGP----QMNELFIPVSVPGATLYRK---------------------------------------------------------------------------------------
C7C9P3       LLIGHAEPNP----TFSQWLQPVALPGTVAYRP---------------------------------------------------------------------------------------
B7KRG4       LLIGHAEPNP----TFSQWLQPVALPGTVAYRP---------------------------------------------------------------------------------------
A9W6M0       LLIGHAEPNP----TFSQWLQPVALPGTVAYRP---------------------------------------------------------------------------------------
C5AVV0       LLIGHAEPNP----TFSQWLQPVALPGTVAYRP---------------------------------------------------------------------------------------
B1ZEP6       LLIGHAEPNP----TFSQWLQPVALPGTVAYRP---------------------------------------------------------------------------------------
B1LWP1       LLLGHAEPSP----AFASFLDAVSLPGTVAYRPRADAPP---------------------------------------------------------------------------------
B7JYH8       LMTGHAEITSNN--DTIKKFQSKLFPESVIYQKVKNKVGSEL--------------------------------SIN-------------------------------------------
E0UB22       LLTGHAELSP----EQVRGFQVHLFPESVIYQKREKWVS---------------------------------------------------------------------------------
C6DZA2       LLTGHGELHL----QALYRLKSRMIDEQMILQKGTDLTS---------------------------------------------------------------------------------
B0TG84       LLTGHGELLGLEPGQALFSFEHLLFPEGHIWQKPVIPPP---------------------------------------------------------------------------------
A5G943       LFLGHSETLS----HISSRFERQTQDGGFYYLKKVFPPVA------------------------------------------------------------------------------VS
B9LZW0       LFLGHAETMA----QISSRFERQSHDGSFYYRKKTAKNGI------------------------------------------------------------------------------PT
B5EF82       LFLGHAETLL----QQDSGLEIRRQGKTFFYLKTAPRTSQ------------------------------------------------------------------------------TP
C6E726       LFLGHAETLL----QQDSELEIRRQGKTFFYLKSGPRTPQ------------------------------------------------------------------------------TP
A9WBT1       LCLGFSETLW----NIFDRLTPVAVDGAFLYRKDPPPAR---------------------------------------------------------------------------------
B9LEA3       LCLGFSETLW----NIFDRLTPVAVDGAFLYRKDPPPAR---------------------------------------------------------------------------------
B8G3A2       LCLGFSETLW----NIFDRLRPVSVDGAFLYRKDPPRQR---------------------------------------------------------------------------------
A7NRP0       LFLGFSETLW----NVFDGFRSREVSGAYVYQKVNPPDRP------------------------------------------------------------------------------TQ
A5UPC3       LFLGFSETLW----NVFDGFRSREVSGAYVYQKVERPPSP------------------------------------------------------------------------------LQ
B7K4M9       LFVGAVETTL----LNSPPFRYMPHPSAFAYQK---------------------------------------------------------------------------------------
E0UAE6       LFVGSPEIAL----LPSPRFQVIDYPYAFVYQKNE----------------------------------------------------------------------------------K-R
B2J4R2       LFVGASETAK----IVADQYTSTRKPFTFAYRK---------------------------------------------------------------------------------------
B1Z0W8       LFVGPAETGV----AMRHGMRSARVPLAFAFHR---------------------DHGGAAV-----------------------------------------------------AADASA
Q0B461       LFVGPAETGV----AMRHGLRSARVPLAFAFHR---------------------DGGGAAVGA---------------------------------------------------AADASA
Q1BLP9       LFVGPAETGV----AMRHGMRSARVPLAFAFHR---------------------EPAGAAA-------------------------------------------------------DACA
A0AYP9       LFVGPAETGV----AMRHGMRSARVPLAFAFHR---------------------EPAGAAA-------------------------------------------------------DACA
B1K4W3       LFVGPAETGV----AMRHGMRSARVPLAFAFHR---------------------EPAGAAA-------------------------------------------------------DACA
A9AP36       LFVGPAETGV----AMRHGMRSARIPLAFAFHR---------------------ERSGAAA-------------------------------------------------------DGCA
A4JLK2       LFVGPAETGV----AMRCGMRSARVPLAFAFQR---------------------ADDGAAA-------------------------------------------------------DECV
A1UYE3       LFVGPAETGV----VMRQGLASAQIPLAFAFRKPAVEAAARPGAGGVGARRGGAAPGGAANVVNAANAANAGAAAGDRVPTFGGTLRAIAHEWFFDAGWPTPEPIVRAIDGARSAFESFA
A2S0C7       LFVGPAETGV----VMRQGLASAQIPLAFAFRKPAVEAAARPGAGGVGARRGGAAPGGAANVVNAANAANAGAAAGDRVPTFGGTLRAIAHEWFFDAGWPTPEPIVRAIDGARSAFESFA
A3MB25       LFVGPAETGV----VMRQGLASAQIPLAFAFRKPAVEAAARPGAGGVGARRGGAAPGGAANVVNAANAANAGAAAGDRVPTFGGTLRAIAHEWFFDAGWPTPEPIVRAIDGARSAFESFA
Q3JJX9       LFVGPAETGV----VMRQGLASAQIPLAFAFRKPAVEAAARPGAGGVGARRGGAAPGGAANVVNAANAANAGAAAGDRVPTFGGTLRAIAHEWFFDAGWPTPEPIVRAIDGARSAFESFA
A3P8B0       LFVGPAETGV----VMRQGLASAQIPLAFAFRKPAVEAAARPGAGGVGARRGGAAPGGAANVVNAANAANAGAAAGDRVPTFGGTLRAIAHEWFFDAGWPTPEPIVRAIDGARSAFESFA
A3NMV6       LFVGPAETGV----VMRQGLASAQIPLAFAFRKPAVEAAARPGAGGVGARRGGAAPGGAANVVNAANAANAGAAAGDRVPTFGGTLRAIAHEWFFDAGWPTPEPIVRAIDGAPSAFESFA
Q2T7Z6       LFVGPAETGV----VMRQGLASAQIPLAFAFRKPAADAATRPGASGIGAQAAGGAWRGAAAGV-AASAGGAGSAGDDRTPAIGGTLRAIAHEWFFDAGWPTPEPIARAIDGAPHAFESFA
B2JNG9       IFVGPAETGL----MLRHAMTSARIPLAFAFRR------------------------STRE-------------------------------------------------------ERDT
B2T7T3       LFVGPAETGL----LMRYGMQSAKIPLAFAFHH------------------------GAPEET---------------------------------------------------QWNSWH
D8J0F2       IFVGPAEGGI----LLGPTMESAGIALAFGFRK------------------------------------------------------------------------------------RVA
A6STX7       IFVGPAEAGV----MLRPNLVSAGIPLAFAFRK------------------------------------------------------------------------------------RNG
Q0JZJ6       LFVGPAEASV----LSAQGLEPVGLPLTFAFRKPS----------------------------------------------------------------------------------AVP
B3RBS6       LFVGPAEASV----LSAQGLEPVGIPLSFAFRKPAA---------------------------------------------------------------------------------AAP
Q46V25       IFVGPAEASL----LSAQGLPSAGVPLAFAFRKAT-------------------------------------------------------------------------------------
Q1LG87       IFVGPAEASL----LTREGLAGAGVPLGFAFHAQP-------------------------------------------------------------------------------------
Q2KV72       IFVGPAEASL----LTARGYPALAGAGAFAFSARP-------------------------------------------------------------------------------------
B7V0Y7       MFIGPAEASL----LSQHGMQPIGVPLSFVFRRTS------------------------------------------------------------------------------------E
Q02RH8       MFIGPAEASL----LSQHGMQPIGVPLSFVFRRTS------------------------------------------------------------------------------------E
A6V180       MFIGPAEASL----LSQHGMQAIGVPLSFVFRRAA------------------------------------------------------------------------------------E
Q88MS8       LFIGPAEGSL----LARMGMRPLGIAQSFAYVRHE------------------------------------------------------------------------------------G
A5W896       LFIGPAEGSL----LARMGMRPLGIAQSFAYVRHD------------------------------------------------------------------------------------G
B0KRK9       LFIGPAEGSL----LARLGMRPLGIPQSFAYVRQE------------------------------------------------------------------------------------G
Q1IDW8       LFIGPAEGSL----LARLGMRPLGIAQSFAYVRQE------------------------------------------------------------------------------------P
Q98PC8       LLVGPAETAL----PRLHGFIPADWPLAFAFLKSE-------------------------------------------------------------------------------------
D8FCI6       FFCGHAERQT----ALDRGFEAINETGVFACRKIR----------------------------------------------------------------------------------R-E
C6BU38       LFVGHAEILP----VFNDWFTPVRKQGTFALRKGK---------------------------------------------------------------------------------RKVA
Q60AM0       LVLGHADGIG----GPDRGFRRHGVAGAFSWIR-R-------------------------------------------------------------------------------------
A0L7P7       LVAGSAETLP----NDFGLLTLEQWGGCFFFRKGKS----------------------------------------------------------------------------QAAEEAVE
Q2W2W8       LVIGSAETLA----NDLGVFRLVEDEGAFYFVQG------------------------------------------------------------------------------DRAGPPVP
Q1NKB1       LLVGASETLH----HDLAILSLEERDNLYFFRKPK----------------------------------------------------------------------------------LVP
Q1NWT4       LLVGASETLH----HDLAILSLEERDNLYFFRKPK----------------------------------------------------------------------------------LVP
Q1NLW5       LLVGASETLH----HDLAILSLEERDNLYFFRKPK----------------------------------------------------------------------------------LVP
Q1D4W0       LFLGYSESLF----KVYDRFEMIEVDGAFVYRRPLNDKSM-----------------------------------------------------------------------------RAP
E3FYQ2       LFLGYSESLF----KVYDRFEMIEVDGAFVYRRPLLG-----------------------------------------------------------------------------------VA
A7H826       LFLGYSESLF----RIFDGFELTEVAGAFLYRRPETVR----------------------------------------------------------------------------------
Q1D226       LVLSASEPLL----RVPPTLRVRRGEQAFFYVRPEEG-----------------------------------------------------------------------------------
Q08NE7       LVLSAAEPLL----HAPPSLRIIRCEHAFFYVRTFD------------------------------------------------------------------------------------
Q1D8X4       LFVSPAEVPL----TNGLGLETVDAEGSVVLRVPVPGA----------------------------------------------------------------------------------
Q08UZ6       LFLSPAEVPL----ANGLGLEVLDVEGTPVLRRPVT------------------------------------------------------------------------------------
A7H7I5       LLLGPVEVPL----AERLEAERIEVGGTTLLRRMAEG-----------------------------------------------------------------------------------
Q1CX10       LFTGPSDPPL----GGLAPLESILTEWGVLYRRPLPGATLSL------------------------------------------------------------------------------
E3FZL3       LITGPSDPTL----AGLAPLEPLLTGWGVAWQRLPGA-----------------------------------------------------------------------------------
A7HCQ0       LLTGAADPPL----GALTGFTVRAGPFGVAYQRPVAPS----------------------------------------------------------------------------------
E3FRV5       LLLGPSETPR----GFSESFEVLQVGEAFYHR----------------------------------------------------------------------------------------
Q08UV4       LLLGPSETPR----GFSESFEVLQVGEAFYHR----------------------------------------------------------------------------------------
A7HCX6       LFLGHAETLR----GVSTEFHLRQSHGTFYYQSRGEEEAG-------------------------------------------------------------------------------E
C6C1V4       LVATPSESGL---LSSHGRFEPVNVGGVLLYRKNENYVPECRMDFDG-------------------------------------------------------------------------
D2Z5P5       LVVAACETPI----LDGSPFHPVRVGEQTFFTVE--------------------------------------------------------------------------------------
D0LTR4       LIPGPSDPLL-----ACAGLSIDASPGFIAYRRR--------------------------------------------------------------------------------------
Q08XU2       LFLGTVEVDR-----VPEGLVRVGPPELQAFHR---------------------------------------------------------------------------------------
A0L8P6       LITGACDYVP----NHIPGLVYRHEGGVFFFEKVEATGALN-------------------------------------------------------------------------------
D0LUD0       LLLSAVEQPL----FWMSELAPQRETDQLMQVSPGPVSVG--------------------------------------------------------------------------------
D3PC59       LFLAPGEFHF----LNQLGYKTIFIENNIVFKKGN-------------------------------------------------------------------------------------
D3P9S2       IIFGTSDILT----IPTNTFERYEINGVTVFRKKK-------------------------------------------------------------------------------------
```

```
                    610       620       630       640       650       660       670       680       690       700       710       720
                      |         |         |         |         |         |         |         |         |         |         |         |
E. coli      ------------------------------------------------------------------------------------------------------------------------
S. typhi     ------------------------------------------------------------------------------------------------------------------------
Q12AR4       -----------------------------------------SDVAPRTEPVTVTANGAPESG----------------------------------------------------------
Q2LR61       -----------------------------------------YHHSRRNSKNDAAVKGQTELGE---------------------------------------------------------
Q2YC83       ----------------------------------------------IDS-HDVHGNGDYFSV----------------------------------------------------------
B8FNR6       ------------------------------------------NKVQGADLVDTKAESAPVKRE---------------------------------------------------------
B5ECK8       ------------------------------------------------RPLPAKAQLSALSG----------------------------------------------------------
C6DYF7       ------------------------------------------------RSLPAKAHVAALSG----------------------------------------------------------
A9WGY5       --------------------------------------------PLQ---APLFT-NASTP----------------------------------------------------------V
B9LL63       --------------------------------------------PLQ---APLFT-NASTP----------------------------------------------------------V
A7NNV5       --------------------------------------------PLH---APLFD-EAIPS-----------------------------------------------------------
A5URZ9       --------------------------------------------PLN---APLFD-ETAVPP---------------------------------------------------------L
Q2W5V8       --------------------------------------------VDRPYVVAEPS-PPVPPP---------------------------------------------------------L
D3NZL7       --------------------------------------------QAERAIPAAG---PVPGA---------------------------------------------------------M
C7C9P3       --------------------------------------------IDTPVPPPPP---VLVPA---------------------------------------------------------L
B7KRG4       --------------------------------------------IDTPVPPPPP---VLVSA---------------------------------------------------------L
A9W6M0       --------------------------------------------IDTPVPPPPP---VLVPA---------------------------------------------------------L
C5AVV0       --------------------------------------------IDTPVPPPPP---VLVPA---------------------------------------------------------L
B1ZEP6       --------------------------------------------IDTPTTEPPP---VLVPA---------------------------------------------------------R
B1LWP1       -----------------------------------------VALPGTPVPDPAPS-TGQVPG---------------------------------------------------------P
B7JYH8       -----------------------------------------SSSEVEKITQNKCQVEKLQNQ---------------------------------------------------------L
E0UB22       -----------------------------------------QSSVSHPAPPQKTALEKLSTQ---------------------------------------------------------L
C6DZA2       -------------------------------------------AVSLPAVTP------LHHQ---------------------------------------------------------V
B0TG84       -------------------------------------------APSSRGIPPAAVTQPLPGA---------------------------------------------------------L
A5G943       N--------------------------------------------EQPRSKPSKT--TATVI------------------------------------------------------KPAR
B9LZW0       E--------------------------------------------PAPPRQPAPA--KA---------------------------------------------------------KSRQ
B5EF82       Q--------------------------------------------PPPCAKTAPQ--AIPELPEL--------------------------------------------------PAVPA
C6E726       Q--------------------------------------------PPPCVKPAPQ--AMPAA-----------------------------------------------------STVPA
A9WBT1       ----------------------------------------------------WMP--TTTGQR--------------------------------------------------------L
B9LEA3       ----------------------------------------------------WMP--TTTGQR--------------------------------------------------------L
B8G3A2       ----------------------------------------------------MVA--LSVPKPSR---------------------------------------------------PTLH
A7NRP0       H--------------------------------------------RSTSPRPSLP--TETRRRSPS-------------------------------------------------VVKVA
A5UPC3       R--------------------------------------------RTESRQPATT--AEAPQRLTP-------------------------------------------------VTATP
B7K4M9       ----------------------------------------------ILDPAPSS------------------------------------------------------------------
E0UAE6       A--------------------------------------------KHKNPKPEKS--II-------------------------------------------------------------
B2J4R2       ----------------------------------------------SEPPLPKLE--LLNI-----------------------------------------------------------
B1Z0W8       ARYAAPTRA------------------------------------VAPYRRAGRL--TVAP------------------------------------------PAAARPL-LAVVPPTWS
Q0B461       ARYAAPTPV------------------------------------VAPYRRAERL--TVAP------------------------------------------PAAARPL-LAVVPPTWS
Q1BLP9       ARPAAPLSA------------------------------------AAPYPGAERF--TVAP------------------------------------------LAAPRPV-LAVAPPAWL
A0AYP9       ARPAAPLSA------------------------------------AAPYPGAERF--TVAP------------------------------------------LAAPRPV-LAVAPPAWL
B1K4W3       ARPAAPLSA------------------------------------AAPYPGAERF--TVAP------------------------------------------LAAPRPV-LAVAPPAWL
A9AP36       TRRATPMPA------------------------------------AAPYRRAERF--APAP------------------------------------------SAWSGPAPLAFAAPVAP
A4JLK2       AGHVGGDGS------------------------------------YGRMARGGAL--GAGPTS-------------------------------------GAATASAMPLASAWACASVS
A1UYE3       ASPASAQGATRFAPSEPRAQAGDAGERPAGIFARAPGAAEPGGEALARTGDAGRF--ATAPRANAFEAGWTAAGAFS----------------------RAPDAGRARAGLFAPASDAAR
A2S0C7       ASPASAQGATRFAPSEPRAQAGDAGERPAGIFARAPGAAEPGGEALARTGDAGRF--ATAPRANAFEAGWTAAGAFS----------------------RAPDAGRARAGLFAPASDAAR
A3MB25       ASPASAQGATRFAPSEPRAQAGDAGERPAGIFARAPGAAEPGGEALARTGDAGRF--ATAPRANAFEAGWTAAGAFS----------------------RAPDAGRARAGLFAPASDAAR
Q3JJX9       ASPASAQGATRFAPSEPRAQAGDAGERPAGIFARAPGAAEPGGEALARTGDAGRF--ATAPRANAFETGWTAAGAFS----------------------RAPDAGRARAGLFAPASDAAR
A3P8B0       ASPASAQGATRFAPSEPRAQAGDAGERPAGIFARAPGAAEPGGEALARTGDAGRF--ATAPRANAFETGWTAAGAFS----------------------RAPDAGRARAGLFAPAPDAAR
A3NMV6       ASPASAQGATRFAPSEPRAQAGDAGERPAGIFARAPGAAEPGGEALARTGDVGRF--ATAPRANAFETGWTAAGAFS----------------------RAPDAGRVRAGLFAPAPDAAR
Q2T7Z6       APSVSPPGATRGASSEPRAQAGEAGQPAAGVFARAPGATEPGGEAFARARDAGGP--AAAPRANAFDSGWTAAGAFS----------------------RAPGAERARAGSFAPAIDGVP
B2JNG9       TRRAVQQAV------------------------------------PV----AARD--MHAP----------------------------------------------------------R
B2T7T3       TAPLATAAA------------------------------------LAMTHSQNPA--LSSP-----------------------------------------HVFSAEPFAWPDPVAPTP
D8J0F2       AA-----VP------------------------------------PLPPAWASLV--QAAP-----------------------------------------------P----------A
A6STX7       ---------------------------------------------PHPTAVARKI--IAP-----------------------------------------------------------A
Q0JZJ6       A-------------------------------------------ARAAPRAPVHA--AQPP------------------------------------------------R----------
B3RBS6       R-------------------------------------------ARAPSYPPVHT--TQPP------------------------------------------------R----------
Q46V25       P-------------------------------------------GEARSEQPLQA--SAAP-----------------------------------------------------------
Q1LG87       A-------------------------------------------SPSSHASAAHT--VMPP------------------------------------------------Q---------P
Q2KV72       --------------------------------------------APMPALRPRRA--PARS------------------------------------------------S----------
B7V0Y7       A-------------------------------------------PRGARPKAVSD--GARP------------------------------------------------V---------V
Q02RH8       A-------------------------------------------PRGARPKAVSD--GARP------------------------------------------------V---------V
A6V180       A-------------------------------------------PRGARPKPVAT--APRA------------------------------------------------A---------A
Q88MS8       D-------------------------------------------SAPLAAAPAQT--AKRA------------------------------------------------F---------T
A5W896       D-------------------------------------------SAPLAAAPAQP--AKRA------------------------------------------------F---------T
B0KRK9       D-------------------------------------------SAPMDVVPAQP--VKRT------------------------------------------------L---------A
Q1IDW8       A-------------------------------------------SSAAVSLPLPV--VR-------------------------------------------------------------
Q98PC8       ---------------------------------------------PVKTTAPKVS--AAIP-----------------------------------------------------------
D8FCI6       SG-------------------------------------------EKAAAWTAEK--VPVSG--------------------------------------------------------IH
C6BU38       TLLKQP---------------------------------------VCPHQKCPPN--SFSGS---------------------------------------------------------G
Q60AM0       ---------------------------------------------DAAEAEPPPA--RSSRP----------------------------------------------------------
A0L7P7       LWHGLSLS------------------------------------SPSHSWRPQDE--ALSWR---------------------------------------------------------G
Q2W2W8       ILP-----------------------------------------APHPAPVPPRP--APAPA---------------------------------------------------------R
Q1NKB1       APR-----------------------------------------HPRPTRRAGKK--GDTIP---------------------------------------------------------V
Q1NWT4       APR-----------------------------------------HPRPTRRAGKK--GDTIP---------------------------------------------------------V
Q1NLW5       APR-----------------------------------------HPRPTRRAGKK--GDTIP---------------------------------------------------------V
Q1D4W0       P--------------------------------------------LRITPYPGEP--DVAARRPVPADAFTADLRKRMLPEDVPLTTRLPAVSASSVAAPGSPSVTLPALGASSSPRSVV
E3FYQ2       R--------------------------------------------AKTTPVPMSP--PVSS--SVST--------P--VPLS--------------VAPAGTPPRPAPSFSRLRPPDAPV
A7H826       ------------------------------------------------TPLPAPR--PIRP--------------------------------------------------------VVP
Q1D226       ----------------------------------------------ESEAPPAPL--SWNG-----------------------------------------------------------
Q08NE7       ----------------------------------------------IHLAPPKRL--PASGN---------------------------------------------------------L
Q1D8X4       ---------------------------------------------PRAVTPESRL--PRLGMGRNQVASP-------------------------------------------------V
Q08UZ6       ---------------------------------------------PLGSVKEAPT--PIKPLARWKQE----------------------------------------------------
A7H7I5       ---------------------------------------------ERAGERVAPK--PARGRARSGSGASP---------------------------------------------TASR
Q1CX10       --------------------------------------------PVPAAWSPSST--GVPGTATGTTSLG--------------------------------------------------
E3FZL3       --------------------------------------------PRSAASAPPSF--PIRAPAPATPFTP--------------------------------------------------
A7HCQ0       --------------------------------------------SRTASVRPTS----ARRVAPRDAAAR--------------------------------------------------
E3FRV5       ---------------------------------------------RKTGLTPVPM--AAPRP----------------------------------------------------------
Q08UV4       ---------------------------------------------RKTGLTPVPM--AAPRP----------------------------------------------------------
A7HCX6       AAAV----------------------------------------PEREGPAPTPP--PDART----------------------------------------------------------
C6C1V4       --------------------------------------------FKFRDDFKTSFSSAAEQFDFN-------------------------------------------------------
D2Z5P5       ---------------------------------------------EEPSVPPERYLLDSAVS----------------------------------------------------------
D0LTR4       ---------------------------------------------TPAPPPPSPQRTTRPPP----------------------------------------------------------
Q08XU2       ---------------------------------------------PETPLMPAKAP----------------------------------------------------------------
A0L8P6       ------------------------------------------STSLITQSQPARPSQAALEPPK--------------------------------------------------------
D0LUD0       -------------------------------------------ASLHGALSPARIQEPPKLP----------------------------------------------------------
D3PC59       ---------------------------------------------YQDTQYSIKR-DRTKLP----------------------------------------------------------
D3P9S2       --------------------------------------------IKNELIKKTSENINRLEN----------------------------------------------------------
```

```
                 730       740       750       760       770       780       790       800       810       820       830       840
                   |         |         |         |         |         |         |         |         |         |         |         |
E. coli      ------------------------------------------------------------------------------------------------------------------------
S. typhi     ------------------------------------------------------------------------------------------------------------------------
Q12AR4       -------FPAIETSLSWEPSA-----------APVAVFES------------------------------------------------------------LQPKEDHAVPEAPGSAL
Q2LR61       -------WASLSTAEPLPSDS-----------IPERKTEPS-----------------------------------------------------------RMAPSVSEFPPSAAMKEI
Q2YC83       -------GPPSDASYKITSAS-----------APSPRLQGW-----------------------------------------------------------ETPLSLESMEPQILPVAS
B8FNR6       -------TPLPNFPARTPLTK-----------RPPRVERTA-----------------------------------------------------------PPRPEVDRAKKMEPLDAD
B5ECK8       --------PAVAAPSPFSAQL-----------WSEGAEPG--------------------------------------------------------------SARKDEAAAEAESPLE
C6DYF7       --------PAVAVPSPLSPQL-----------RSEDAEPG--------------------------------------------------------------GAREEAAAAEAESPLQ
A9WGY5       QS---------GPVVPAPPPA-----------LPASPV------------------------------------------------------------SLSLPPPP---------RAS
B9LL63       QS---------GPVVPAPPPA-----------LPASPV------------------------------------------------------------SLSLPPPP---------RAS
A7NNV5       VP---------ARQMVRPLS------------TTSADV------------------------------------------------------------ASLLLPAP---------AAP
A5URZ9       SP---------ARTTPVSLPN-----------VSSSDV------------------------------------------------------------SSDLPALP---------VAL
Q2W5V8       PL---------VSPPPVVRGGL----------PAKAPT------------------------------------------------------------SRALPPKA---------LSP
D3NZL7       P-----------GPPPAAPS------------PPTRPR------------------------------------------------------------RTAAAKPP---------AEP
C7C9P3       PD---------LAPILVAPP------------PEPAPA------------------------------------------------------------AAAPALPV---------PIP
B7KRG4       PN---------LAPILVAPP------------PEPAPA------------------------------------------------------------AAAPALPV---------PIP
A9W6M0       PD---------LAPILVAPP------------PEPAPA------------------------------------------------------------ATTPALPV---------PIP
C5AVV0       PD---------LAPILVAP---------------EPAP------------------------------------------------------------AAAPALPI---------PIP
B1ZEP6       PD---------AVPIPLAP---------------LPAFV-----------------------------------------------------------AVEPAAPM---------PEP
B1LWP1       PA---------RDPGPGASAGASS--------GVSGAGL-----------------------------------------------------------PDATVVPA---------PDP
B7JYH8       NN---------SLLNSANKDSKK---------IAIYTKQDGEKNKFSYKILTTTKENIS---------------------------------------QAIEEVNEID---------LLE
E0UB22       NN---------TVIRPPQ--FSE---------ITALPKATKPLDKIVQNLESKPQPSIPP-------T-----------------------------PLKSSLKDEE---------LLQ
C6DZA2       VP---------ASARPSAG-------------TKKEQR------------------------------------------------------------LN--PAAVS------------
B0TG84       SP---------ERQPDRIPFQPP---------MPAMPKR---------------------------------------------------------AQSALGQAP---------LLP
A5G943       P----------KPPLPVKTV------------PPRSNAPA----------------------------------------------------------QSEIDLS----------QLF
B9LZW0       E----------QTPAPVNTT------------APAPASP-------------------------------------------------------------QAVDWE----------RLH
B5EF82       Q----------AAPKAVSPP------------LPAPLAP--------------------------------------------------------------PSPAA----------LGV
C6E726       Q----------AAPKVVSPP------------PPEACAP--------------------------------------------------------------PPPAP----------LDV
A9WBT1       P----------APALRTRAP------------RPLERKV-------------------------------------------------------------TLFTEE----------AVV
B9LEA3       P----------APALRTRAP------------RPLERKV-------------------------------------------------------------TLFTEE----------AVV
B8G3A2       P----------TRPTRRRPT------------SPPASSD-------------------------------------------------------------PLFTDE----------TVV
A7NRP0       S----------TPSRKARPP------------VAATTAP-------------------------------------------------------------TMEEDV----------GRV
A5UPC3       R----------TASHGRRSP------------IAPITPKP-----------------------------------------------------------TSSNADA----------DHL
B7K4M9       -----------------PIK------------PPVVSSP-------------------------------------------------------------SNLVTV----------KKR
E0UAE6       ------------QKKIRPSN------------PTELTSL-------------------------------------------------------------AQAQTH----------LKK
B2J4R2       -----------TQFQSATLQ------------NPVKQSLGVKN----Y------------------------------------------------------SAIDTQ----------QIK
B1Z0W8       G----------NAASSAALA------------PAERPTAFEPRADMRAGSRTN---AAT---------AGHAGHAASATNAT------------------NATN----------ATN
Q0B461       G----------NASSPAALA------------PAERRTAFEPLADMRAGSLTAAATAAT----------AGHAAHAASATNAAS----------------AANAAN----------ATT
Q1BLP9       G----------DVRQPFAVP------------PPAVTTA--------------------------------------AIDAR--------------------ADL----------AVA
A0AYP9       G----------DVRQPFAVP------------PPAVTTA--------------------------------------AIDAR--------------------ADL----------AVA
B1K4W3       G----------DVRQPFAVP------------LPAVTTA--------------------------------------AIDAR--------------------ADL----------AVA
A9AP36       ------------IGGLATVH------------AADVDTAMADG----------------------------------PAAARAR------------------AADL----------PAP
A4JLK2       A----------TASGLPSSS------------PTSATRALHAVVPPPSG-----------------------------LDAARAAP-------------PTAFQPRA----------PSP
A1UYE3       A----------AHEEPFADA------------AGAARAAFDAPPGVSADAGRLA---------------ATARDAGAAPRASASLDHSFDTARPVFGTAAVAPGI----------AAP
A2S0C7       A----------AHEEPFADA------------AGAARAAFDAPPGVSADAGRLA---------------ATARDAGAAPRASASLDHSFDTARPVFGTAAVAPGI----------AAP
A3MB25       A----------AHEEPFADA------------AGAARAAFDAPPGVSADAGRLA---------------ATARDAGAAPRASASLDHSFDTARPVFGTAAVAPGI----------AAP
Q3JJX9       A----------AHEEPFADA------------AGAARAAFDAPPGVSADAGRLA---------------ATARDAGAAPRASASLDHSFDTARPVFGTAAVAPGI----------AAP
A3P8B0       A----------AHEEPFADA------------AGAARAAFDAPPGVSADAGRLA---------------ATARDAGAAPRASASLDHSFDTARPVFGTAAVAPGI----------AAP
A3NMV6       A----------AHEEPFADA------------AGAARAAFDAPPGGSADAGRLA---------------ATARDAGAAPRASASLDHSFDTARPVFGTAAVAPGI----------AAP
Q2T7Z6       P----------A-AEPFADA------------AGLARPAFDALPGAAADAGRLAS-------------MALARDAGAAPRASASLAHTFDTAWSAPARGAAASGI----------AQP
B2JNG9       S----------AAAERESAK------------PSLAQRAF-------------------------------TR-AP-------------------LRA----------PRP
B2T7T3       F----------RGATQFAQR------------ENSATQAY-------------------------------THKTP-------------------MPAA----------TPP
D8J0F2       P----------TPVRPDAAH------------RAPARRIV------------------------------------------------------------ASA----------ALG
A6STX7       P----------IQIKP-LPP------------ATPVRKPF------------------------------------------------------------ANV----------VPP
Q0JZJ6       -----------MAAPPRLPR------------PPASMR-------------------------------------------------------------------------------PVP
B3RBS6       -----------MAAPPRLPR------------PPASLRPL------------------------------------------------------------AARA----------PAP
Q46V25       -------------APAGMPP------------RPVSLR----------------------------------------------------------------VQ----------PAP
Q1LG87       -----------MIARARSAA------------TAPAKH----------------------------------------------------------------AA----------RAP
Q2KV72       -----------VAALSQAAP------------RPPAFS-----------------------------------------------------------------S----------AVR
B7V0Y7       -----------AAAVERASI------------RPSPPPP-----------------------------------------------------------AKPR----------QRL
Q02RH8       -----------AAAVERASI------------RPSPPPP-----------------------------------------------------------AKPR----------QRL
A6V180       P----------VVAAERTGS------------RPSPP-------------------------------------------------------------AKPR----------QRL
Q88MS8       ------------TLPAPVYP------------QPSVPLP-----------------------------------------------------------RSRR----------VLP
A5W896       ------------TLPAPVYP------------QPSVPLP-----------------------------------------------------------RPLR----------VLP
B0KRK9       ------------TLPTAGYP------------PPSAPLP-----------------------------------------------------------PPLR----------VLP
Q1IDW8       ----------------PLAP------------PPPRVVA-----------------------------------------------------------LPAR----------TVR
Q98PC8       -----------VRTPTPKVN------------KSEPLLP-----------------------------------------------------------FRP----------TIG
D8FCI6       G----------AGSRPDENK------------PKFIQKP-----------------------------------------------------------AFKK----------EVQ
C6BU38       T----------TLAQKAFIP------------APTFKQPE-P--------------------------------------------------------DIGKTK----------AVD
Q60AM0       -----------LAAKAVRRD------------APGEAKP------------------------------------------------------------SGP----------DLL
A0L7P7       L----------TEIAEEDEP------------LPPLFSLDDELL--------L-----------------------------------REETLLPEGNGEPE----------RIA
Q2W2W8       L----------HLVPPTPPP------------APP-----------------------------------------------------------------PA----------PSP
Q1NKB1       F----------RASKGEKRD------------TPAIKN-------------------------------------------------RYGVSPVPGSGKVP----------SSA
Q1NWT4       F----------RASKGEKRD------------TPAIKN-------------------------------------------------RYGVSPVPGSGKVP----------SSA
Q1NLW5       F----------RASKGEKRD------------TPAIKN-------------------------------------------------RYGVSPVPGSGKVP----------SSA
Q1D4W0       PGRLPAVSPHSPLPAIAARS------------RVTAELPT----------------V-------------GSVDSARPRITTELPAVATTPRAP-TVEVPAWPTL----------LPP
E3FYQ2       P-----MTP-SPRPSYLEQA------------ASPPSRP-------------------------------GSEPVRPPRLTLEVS--ATDPRVVRTTEVPGGAKA----------FPP
A7H826       ------------VPSAPPTV------------RHVPVAP---------------------------AAPPRLPTPAP--------I----P-AAQPP----------LAP
Q1D226       -----------AAPPS----------------RKRGT---------------------------------------------------------------ALPPG----------RAP
Q08NE7       L----------AEPPTPRPE------------RRRDSGV---------------------------FHTVTASGQGNDWS-------------REAAPSA----------ASP
Q1D8X4       P----------PTGWPQALDDS----------RARRARVPEGR------------------------------------------------LSLNEPGTSRP---------
Q08UZ6       -----------PFGTRLAASLP----------LAPLTRSP---------------------------------------------------LVRNPP-------------
A7H7I5       T----------PIRTRTPVPGPPEAGHPEPFDSAACGRYAQDR------------------------------------------------LRSAAPAAER-----------
Q1CX10       -----------TTAPGLPSGSESSTR-T----ALPGASAPSHPGGSAARTTTPAWSPPGAGGTSAQAEGTRAAGQPQASAGSVLGNTAGTGAPASRSGALPRAETPTHAPSA-----AAM
E3FZL3       -----------PPLPPLPP-------------LP--VAPPRP---P--T------PP------------------------------------------PRPPRPAPPSVT-----ERL
A7HCQ0       -----------TLRPPPPPG------------HVP----PIRS------------------------------------------------------HRAAPPGPP-----------
E3FRV5       ---------LDVASLQAASS-----VRWMSPPTPPAPRMPE------------------------------------------------------PPREPLG------------M
Q08UV4       ---------LDVASLQAASS-----VRWMSPPTPPAPRMPE------------------------------------------------------PPREPLG------------M
A7HCX6       ---------AWVQAIQDASER----VAKVSAAAHAAAAQPS------------------------------------------------------LREEALPRSGAAPREDGARV
C6C1V4       -----------AQLESTPADFVDLKFP------EPEIPVP-------------------------------------------------------EVESVPEPP--------VIPR
D2Z5P5       ------------EIEPEVEA------------VSDVWFD-------------------------------------------------------------------------------
D0LTR4       -----------LRLYARRRS------------TTLKLRTR-----------------------------------------------------ARSQRTP----------ERP
Q08XU2       ------------APRPARSG------------PPRMTPP-------------------------------------------------------------------------------
A0L8P6       ----------LALSRGQTKP-----------ATPYVASPPAG--------------------------------------------------YVAPSITNPHPP------LVAG
D0LUD0       ---------QARVLSPRARG-------VRRQRAPAAASPP-----------------------------------------------------ASTRASASG--------NAD
D3PC59       ----------ITDKKERYLR------------EIKSLELLN-------------------------------------------------------------------------N
D3P9S2       ----------HPFVTDIKVR------------TRKISEKPE-----------------------------------------------------TELFEKAVN-------FMFN
```

```
                  850       860       870       880       890       900       910       920       930       940       950       960
                    |         |         |         |         |         |         |         |         |         |         |         |
E. coli     ------------------------------------------------------------------------------------------------------------------------
S. typhi    ------------------------------------------------------------------------------------------------------------------------
Q12AR4      ------AFAQSLYEQGRYAEVVDTLLAALAEH------APLTEACSLLARALANQGRLADAL---TWCDRWIVTDKLDP------AGHYLRAGVLLEQG---ELEQARSSLQRATYLEPG
Q2LR61      GEEALHESALRLFKEGRYSECEQILTSLLEQY------PESFHGQALMARLHADQGRLPEAQ---EWCEKAISNNKLDA------GCHYLLAVIFIEQR---RFGDAEQSLHHALYLDPD
Q2YC83      -------SSFTQLTS-----VQEVAGQSANQE------RSNTLYR--EARRHADGGDLPRAV---KQCEEAIAANKLNP------AAYYLLATIHQELG---QAKEAMRCLMNTLYLDPD
B8FNR6      R---MLDKALELVEKGNYDDAVNCLLEIISAADAKAGMKPDSRAMALLARTYANMGKLEEAR---KWGGKAVDADKLNP------GHRYLLATVYAELG---DSEKAADLLQASLFLDSR
B5ECK8      R-------AFALYSAGNYEEAARMAMSTPASA-------DKAEALALAARSYANIGRFDPAR---DCCEAAVALDRLSA------PNHYLLSIILEQQG---DGEGAVRSLRNALYVDHD
C6DYF7      R-------ARAFYRAGLYEKAARMALDTPAGV-------EKAEALALAARSYANVGRFDPAR---DCCEAAVELDRLNA------PNHYLLSIILEQLG---DGEGAVRSLRNSLYVDHD
A9WGY5      S------APISSGVLSSASPPAPVRAE---------------DLLHVARTAADRGDWQQ---AQTQIEALIKAHPLFA------PAYHLQGQIAEHLG---HLEAALAAYRRSVYLDPR
B9LL63      S------APISSGVLSSASPPAPVRAE---------------DLLHVARTAADRGDWQQ---AQTQIEALIKAHPLFA------PAYHLQGQIAEHLG---HLEAALAAYRRSVYLDPR
A7NNV5      S------PSFVSDTPVADNAAP--------------------DTLAVARQAADRGDLAT---AMRLCETMVQHQPLSA------EAHLLNGQILEQQG---RLDEALAAYRRAVYLDHS
A5URZ9      S------SSEPAPVRAADADAQAF------------------DDLQAARQAADRGDLEE---ALRLCATIIQREPLRA------EAHLLTGQILEQQE---RFDEALAAYRRAIYLDHT
Q2W5V8      R-------GSTSARPGSAAPAAAAGGAG------------DFDRLTVARNLADSGQWEK---AAAACDAIIASPLDA------WAYFYRAMVHEQLG---EDEPCEKALRRAIYLDRR
D3NZL7      P-------AKPPTKPDPSAPAAANDRS-----------------------T---------ALETCLVQVEANRMDP------AVHYRLGLVEEELG---VGDPVA-AFKRALYLDPR
C7C9P3      R-------PAPPAIAHPLSPGD---------------------LLAEIRVLADAGETAR---AWRRLHEEIDGYATDS------PLRYYEGLLALDLG---REREAERALRGALFLDRG
B7KRG4      R-------PAPPAIAHPLSPGD---------------------LLAEIRVLADAGETAR---AWRRLHEEIDGYATDS------PLRYYEGLLALDLG---REREAERALRGALFLDRG
A9W6M0      R-------PAPPAIAHPLSPGD---------------------LLAEIRVLADAGETAR---AWRRLHEEIDGYATDS------PLRYYEGLLALDLG---REREAERALRGALFLDRG
C5AVV0      ---------APAAIAHPLSPGD---------------------LLAEIRVLADAGETAR---AWRRLHEEIDGYATDS------PLRYYEGLLALDLG---REREAERALRGALFLDRG
B1ZEP6      IGI-PIPLPAEPATEHPLSPGD---------------------LLAEIRVLADSGETAR---AWRRLHEEIESYATDP------ALRYYEGLLALDLG---REREAERALRGALFLDRG
B1LWP1      -------VAAPGTAPVPDAPGTRDHGAV-LD----------EACDLDRIRALADSGETGA---AWRALRAALDRDPTDT------ALRFYEGLLARTLG---RDAEAERALRAALYLDRS
B7JYH8      EVK-QLIEQKNYSFSIKKLHKILEKYP--NS----------FAANYLMAEIYANLGKYEE---AIDYCHQATTIDSLAV------TPHHLLVQIAEERG---ELEEAKRLLRKIIYLEPY
E0UB22      EAE-SLIAAKSYYLANKKIEEFLSISP--RS----------FSGHYLLAKIQANLGKHEA---AIQACKKAIEIDSFSV------APYHLLVQISEEKG---DLEEAKTLLKKIIYLEPC
C6DZA2      --------AKRLFAGHAAKPVAAGVLP--HD----------LDRCLEQARRCADQGKYHD---AARICRDVIRYHTLSP------LPYFLLAQISEANG---DAWSAKQCFKKAIYLDPS
B0TG84      ---------RDLPPQKPAIANVAGEAPKLSD----------LDRLRDKARDFANRREYDR---AEACCRAVIAVNPLDS------EVYYLLGLIRLEQG---DWSGAHAFFQKVLFLAPD
A5G943      R------KARQMFEEEHFTEAMQLLKEVIRHQ------PDHAGALITQGFILANDGHFQEA---LAACGKALGIDDLLA------EAYFLKGLVLDMSD---NLTEAAEEYRKAILLEMN
B9LZW0      L------HAQELFREENFSETARLLQDILQHR------PDHTGALILYGFTLANDGRFTEA---LAACEAALKTDDLLP------EAYFLKGLVYEMTD---RLPEAGEEYRKAILLQHD
B5EF82      A------AARELFDQEEFDRAQELLDRILAED------PSNAAALVLVAFILAGKGELQEA---LKTCSRALELNDLLP------EAYFLKGVILDAED---RLAEAADEYRKALLLEHE
C6E726      E------AARELFDREEFDRAQELLDRILAED------PANAAALVLVAFIQAGKGALQQA---LKSCSRALELNDLLP------EAYFLKGVILDAED---RLAEAADEYRKALLLEHE
A9WBT1      Q------EGRQLIENGQIDTALELFARVPLAG------RHAPMVLALAAQAHANRGDLDLA---LAEARRALELNPLLT------EAYLLLGLIYERQQ---QMPLAIRHLERARYLNRD
B9LEA3      Q------EGRQLIENGQIDTALELFARVPLAG------RHAPMVLALAAQAHANRGDLDLA---LAEARRALELNPLLT------EAYLLLGLIYERQQ---QMPLAIRHLERARYLNRD
B8G3A2      V------EGRRMIESGQIEAALELFAHAPLAG------RYAPAVLALTAQAHANRGELDLA---LAEARRALELNPLVT------EAHILLGLIYERQQ---QFTLAIRHLERARYLNSD
A7NRP0      E------QAQALIDAGRIDEAMDLLRSIHPNS------SLAPRALVLVARVHADRGELDLA---IAEARRALEIDALRS------DAYLLLGTIYARQG---QGNEAIQALERARYLDPD
A5UPC3      D------RAQALLDAGRLDDAMEVLRTIPPNS------SLAPRALTLVARVHANRGELDLA---IAEVRRALEIDALRD------DAYLLLGTMYVRQG---QWHDAIQSLERARYLNPD
B7K4M9      K------PFPSNPSPSPESQQTQ------------------LSALKSARKLANQGQLSEA---TEQCQNYLSQNPADA------SAYLLLGEIEQAKG---NIDKAQRYFQKALYLQPD
E0UAE6      G------QTISPLSLANHPQSPP------------------KNPLAQARDLADSGALEQA---ASLCRAYLNQTPSDP------QAYLLLGQIYQALG---SEIEAEQYFQKVVYLDPN
B2J4R2      E------SNISASSNRSSSESTP------------------SVDLQTVKKLADEGRSPEA---ITLCKSYLIHHPTSA------AAYLILGALYQADQ---QNHQAEQCFQRAIYLEPT
B1Z0W8      A------ANAANTTNAANAANVP--APAPPLA-------DAATPTLEEAQALANAGAFDEA---ERVLARFSAHAGPHA------DAFYLNGLIADACG---RVAEAGDFYRKALYLRPT
Q0B461      A------AHAASAASAAKAANAANAALPPPPA-------DIATPTLEEAQALANAGAFDEA---ERVLARFSAHAGPHA------DAFYLNGLIADACG---RVAEAGDFYRKALYLRPT
Q1BLP9      A------AAAAAPANDGIAPTLD-----------------EAPTLEDAQALANAGAFDEA---ERVLAQFSARVGPHA------DAFYLNGLIADARG---RAAEASDFYRKALYLRPT
A0AYP9      A------AAAAAPANDGIAPTLD-----------------EAPTLEDAQALANAGAFDEA---ERVLAQFSARVGPHA------DAFYLNGLIADARG---RAAEASDFYRKALYLRPT
B1K4W3      A------AAAAAPANDGIAPTLD-----------------EAPTLEDAQALANAGALDEA---ERVLAQFSARVGPHA------DAFYLNGLIADARG---RAAEASDFYRKALYLRPT
A9AP36      A------RPVAAMPVTDTPADDA-----------------HTPTLEDAQALANAGAFDEA---ERVLAQFSARVGPHA------DAFYLLGLIADARG---RAADASDFYRKALYLRPT
A4JLK2      F------EPVAPPPADPLARARS--PSPAPAN-------AVDAPTLEQAQALANAGAFDEA---ERMLARFSAHAGPHA------DAYYLNGLIADARG---RAEEAGDFYRKALYLRPT
A1UYE3      A------FASRALSATALAGAFEADGTHAAA---------VDEAPLDAARRLADAGALDAA---QQAVRASIEQSGPSA------EAFYLLGLIADARG---RSDEATDCYRKALYLEPT
A2S0C7      A------FASRALSATALAGAFEADGTHAAA---------VDEAPLDAARRLADAGALDAA---QQAVRASIEQSGPSA------EAFYLLGLIADARG---RSDEATDCYRKALYLEPT
A3MB25      A------FASRALSATALAGAFEADGTHAAA---------VDEAPLDAARRLADAGALDAA---QQAVRASIEQSGPSA------EAFYLLGLIADARG---RSDEATDCYRKALYLEPT
Q3JJX9      A------FASRALSATALAGAFEADGTHAAA---------VDEAPLDAARRLADAGALDAA---QQAVRASIEQSGPSA------EAFYLLGLIADARG---RSDEATDCYRKALYLEPT
A3P8B0      A------FASRALSATALAGAFEADGTHAAA---------VDEAPLDAARRLADAGALDAA---QQAVRASIEQSGPSA------EAFYLLGLIADARG---RSDEATDCYRKALYLEPT
A3NMV6      A------FASRALSATALAGAFEADGTHAAA---------VDEAPLDAARRLADAGALDAA---QQAVRASIEQSGPSA------EAFYLLGLIADARG---RGDEATDCYRKALYLEPT
Q2T7Z6      T------FSSRALGATALAGVAETDAATDAAS------RAAGDAPLDAARRLADAGALDAA---QQAVHASIEQAGPSA------DAFYLLGLIADARG---QGDEATHCYRKALYLEPS
B2JNG9      V------QAMPPDASPQPAAERS---------------------LDAARRHADAGDFDAA---ERLAHQHALVHGPNV------DAFYLLGLIADARG---RGDAAADFYRKALYLDPA
B2T7T3      A------FNPSLPPVTAPTNAAR------------------DTLQAAHALADGGRLAEA---ATAISAYLEHHAPHA------DAFYLLGVLADANG---DASLARSQYRKALYLDPQ
D8J0F2      A------VGTATAARPASPATAL------P------------AGLLERARALADQGRFAQA---GELCEQAVQQQGPSA------EAFYLQGLIHDAGG---QQELAQRCYRKALYLEPE
A6STX7      V------VPIATPVAAPSGET---------------------LLTRAMQCANQGELAEA---DALCKEHVQQNGPNA------AAYYLMGLISDARQ---DSGEALQFYRKTIYLQPN
Q0JZJ6      V------TPPPLRPAAPAPGNDQ------R------------AALAAIAAQADRGELEAA---TAACLALLERTADAN-----ADAYCMLGVLHDAAG---RAAQAHAAYRKALYLDPA
B3RBS6      A------AMPAAPLAAPSAADAH------A------------AALAAIAAMADRGELEAA---TAACLALVDRAAPDTGGAAMADAYGMLGVLHDAAG---RVAQAHAAYRKALYLDPS
Q46V25      A------VREPAPVRAPRAAAPT------P------------TPLARISALADRGDLAAA---TAACEALIASQGPSA------DACCMLGVLHDAAG---LTSSARDAYRRAIYLDPG
Q1LG87      D------VAASNPSAQPASAPSR------D------------AALAAIAAMADRGELDAA---LAACQLFLTEHGTSA------DGWCLLGVLHDASG---RATEAHAAYRKAVYLDPG
Q2KV72      L------LPVPPPASPPVDAAQR------D------------AALQSVAALADQGRLDEA---WAASEAFMAKHGASA------GIWYLRGLIKDAGG---QVEAAHDAYRKALYLDPA
B7V0Y7      S------SLVPPASGQP--LASP------V------------GEFDEIARLADAGQHREA---RAACERQLAARGPSA------TVFYWLGLLSDVAG---QEQEAQDFYRKALYLEPQ
Q02RH8      S------SLVPPASGQP--LASP------V------------GEFDEIARLADAGQHREA---RAACERQLAARGPSA------TVFYWLGLLSDVAG---QEQEAQDFYRKALYLEPQ
A6V180      S------SLVPPASGQP--AAGA------G------------GEFDEIARLADAGRHDEA---RVACERQLAAHGPSA------AAFYWLGLLSDVAG---QVREAQDFYRKALYLQPQ
Q88MS8      V------AARPARAREHS-HEGA------S------------ELLAGIARLANAGASEQA---RSECQRYLSQYPPSA------QVYYWLGLLSDTEG---DAQQALSHYRKALYLEPQ
A5W896      V------VARPAPAREHR-HEGA------S------------ELLADIARLANAGASEQA---RSECQRYLSQYPPSA------QVYYWLGLLSDTEG---DAQQALSHYRKALYLEPQ
B0KRK9      V------AARPAPAREHK-HEGA------S------------ELLASIARLANAGASEQA---RGECQRYLGQYPPSA------QVYYWLGLLSDTEG---DAQQALTHYRKALYLEPQ
Q1IDW8      P------IPVPAPVIEP--HESE------A------------QLLGLIARHANTGDSEQA---RAGCERYLRQFAPKA------QVYYWLGLLSDTEG---DAAQAITHYRKALYLEPQ
Q98PC8      N------EGRRQAPAKPLAQAS--------------------ESLIAVERIANAGRVKEA---QQVALAHLKEFGPSA------EIFYLLGLVQDADG---AAPEAAQSYRKALYLAPN
D8FCI6      S------SERTDEPRKPEPDTIDAAMEN------------PADLFPKVKELADHGKLLDA---LELCEIFLNENPVHV------EAHFLMGLILEALH---DAERAEAFFNRAIYLNPE
C6BU38      A------AEKSVEKIESVEPTAG-------------------SSIEEIKALADRGSTTKA---LSMCDELLRQAGPEP------ELFHLCGLLHEAGG---NISMAEEFYGKALYLEPD
Q60AM0      S------GGRETGRAAPFDEG---------------------AVLGTARALADGGNYQAA---ERLCQSHLASHPHDP------EVHALLGIVMSAAN---RDDEALRYFRQALYLAPS
A0L7P7      V------PSMDQLVALLKEKRYGEALAGVELA------CAQGGGQIQAELLRLKGAVLLNQ---RQFVQAELALKGALQQDEWSLDALLLLGQTARWQG---QTKQAIAWFQKAVYLDPS
Q2W2W8      G------ITVEEARALIRDKRFDR--IDLLLG------ARNRQLPADATLLALEGYARLMS---RDFAAAAELGARALAADEWSVDGLVLLGLAAKWRD---AAQDAIGWFKKAVYLRPE
Q1NKB1      Q------DLFGEALAAARQQHFDQ--ARELLQ------RLLEREPGSGRAHALDGCILVEL---EKFDQARQCGLTALEHDPLCLEAYLLLGISARQRA---DYQEAGHRFREALYIERA
Q1NWT4      H------DLFGEALAAARQQHFDQ--ARELLQ------RLLEREPGSGRAHALDGCILVEL---EKFDQARQCGLTALEHDPLCLEAYLLLGISARQRA---DYQEAGHRFREALYIERA
Q1NLW5      Q------DLFGEALAAARQQHFDQ--ARELLQ------RLLEREPGSGRAHALDGCILVEL---EKFDQARQCGLTALEHDPLCLEAYLLLGISARQRA---DHQEAGHRFREALYIERA
Q1D4W0      A------ERLAMAVRKMAQGDFSAAIAGVQRL------LADEPSDLDGLLTLGNLFSLTG-----RIPEAREAFAQAIQREPLCVEARVFGGVAALQAG---ELSEARSELSKALFLEPT
E3FYQ2      V------ERLKQAVRKMMQSDFTAAIHDVEKL------LIDEPGHLDALLTLGNLYSLTG-----RIAEAREAFAQALVREPLCVEARIFGGVAALQAG---NLVEARSELGKALFLEPT
A7H826      Q------EYLDAAVALFAEGRFGAARELLERL------LEKGGEDLAVRLTLANLYGILR----QTDRARECYVAALQLEPLSAEAHLFFGIHLLAEG---DTEPAALELSRALFLDPD
Q1D226      A------RARTEAHHGFSVP--------------------APTEVEGVRGPVATAP-------------EPS------PEACAEADLLFACVLDGAASGVSDAVAERDLRRCLTLDPD
Q08NE7      A------DSRRDSGRFAAMPSGPARDSGRFAA------MPAVPARDSGRFATVATVALRDSGRFPAVGGGLEPAGGLSSLELMHAEADALFAQILEGAG--ETDVQKEEYLRRCLSLDPE
Q1D8X4      --------AWAASTPDAATPAVPRSVAVDALA-------AEEVPPLERAIIAARAGHFEEA--------EALAREAAK---ALVPEAYLLLSMVAEVRG---DLNGAVEAVRKALYLEPR
Q08UZ6      ----------AAPVPPAQEAPSP-----------------EDDAMRQALVAAREGKYDVV--------ETLAREAAR---KLVPEAYLLLAMVAETRG---DLNGAVDCVRKALYLEPQ
A7H7I5      --------SRGTPTPTASATPTP------------------APGFEQARDAARRGDVEFA--------EQLAREVAER--HLCPESFLLLAMAADARG---DLAGAIDALRRALYLEPG
Q1CX10      H------AARQALARGHWREAAQHLGALDS---------DADTAALAVRALANLDAAAAV--------YACTEAATRH--PLVAGLRYLESLLLGQG---RAADAERAVRQALYLEPT
E3FZL3      E------PARQAMERGDWSHAAQLARAQQE----------GPETAAMAIRALANVNPEAAA--------AACAEATARF--PLAAELRYLEAMVLLGLG---RLAEAERAARQVVYLEPA
A7HCQ0      ----------TTLSRDPELAAAHLLVD-----------------AAHLTEALAAVD-----------------DALARR--PLDAEAHFEKAVVLTELG---RLEEAADACRRARYLSRG
E3FRV5      E------RAWRLLEEERYAEAQAWLEQLPEPD------REQSSARLLRAVLHFQGGHFPEA--ER-VAESLVATGRAEA------AVYYLLGLCREQAG---DEGGARNRYARAVHLEPT
Q08UV4      E------RAWRLLEEERYAEAQAWLEQLPEPD------REQSSARLLRAVLHFQGGHFPEA--ER-VAESLVATGRAEA------AVYYLLGLCREQAG---DEGGARNRYARAVHLEPT
A7HCX6      A------LALELLRRERFADALAALGGRAPAG------GEDVDALLLRAVLLTSSGDLEGA--EG-VCARILERDELNA------EAHYLRALCREHAG---DLAAAADHDRYALYLDPT
C6C1V4      AGNDGAALLKQAGEVRMHGDTLKAVSLYKEVVEHENPREIKAAAFLGIAGIKADSGLTDEA---ALWCAKALELDRVSP------CAHFLLGQICFQQG---DLSTALAHMRNAVFLDSE
D2Z5P5      ------LESETEPEIEPEIEAVP------------------SCDGDLIRSLADRGMSDEA---LELCLSSENSTDP--------YVHYLMSVIYQDRG---ELDSAKDCLRKALFLQPS
D0LTR4      S------PTVAPPAPAHAPAAAPPAPTPPGS----------RSEALGRARALADAGKSAEA---LAELKLLLSEDPLQA------DAYLLRATLLQAMG---QHDAVIADTKRALLLDRK
Q08XU2      --------PVASPPPEPPRSP-------------------------------------------------------------------SQFHLEALERIEQG---DVPRASLMLEALVRQFPD
A0L8P6      EALPTDLELQKLLIEGQFSTVVTRTERLPSTCP--------DRFFLQRAQALTALGQWQAA---QELLQLFLKDHGMND------EAHFLLALVFLEKK---DEKGAEEELRKTMMLNRD
D0LUD0      PG--SGRDAATQPATPSDAPREDAAQSP-E-----------VADLLQRACELEKLGQLDEA---LQRLTAAANRAPLAA------AVHLERGLLLKRLT---RLDEAVHALRAARFLDAD
D3PC59      KE-----EAINLIENVQAKNEE----------------FDKELLIEEIILYLNNDDLFIIEKKLREYRDYLSDD---------EFYFIKGLVCYKEK---NYDKAVDYLRKAALLN-D
D3P9S2      DN----FKEASILFKKILNELNPTN----------------LRAKIFYLICLIKENQLDEA---ERFITFSLEENIFSY------EIFLIKGIIHFLKG---NYKTSKNELRKSIYLKTD
```

```
                    970       980       990      1000      1010      1020      1030
                      |         |         |         |         |         |         |
E. coli      -----------------------------------------------------------------------------
S. typhi     -----------------------------------------------------------------------------
Q12AR4       FVLANFALGNLARRRGKTGEAGKHFANTLHLLQGYQPGDLLPESDGLTAGRLAQTLASLTDP-EIAP----------
Q2LR61       FVLAHFTLGNLVREEGRKTLSSKHFEHALLLLQNYQPEEILPESDGVTAGRLKDIILMADLGRSNAPGA--------
Q2YC83       LVLAHFALGSLCLTQGKSREAKRHFDNALSLLNGYSSNDILPDSGGLTAGDLAHFITSIRICDSIGNKE--------
B8FNR6       FILAHFTLGNTMKKQGKANEARRHFKNALSLLLDLDSDAVIPHSGGMTAGRLTEIVRSMILGD--------------
B5ECK8       FLLGWFALGNLCRQRGELREAEQSFANALRLLQRRDPHEVLPEAEGMTAGRLMQLISDIA-----------------
C6DYF7       FLLAWFALGNLCRQRGEPREAEQSFANALRLLQRRDPHEVLPEAEGMTAGRLMQLISDIA-----------------
A9WGY5       LIVGYIGMAHVYTQLQQPDAARRTLRSAQTLLGALADSQVVDDATGSTAAELRNY---VRLLMEKVDLR--------
B9LL63       LIVGYIGMAHVYTQLQQPDAARRTLRSAQTLLGALADSQVVDDATGSTAAELRNY---VRLLMEKVDLR--------
A7NNV5       LYLALLAMAGILHRTGHVDEAYRAYRRLLRALATLPPHAPVPSFDNATAAELQMF---IQRQMEALRR---------
A5URZ9       LYLASLAMAGILQRTGHTDDARRAYQRLLRSLATLQPDAPVPSLNGATAAELQMF---IRNRMAALGS---------
Q2W5V8       LVLPHYHLGLFLARKDDSTGAERSFRNAQALLAGLADEQPVNPGEKIAVGQMREA---VAMHLKLLGGA--------
D3NZL7       FVLADYHLALAYWRRGRLVPAQRHFRNAREALADRPDGETVTEGAGLTVQELRGM---LDLWLTGEEG---------
C7C9P3       FVMAHFQLGLLLARVGRRGEAARALDNALRLAQGLPPETILPEGDGVSAARLAES---ARRALAGITGRDA------
B7KRG4       FVMAHFQLGLLLARVGRRGEAARALDNALRLAQGLPPETILPEGDGVSAARLAES---ARRALAGITGRDA------
A9W6M0       FVMAHFQLGLLLARVGRRGEAARALDNALRLAQGLPPETILPEGDGVSAARLAES---ARRALAGITGRDA------
C5AVV0       FVMAHFQLGLLLARVGRRGEAARALDNALRLAQGLPPETILPEGDGVSAARLAES---ARRALAGITGRDA------
B1ZEP6       FVMAHFQLGLLLARAGRRGEAARALDNALRLAQGLSPETILPEGDGVSAARLAES---ARRALAGITVSEA------
B1LWP1       FVMAHYHLGLLLIALQRPSEAVRALDNAVALSQALGPDTALPEGDGASAGEIAASAAAARAALGGVGSRRS------
B7JYH8       SVAAYLNLANIYQQTNHQEKAQKTRKNALKVLQKLSPDTTIPELGNKTVQDCIIEITNLTN----------------
E0UB22       SVSAYLNLANLYQQEGDQKRSEKMQQTALTILKKLPNDTQVPELGNITVQELLTQLE--------------------
C6DZA2       LIAPHLELGEMYLSEKKYELAAKSIRSARELLKVMPPGKFIALYSETTALELLRHADDLLDGLNKKEKTSDRT----
B0TG84       HGPARIEMAHVRLLLKENDQ---------------------------------------------------------
A5G943       FVMPHYQLSWLYARMGKDKERQRELNNTLNILAKLGKN---STIPYSGGLSREAFIEQLKRELEMAG----------
B9LZW0       FVMAHYQLAQLYARLGRKKERLRELNNSLRIVAGLKAK---AAIPYSGGLTRELFMDQLNKELAKAD----------
B5EF82       FVMPRYHMGRLHLRLGRQAEAAREIRNSIRILARHDGN---DTVPFSGGLTRAVCMMQLQNALAQVA----------
C6E726       FVMPRYHMGRLHLRLGRQAEAAREIRNSIRILARHDDN---DTVPFSGGLTRAVCMMQLQNALAQVA----------
A9WBT1       HPLVAFHLAECYRQNRQITEAIREYRNAERLLNNLPPD---QLLEGVAVQWLIESCRRWIGRLEGER----------
B9LEA3       HPLVAFHLAECYRQNRQITEAIREYRNAERLLNNLPPD---QLLEGVAVQWLIESCRRWIGRLEGER----------
B8G3A2       SPLVAFHLAECYRQTDRVADAIREYRNAEHLLHSLPPD---HLIEGVAVYWLAESCRRWIRRFEGER----------
A7NRP0       AALVSYHLALAYRQAGRQEQAMREFRSALSKLARHRSE---DLIEGVEVGWLRTTCEQHLGM---------------
A5UPC3       AALVSYHLAMAYRQAGKKELAAREFRSALRKLAAYRAE---DLLEGVEVGWLRATCEQHLASLET------------
B7K4M9       YQEALMHLALLRESQGDAKGAAVLKNRIQRLQNP-------------------------------------------
E0UAE6       CEEALIHLALLKESRGDLSGATLIRQRIQRLFKLRDNP---L-----------------------------------
B2J4R2       SYQALVHLALLKEHQGDTVGAKIIQQRIQRLQSSLRTE---A-----------------------------------
B1Z0W8       HHEALTHLATLLDVGGDGAGAQWLLERARRAAG--------------------------------------------
Q0B461       HHEALTHLATLLDVGGDGAGAQWLLERARRAAG--------------------------------------------
Q1BLP9       HHEALTHLATLLDVGGDRAGAQWLLERARRSAG--------------------------------------------
A0AYP9       HHEALTHLATLLDVGGDRAGAQWLLERARRSAG--------------------------------------------
B1K4W3       HHEALTHLATLLDVGGDRAGAQWLLERARRSAG--------------------------------------------
A9AP36       HHEALTHLATLLDAGGDRAGAQWLLERARRSAG--------------------------------------------
A4JLK2       HHEALTHLATLLDVGGDGAGARWLLERARRAVG--------------------------------------------
A1UYE3       HYEALTHLATLLDIAGDRDGAQWLMQRARRAAQYESAA---SVTDTDDGEPRGTHGTRRR-----------------
A2S0C7       HYEALTHLATLLDIAGDRDGAQWLMQRARRAAQYESAA---SVTDTDDGEPRGTHGTRRR-----------------
A3MB25       HYEALTHLATLLDIAGDRDGAQWLMQRARRAAQYESAA---SVTDTDDGEPRGTHGTRRR-----------------
Q3JJX9       HYEALTHLATLLDIAGDRDGAQWLMQRARRAAQYESAA---SVTDTDDGEPRGTHGTRRR-----------------
A3P8B0       HYEALTHLATLLDIAGDRDGAQWLMQRARRAAQYESAA---SVTDTDDGEPRGTHGTRRR-----------------
A3NMV6       HYEALTHLATLLDIAGDRDGAQWLMQRARRAAQYESAA---SVTDTDDGEPRGTHGTRRR-----------------
Q2T7Z6       HYEALTHLATLLDIAGDRDGAQWLMQRARRAAQYESAA---SVTDTDDGEPRGTHGTRRR-----------------
B2JNG9       HYEALTHLAVLLDIGGDRAGAQNLVQRAQRSAARAAQS---TDASADEAQRSRGFHASRRA----------------
B2T7T3       HAEALAHLATLLELEGDRDAARLLMQRASRAQGAQRG----------------------------------------
D8J0F2       HQAALLQLAALLQAQGDASGAQRMRQRAERMAKPGEGA---HG----------------------------------
A6STX7       HYEALTHLAALLAAQGDTAGAQLMQQRAQRVAASATSA---QEQPHA------------------------------
Q0JZJ6       HQESLYHLAALLDTEGDHAGAMHLRQRAQRHTRKHHG----------------------------------------
B3RBS6       HQESLYHLAALLDTEGDHDGATRLRQRAQRHTRKHHG----------------------------------------
Q46V25       HQEALHHLAALLDTEGDHAGAARLRQRAQRHTRTA------------------------------------------
Q1LG87       HEEALYHLAALLDSAGDAAGAHRLRERAQRHARMSHG----------------------------------------
Q2KV72       HRQAMLQLAALLHAEGKQEASERLSARARRLGP--------------------------------------------
B7V0Y7       HAEALAHLAALLAARGDHAGARRLQQRAARGVNKDG-----------------------------------------
Q02RH8       HAEALAHLAALLAARGDHAGARRLQQRAARGVNKDG-----------------------------------------
A6V180       HAEALAQLAALLAASGDHAGARRLQQRAARGVNKDG-----------------------------------------
Q88MS8       HPEALVHLAALLAAQGDLAGARRLQERAARAGRESER----------------------------------------
A5W896       HPEALVHLAALLAAQGDLAGARRLQARAARAGRESER----------------------------------------
B0KRK9       HPEALVHLAALLAAQGDLAGARRLQERAARAGRESER----------------------------------------
Q1IDW8       HPETLVHLAALLASQGDVAGARRLQERAARAGRESER----------------------------------------
Q98PC8       HREALVHLALLLRKQGDHTGAEALAGRLGRVQKRSGS----------------------------------------
D8FCI6       HVEALNHMAFMELHRGNKAGAERLRKRAQRIGMGGVER---RSTENQSSGRRYGK----------------------
C6BU38       HMESLVHLALLLENRGDLRKAEIMRNRARRAEKRNEAG---------------------------------------
Q60AM0       HNESLLHLAALYERRGDEERARHFRNRSAAAEGEP------------------------------------------
A0L7P7       HWLAHYFLAELYAIEQQSSLAQREYRLVLRQLDERRPV---EGTLLALLALAVPPKQISFLCQQKLAKLAVHS----
Q2W2W8       CWPAHYYLAELYRSADSPDLSRRSYRVALQQLSGQPEP---DGGLALPLGLPVA--EVLFLCERQAGTARSAQR---
Q1NKB1       CWPAHFYLAETAAALGDADRARRAYHNALEVLQHQGE----EARRRSFFPLSCNPAHFVNICRHKLARLAN------
Q1NWT4       CWPAHFYLAETAAALGDADRARRAYHNALEVLQHQGE----EARRRSFFPLSCNPAHFVNICRHKLARLAN------
Q1NLW5       CWPAHFYLAETAAALGDADRARRAYHNALEVLQHQGE----EARRRSFFPLSCNPAHFVNICRHKLARLAN------
Q1D4W0       LAIGHYLLAQVHERTQDHEAARRSYRNAI-AQLRFPQ---RPLAGHYPEMPDSADAISRAARYALAALEEQPLR---
E3FYQ2       LALGHYLLAQVQERTQDRDGARRSYRNAI-AQLRFPQ---RPLAGHYPDIPDSAEAIARVARYALAALEEEGN----
A7H826       LALAHYWLGRCREAQRDPERARLAYRNALTAHARWPEGKRQAFLGYYPDVPEDGAAFVRAAQYALAAL---------
Q1D226       HAAARYLLGLLLEQCRRTAEATAEYRRALQALESGRS----RPVAFFLNPDRLRVACAHAAERLEFSIQKR------
Q08NE7       LAAARYLLGMMLELREEFPEAAGEYRRALRSLEEGKA----RATPFFLNPARLQVACARAVERMEVGKSR-------
Q1D8X4       LALGHATLVALYGRMDRPEDAERARQNALRALDGLDDEHPLRGVETMTAGGLRQALAPVEQAGWLGVR---------
Q08UZ6       LALGHATLVTLYTRLERREDAERARQNALRALDGLDDEHPLRGVETMTAGGLRQALSARGRAGWQGAP---------
A7H7I5       LAMAHAALVPLYARVGRHDEAARARRNALEAVEGLDDTAPLRGVEPITVGALRSALGAVHRPQAGAGRSEA------
Q1CX10       LVVAWLILGRVLRRHGDTSGALKAWREAEQLCNALPPDAPVPHADGENASRLAEVARGERSRMEAALAAGEELS---
E3FZL3       LAVAHLALGHVLRRRDDLAGALRAFRAAEELCAALPPDTAVPLSEGERAGSLARVAHNERVRLETMMTREER-----
A7HCQ0       QPFVHFFTGLLRLRRGDTAGASRALRTAASLAQRMPAAAPVRLSHGMTAGALVEGARFHLSRLAGDGPSRP------
E3FRV5       FALGHLRLGILARRAQEATPARVALRLALTLLAHEQPLHLTLFGGGFGRHGLMQVCQQELRACAEVP----------
Q08UV4       FALGHLRLGILARRAQEATPARVALRLALTLLAHEQPLHLTLFGGGFGRHGLMQVCQQELRACAEVP----------
A7HCX6       FAMPRLHLGLLAKRGGDLEAARRELSRALVLLAGEEGSRILMLGGGFTRDALLDVCRAELNACGGIP----------
C6C1V4       FIMAHFLLGNIYLEQNDSNGALRHFRISMQELDKMDQDDPVPCSDNVTAGRLMEMVRLVQQQLVQ------------
D2Z5P5       FVMAHFALLGIAVSQSNDRDRARHIRNVEELLLQMAEDEAVPYGEGATVGDLLAALKDL------------------
D0LTR4       LAFAHVVAATSFAAQDERAQARRALRNARVILASMPASDMVRGAQITAAELHAACRQLQRALLGERAPSPSAPGAKS
Q08XU2       YLPGLLELALLRERNGARAAAYPLMRAVHARAAKLPPDQLVEGPECLPARFYKASADAYLNQGAAE-----------
A0L8P6       HLMSHFHMGFLKIGQGRAEAGERLLRRALELANRHAQS--QTLLDPVAGLTILDVRAQLAGYLERDRVPL-------
D0LUD0       SWLAPYQLAMCLEARGELKEAEEGYRHALAVIDAGGGPGPSRSAQALAHLATTAAEVCRQRVGKRAHGNE-------
D3PC59       NFVYNLFLAFALVSKNDLKNAKYFFNMALTLVEKESS------FESLVDREVAKKIIVKYLNIL-------------
D3P9S2       SSASWFYYGLTLLKLNEKMEAKRAFEKALMFIDNNVDN-SLFYISELSGEGLKNIINYYLSQL--------------
```

Figure S4B

```
                        10        20        30        40        50        60        70        80        90       100
                         |         |         |         |         |         |         |         |         |         |
E. coli        ----------------------------------------------------------------------------------------------------
S. typhi       ----------------------------------------------------------------------------------------------------
Q98BP3         ----------------------------------------------------------------------------------------------------
Q2W002         -------------MSANSKPLNIGEAFGRAMAAWDDGNAAEARRLARSIVESKPDFGGAHYLLGLIALAQGQARRAAEHLTQAVAADPNQSVPRLALGRA
D5CN06         --------MTREISLTQAGVRTLELALLQAVELHQAGRLQEAEQCYQAILKLDPHHAKANHNLGVLSVQMGQSEAGLAYFAAALEADPAHGQYWLSYIDT
Q0F1Z8         MARKTRKKTPPPRKVIHRPPQPPQTQINAIAQRLNSGDLETAEKLAQSLARSFPGHAHSWTMLGIVLAQMGRVAESLTPLQKAVNLMPGDSGPHSNLGNA

                       110       120       130       140       150       160       170       180       190       200
                         |         |         |         |         |         |         |         |         |         |
E. coli        --------------------------------------------------------------------------MTSSLPCGQTSLLLQMTERLALSDAH
S. typhi       --------------------------------------------------------------------------MTSSLPSGQTSVLLQMTQRLALSDAH
Q98BP3         -------------------------------------------------------------------------MLFASGDHTAAAELLLGALELAPQWAM
Q2W002         LEALDNYN-----SAILHYRAILSREPDHAEANARLAELLGRLGRNKAEALEHARRAVKVAPRHPEALCTLGTLLHQTGDHAEAARMLERSLEIRPDWAV
D5CN06         LHLAGQSEEARQVLALARQSGLQGDDVDALEASLEGAGLSGAGHQPESAGTNDQLKSTQQSKSPHQQEINTLIALFTQGRYEEVMSLSQVMTRRFPQYGF
Q0F1Z8         LLQLGRLQ-----EAEACYRKAIAVNPNDPDSHYKLGNVLSSLERLPEAEVIYRRLLTLLPENDAAKLAYVQCVALMRFKSYGPFLYKTVAQALNDAWLR

                       210       220       230       240       250       260       270       280       290       300
                         |         |         |         |         |         |         |         |         |         |
E. coli        ---------------------------------------------------------------------FRRISQLIYQR--------------------
S. typhi       ---------------------------------------------------------------------FRRICQLIYQR--------------------
Q98BP3         --------------------------------------------------------------------GWFRLGEMQEAS--------------------
Q2W002         ALNNYGLVLGSLGQYERAVAVLSGAAELRRDHAGTRANLAAALRGMGRLDEARLHAERATKINSRDMAGWLELGLVRKALGMPEAAAAAFERAVTAAPDS
D5CN06         GWKILGVACKQQGKNAEALIPMQKAVELLPNDAEAHSNLGGVLQSLGRLSEAEAMLSLALRINPDNADALFNLGITLQGLDRLDEAERSYRKALQIRPDY
Q0F1Z8         PTDLVGVACN-----------------------------------------------------------LLTINPLIAPYLQQTDMP-------------

                       310       320       330       340       350       360       370       380       390       400
                         |         |         |         |         |         |         |         |         |         |
E. coli        ----------------AGIVLADHKRDMVYNRLVRRLRSLGLTDFGHYLNLLESNQHSGEWQAFINSLTTNLTAFFR--EAHHFPLLADHARRRSGEYRV
S. typhi       ----------------AGIVLADHKRDMVYNRLVRRLRALGLDDFGRYLSMLEANQNSAEWQAFINALTTNLTAFFR--EAHHFPILAEHARRRHGEYRV
Q98BP3         --------------GKLDLAAQAWTTSLKLDPADRQGAALKLQLIG--KAPVAAAPPSAFVEALFDHYAETFEEMLVGKLDYRLPEFLEQMIRKARPGRF
Q2W002         VHAHYCLAEARLVLGEKNRAAKSFRRCLELDPEDRHGAALGLAQAG--AGEAPARAPDAYVRQLFDDYADKFDAALVDKLAYRAPALLAQAMDKALDRRQ
D5CN06         AEVHGSLGAVLQATGRLQEALTHFQQRSLLAPGNPVDQHLIASLTG----ETTERAPAKYVEDVFDSYADNFDSHLLQVLKYETPRKLLESISHHPPNAD
Q0F1Z8         --------------HHQVRPINQLCEGNTLAEIGNDPLYKALLRSVPVYDENLEQWLTDARQMLLNAANATTNPLQTSWFSFFGPLAEQCFINDYLFYCS

                       410       420       430       440       450       460       470       480       490       500
                         |         |         |         |         |         |         |         |         |         |
E. coli        WSAAASTGEEPYSIAMTLADTLGTAPGRWKVFASDIDTEVLEKARS-GIYRHEELKNLTPQ-QLQRYFMRGTGPHEGLVRVRQELANYVDFAPLNLLAKQ
S. typhi       WSAAASTGEEPYSIAITLADALGMAPGRWKVFASDIDTEVLEKARS-GIYRLSELKTLSPQ-QLQRYFMRGTGPHEGLVRVRQELANYVEFSSVNLLEKQ
Q98BP3         RL-ALDLGCGTGLMGERLRPLVDRLEG------VDISARMLKKARAKGIYDTLTKADLQG--FSHSGEKPDLVTSADVLIYIGALDGLVGTVAG-LLAEG
Q2W002         DLDVLDAGCGTGLAAPVLRPLAARLDG------IDLSGAMVDKARARGLYDGLEVGELVAG-LRHHPHAYDLVVAADVLVYFGGLEDIMAATAL-ALKPG
D5CN06         KWDVLDLGCGTGLFGVEVAPFAKQLVG------VDLSSKMLEQARARKLYQRLEQADLVTMMQGEPAASFDVIVAADVFVYLGKLDDVIAEAGR-LLRPG
Q0F1Z8         ADEIQTASALKDALITTLEKEPEEVSP-LRLIAVASYFPLYSIAGSHKLLERQWPDAIQRLLIQQIKEPREEIALRPSITSLTPVENEVSLAVQAQYEES

                       510       520       530       540       550       560       570       580       590       600
                         |         |         |         |         |         |         |         |         |         |
E. coli        YTVPGPFDAIFCRN----------VMIYFDQTTQQEILRRFVPLLKPDGLLFAGHSENFSHLERRFTLRGQTVYAL------------------------
S. typhi       YNVPGPFDAIFCRN----------VMIYFDKTTQEDILRRFVPLLKPDGLLFAGHSENFSNLVREFSLRGQTVYAL------------------------
Q98BP3         GLFAFSIETLAGDG----------DFALQPSRRYAHSQPYVGRTLEAKGLAILALEPTTIRQDRREPVKGLGVVAWKPHAA-------------------
Q2W002         GVFAFTVERADDCA----------SYVLGAKNRYAHAPEYVDAVAAANGFSVVSAERASTRQEAGEDVPGLVVVLRKG----------------------
D5CN06         GILAFSVEDCDMSPRRSDAQGEVPDYQLKNTGRYGQSLGYLARLASVNGLQIQEEVATQLRIDHGKPILGHICLWAKESATNAKHGAPSADQLLQGAVRC
Q0F1Z8         PYPRWVKLPAAIEPVAIN----TYLHSLFPGVVIEPIENMMQPDLLVAGCGTGQHPIETARLIRNASVLAVDLSLASLSYAERKAREMGIQNIEFARADI
                                                        .      *            .      : .

Prim.cons.     G62AF22EA23CR22222AQGE2222ILF2KTRY2HILRY2VPLLK32GLL2AGH2E22SRL2R222V2G2TV2A2K4SAA22K2222222222222222
                       610       620       630       640       650       660       670       680       690       700
                         |         |         |         |         |         |         |         |         |         |
E. coli        ----------------------------------------------------------------------------------------------------
S. typhi       ----------------------------------------------------------------------------------------------------
Q98BP3         ----------------------------------------------------------------------------------------------------
Q2W002         ----------------------------------------------------------------------------------------------------
D5CN06         HQAGQLHDAERLYREILGIDQNHSKANHNLGSLYIQKSQPAASLPFFAAALEADPAHGQYWLSYIDALHQAGQKQVAREVLALARQYGLGGEDVDALEMN
Q0F1Z8         LKLGSLDRTFDIIESVGVLHHLDRPEEGWKVLVSMLRPNGVMRLGFYSEVARRHVVKARELIAQRNFAATADGIRKARPYLREVDRNQTLGCAVRSTDFY

                       710       720       730       740       750       760       770       780       790       800
                         |         |         |         |         |         |         |         |         |         |
E. coli        ----------------------------------------------------------------------------------------------------
S. typhi       ----------------------------------------------------------------------------------------------------
Q98BP3         ----------------------------------------------------------------------------------------------------
Q2W002         ----------------------------------------------------------------------------------------------------
D5CN06         LRGGPADMAGQSQSYCPVCGSNQVSFLPLPEFYRESSRLYGFEHFGKGEMISLEQYTCNRCGASDRERLYALWIDQQLEKNSLSRSAKVMHFAPEEALSK
Q0F1Z8         SLSACRDLLFHVQEHR------LT-LAQIQQFIDENGLTFLG---FTAEPMTIRAYHTRFPDDPTATNLENWAIYEQENPDAFFDMYQFWLQKRP-----
```

```
                    810       820       830       840       850       860       870       880       890       900
                      |         |         |         |         |         |         |         |         |         |
E. coli      ----------------------------------------------------------------------------------------------------
S. typhi     ----------------------------------------------------------------------------------------------------
Q98BP3       ----------------------------------------------------------------------------------------------------
Q2W002       ----------------------------------------------------------------------------------------------------
D5CN06       RLRQLFANYETADFGMGQVDHKVDLQNLPFADESYEFFICSHVLEHVESDDRAIRELYRITRTGGCGILVAPIIVGLERTVEDPSVKDAAGRWRLYGQDD
Q0F1Z8       ----------------------------------------------------------------------------------------------------

                    910       920       930       940       950
                      |         |         |         |         |
E. coli      --------------------------------------------------
S. typhi     --------------------------------------------------
Q98BP3       --------------------------------------------------
Q2W002       --------------------------------------------------
D5CN06       HVRLYAHDDYVNKIRSHGFHVAELGEEYFGEEIFHSLGLTHTSILYVVSK
Q0F1Z8       --------------------------------------------------
```
