# Supplementary material for: Genes Encoding Cher-TPR Fusion Proteins Are Predominantly Found in Gene Clusters Encoding Chemosensory Pathways with Alternative Cellular Functions
Source: PLoS One. 2012 Sep 20;7(9):e45810. doi: 10.1371/journal.pone.0045810 (PMC3447774; doi:10.1371/journal.pone.0045810)
Supplement: Analysis S1 — Analysis of chemoreceptor-TPR fusion proteins. Sequences were retrieved from InterPro by a search of the CheR-TPR containing genomes (Table S1) for matches of InterPro signatures IPR011990 (TPR-like helical) and IPR004089 (methylaccepting chemotaxis protein [MCP] signaling domain). Shown are protein sequences and the fragment recognized by IPR011990 is shown in red. The transmembrane regions as predicted by the TMHMM Server v. 2.0 (http://www.cbs.dtu.dk/services/TMHMM-2.0/) are shaded in yellow. The sequence fragment predicted to be located in the cytosol is underlined. Shown are also images from the InterPro output of each sequence. The sequence fragments shaded in red containing 5 additional amino acids at each side were submitted to the three dimensional homology modeling server CPHmodels 3.2 (Nielsen et al. (2010) CPHmodels-3.0 Nucleic Acids Research 38, doi:10.1093/nar/gkq535). Shown are the resulting homology models and the protein database ID of the templates used for modeling. In each case the model showed the typical structure of a TPR domain containing 2–3 TPR. For reference, the three dimensional structure of the TPR domain of pdb entry 2C2L is shown below. This template was used to generate some of the homology models. At the end of this document a sequence alignment of members of the chemoreceptor-TPR family is shown. The alignment was made using the CLUSTALW algorithm of the NPSA server (http://npsa-pbil.ibcp.fr/cgi-bin/npsa_automat.pl?page=/NPSA/npsa_clustalw.html). The GONNET matrix was used and the gap opening and gap extension penalties were set at 10 and 0.1, respectively. The TPR regions are shaded in yellow. Amino acids in red are fully conserved, those in green strongly similar and those in blue weakly similar. (DOCX) [file pone.0045810.s009.docx]

# Analysis S1

**Arthrospira maxima CS-328 9, protein** [**B5W0C4**](http://www.ebi.ac.uk/interpro/ISpy?ac=B5W0C4)

>tr|B5W0C4|B5W0C4_SPIMA Methyl-accepting chemotaxis sensory transducer OS=Arthrospira maxima CS-328 GN=AmaxDRAFT_2218 PE=4 SV=1

MTASAEYLQEYQKTEAAYTDGKLDEAATLVYKLVEDYPEDPFARLLCGHIYYGLQQYDVAREQYEVVLSLTEDPALVEQAEDYLTEASQFCEDSETGSSLGDISLDDEFDDEFDDAQETILEDLSSVGDDANGSLDFDLEEELDLNAIDEQLDELEELTAASQQLGKYPQKPSATIETLDDALDLYDDEDMGMEEIDISSALDDLDELSNLEFEEGTEENLEAIDDDLGDLDRSADELEPENLNGKASVGGEELNDSLEDEFDLEEDNFNPLELEANPLVDDSNFDLDDEVDPDLTNPLNNPFAQGQETDEEEEDPDLNFDLETSDPFVMDEEDELVLTEGLDPEDPMSGSLEQLSEDLFEEDLIDDSHKDSPGTNTPGVTQEHQDSLDDLDDDFSLTDLPLTDGPITNEPIVDNVAEEIKPPDLGDVQDELDIGELELEEDDIFSPLEDMADARHQTVAETNGNGNGKGLVKPDQISLNKTPNNNLDEEEENFSDFNLDGDKINDSVSEELELDDIPDTFDLDSLEESTVSNGTANGAIAHNKTNQTSGSQVNEFLEDFEEFDDVEGFGIPDAAGYAFMPDSADLDDDDLDSNFLGRSSSIPDNDSSAIYEDDVFNTPTEREAITAFSNLSEDSVDTNISVEQGSFAFLENKPLRSKSFYIALGSGLVTLIAVAVATNIATKVAASSYQGEVVNYLRRSGWLMTIVAGASSFGTAFAMGRITSQQLEKATGDLQKQFDAIARGNLNARVNVYAEDELGQMCAKFNYMAQFIESTTREAQRKAEEQEEAKENLQRQVIRLLDDVEGAARGDLTVSAEVTADVLGAVADSFNLTIQNLREIVVQVKQAARQVSRGATDSASFAKDVAGDALRQAEELAATLNSVQLLTDAIQRVADSAKEAEEVARTAAAVATKGGEAVEMTVAGILKIRETVAETTRDVKRLAESSQEISKIVAIISNIASRTNLLALNASIEAARAGEAGRGFAIVADEVRQLADKSAKSLKEIEQIVMQIQSQTSSVMMAMEEGNQQVIEGTRLAEQAKRSLDDIIQVTNRIDVLVRSITADTVEQNETARAVAEVMQAVELSAQDTSQEAQRVASALSNLVGVARDLLTSVERFRVDPSEH

InterPro output


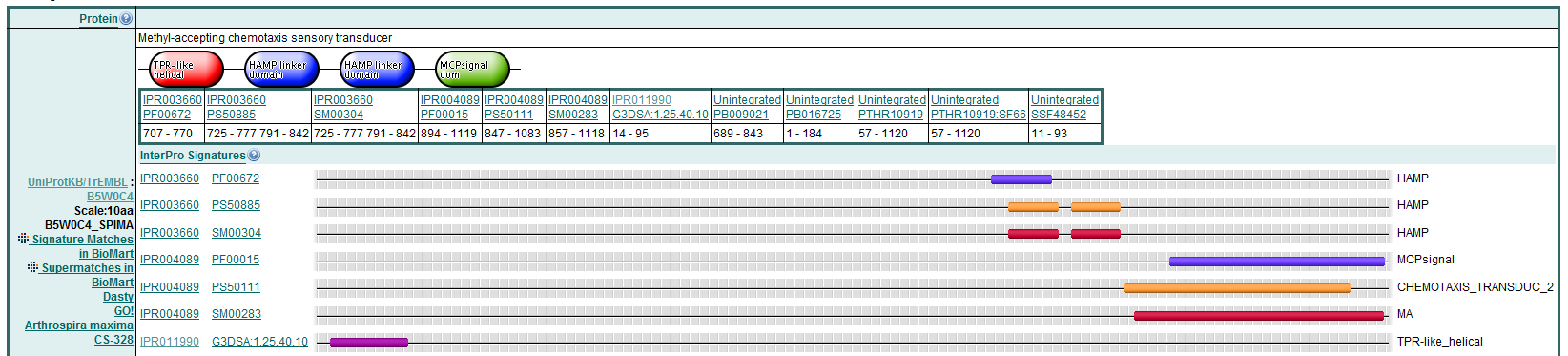


**3D model of TPR region using template pdb ID 2C2L**: Zhang et al. (2005) Chaperoned ubiquitylation--crystal structures of the CHIP U box E3 ubiquitin ligase and a CHIP-Ubc13-Uev1a complex. [Mol Cell.](http://www.ncbi.nlm.nih.gov/pubmed/16307917?dopt=Abstract) 20:525-38.


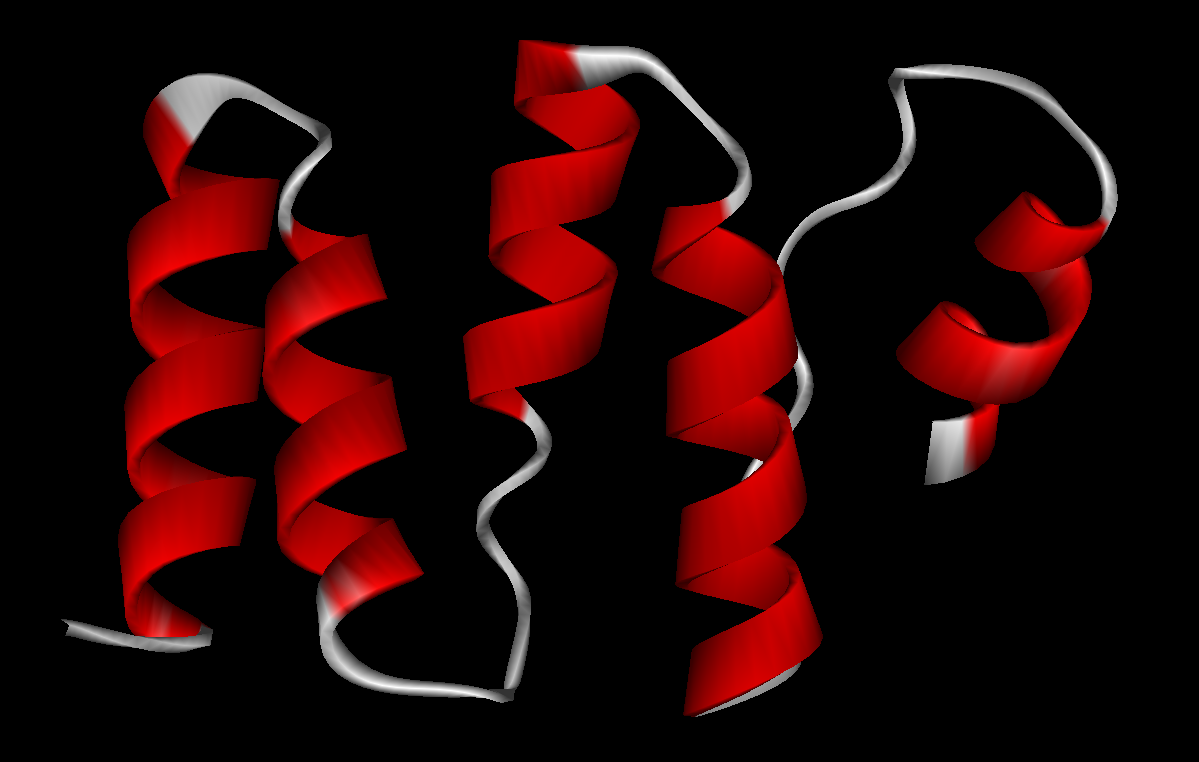


**Arthrospira platensis NIES-39 92 protein**  [A0YZI7](http://www.ebi.ac.uk/interpro/ISpy?ac=D4ZR05)

>tr|D4ZR05|D4ZR05_SPIPL Putative methyl-accepting chemotaxis protein OS=Arthrospira platensis NIES-39 GN=NIES39_E01060 PE=4 SV=1

MTASAEYLQEYQKTEAAYTDGKLDEAATLVYKLVEDYPEDPFARLLCGHIYYGLQQYDVAREQYEVVLGLTDDPELVQQAEDYLTEASQFCEDSETGSSLGDISLDDEFDDAQETILEDLSSVGDDANGSLDLDLEEELDLNAIDEQLDELEELTAASQQLGKYPQKPSATIETLDDALDLYDDEDMGMEEIDISSALDDLDELSNLEFEEGTEENLEAIDDVLGDFDISADELEPENLNGKASVGGEELNDSLEDEFALEEDNFNPLELEANPLVDDSNFDLDDEVDPDLTNPLNNPFAQGQETDEEEEDPDLNFDLETSDPFVMDEEDELVLTEGLDPEDPMSGSLEQLPEELFEEDLIDAPHKDSPATNTPGVTQEHQDSWDDLGDDFSLTDLPLTDEPITNETIVDHVALEIKPPDLGDVQDELDIGELELEEDDIFSPLEDMADARHQTVAETNGNGNGKGLVKPDQISLNKTPNNNLDEEEENFSDLNLDEFDQVDDSVSDEFELDDIPDTFDLDSLEESTVSNGTANGAIAHNKTNQTSGSQVNEFLEDFEEFDDVEGFGIPDAAGYAFMPDSADLDDDDLDSNFLGRSSSIPDNDSSAIYEDDVFNTPTEREAITAFSNLSEDSVDTNISVEQGSFAFLENKPLRSKSFYIALGSGLVTLIAVAVATNIATKVAASSYQGEVVNYLRRSGWLMTIVAGASSFGTAFAMGRITSQQLEKATGDLQKQFDAIARGNLNARVNVYAEDELGQMCAKFNYMAQFIESTTREAQRKA

EEQEEAKENLQRQVIRLLDDVEGAARGDLTVSAEVTADVLGAVADSFNLTIQNLREIVVQVKQAARQVSRGATDSASFAKDVAGDALRQAEELAATLNSVQLLTDAIQRVADSAKEAEEVARTAAAVATKGGEAVEMTVAGILKIRETVAETTRDVKRLAESSQEISKIVAIISNIASRTNLLALNASIEAARAGEAGRGFAIVADEVRQLADKSAKSLKEIEQIVMQIQSQTSSVMMAMEEGNQQVIEGTRLAEQAKRSLDDIIQVTNRIDVLVRSITADTVEQNETARAVAEVMQAVELSAQDTSQEAQRVASALSNLVGVARDLLTSVERFRVDPSER

InterPro output


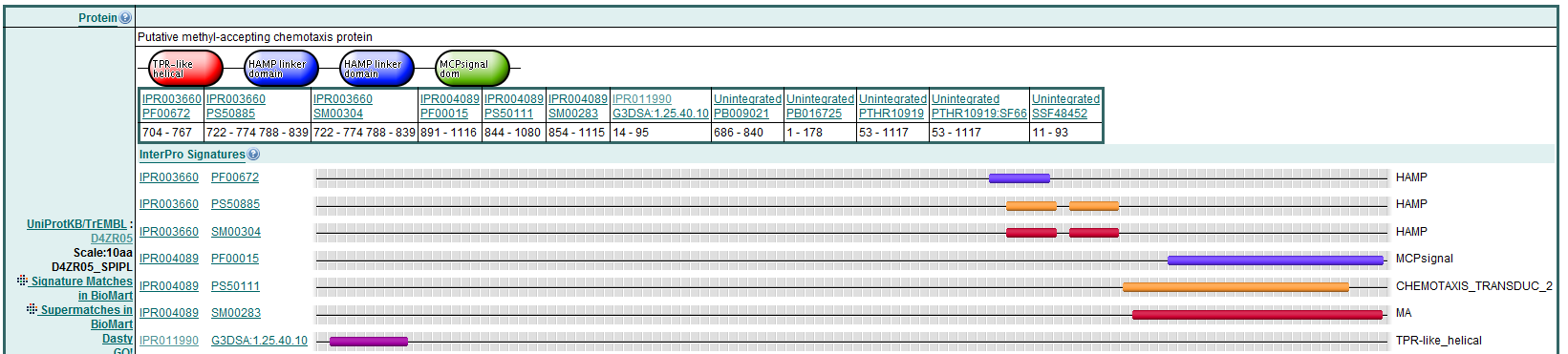


**3D model of TPR region using template pdb ID 2C2L**: Zhang et al. (2005) Chaperoned ubiquitylation--crystal structures of the CHIP U box E3 ubiquitin ligase and a CHIP-Ubc13-Uev1a complex. [Mol Cell.](http://www.ncbi.nlm.nih.gov/pubmed/16307917?dopt=Abstract) 20:525-38.


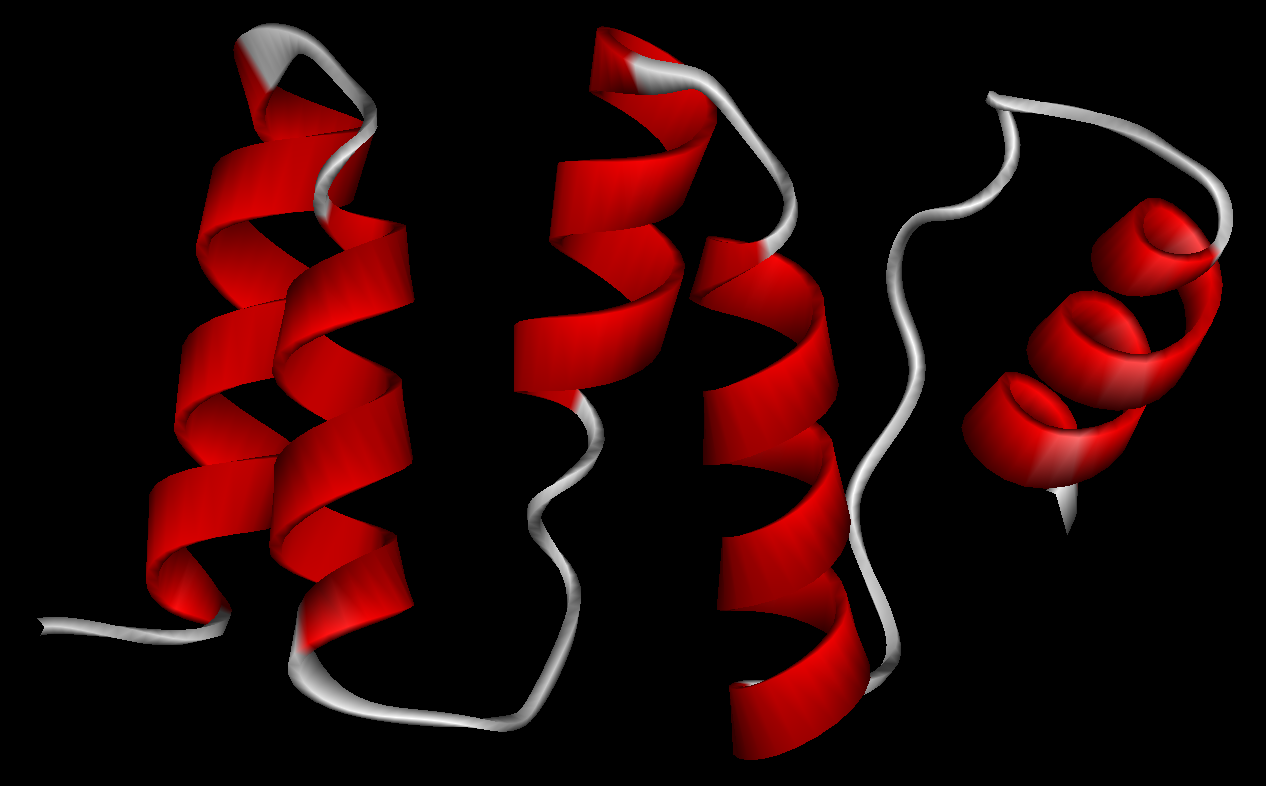


[Nostoc punctiforme (strain ATCC 29133 / PCC 73102)](http://www.uniprot.org/taxonomy/63737)  [**B2ITW6**](http://www.ebi.ac.uk/interpro/ISpy?ac=B2ITW6)

>tr|B2ITW6|B2ITW6_NOSP7 Methyl-accepting chemotaxis sensory transducer OS=Nostoc punctiforme (strain ATCC 29133 / PCC 73102) GN=Npun_F5963 PE=4 SV=1

MKLEEEMAASIDNYEPTYQQAMTAYVQRNYEVAATLVDQVVQNLPNDPNTHLLRGHIYYVLQQYDVAKEEYQQVLSLTNEQEIIGFANNGIENINQYLQSFGGQIDTSGSQEQIHSSEMPDPLAYSEPELEDLDPSEEFDSNNLDLNFFGEHQETVDEVKELSSNSPFDIPTEDSIGMGKIPDSSITFGDDPFALDEESEEQDFNSSSYEEKTELDLPAFWQEDMSEEIHEESLVNSHFSENEINHDRGNSAIDNNNSSSSNSQTSSSKNNFSDLLIEPKSPEPKIGNLEYNKSNFGGETLLIVVEEEPINSSATTNNNSRYDLPETEAHDWLKSKDLEVEQEFQTESSDNYSSFKSDIPLEEKQMKSSEIISKNSFDDENFDMEAFESAFGSDGLSSYEDSSNILNGENSKSNIDFLDDFEEFDDLGNIPGFDLIEEDSNFGDAAMYSAPAEASGSGRSQSTDTSGSNAADREEELFSMTGSHEGVPVFSQTDVSKLEPNVSVEQGWLAPLENASIERKQWLIAGSVGIVSALVVATVSFVATTFSPVQQRESVRNTGWAMSLAAGIAGFATAGFMGNLTLKQIRRTTNDLQAQFEAVRQGNLNAQATVFSEDELGHLSTGFNEMARVIFTTTSEAQRKADEQEEAKENLQRQVIRLLDDVEGAARGDLTVQAEVTADVLGAVADAFNLTIQNLRDIVQQVKVAAKDVTKGATNSETFARALSSDALRQAEELAVTLNSVQVMTDSIQRVAEAAREAETVARDASTIALKGGEAVENTV

AGILEIRETVAETTRKVKRLAESSQEISKIVALISQIASRTNLLALNASIEAARAGEAGRGFAIVADEVRQLADKSAKSLKEIEQIVMQIQSETGSVMTAMEEGTQQVIKGTKLAEEAKRSLENIIQVANRIDILVRSITSDTVEQTETSRAVAHVMQSVELTAQETSQEAQRVSGALQHLVGVSRDLIASVERFRVETMETR

InterPro output


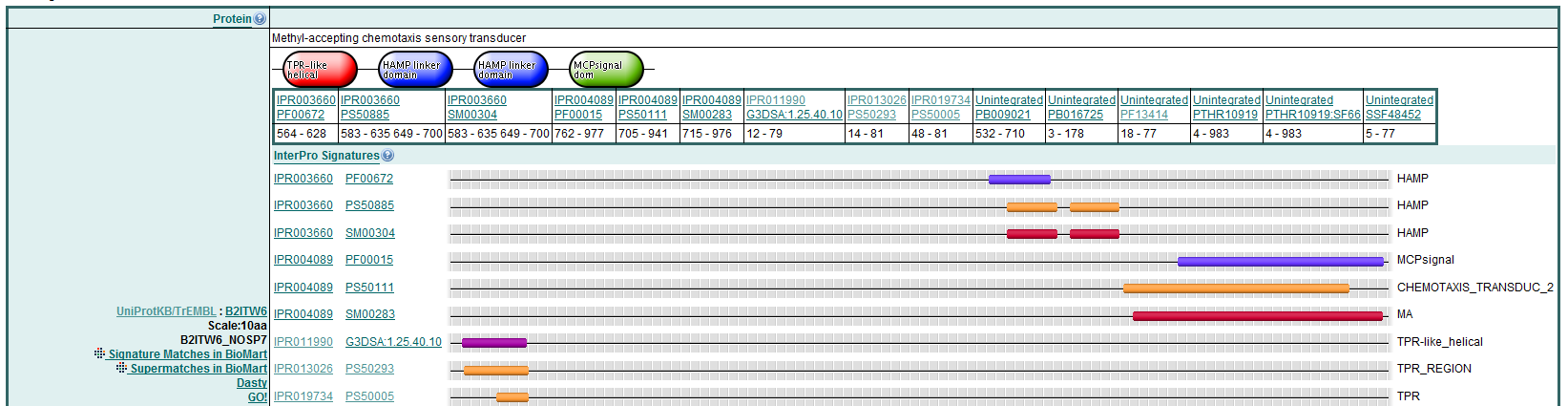


# 3D model of TPR region using template pdb 3FP3: Li et al. (2009) Molecular chaperone Hsp70/Hsp90 prepares the mitochondrial outer membrane translocon receptor Tom71 for preprotein loading. [J Biol Chem.](http://www.ncbi.nlm.nih.gov/pubmed/19581297?dopt=Abstract) 284:23852-9.


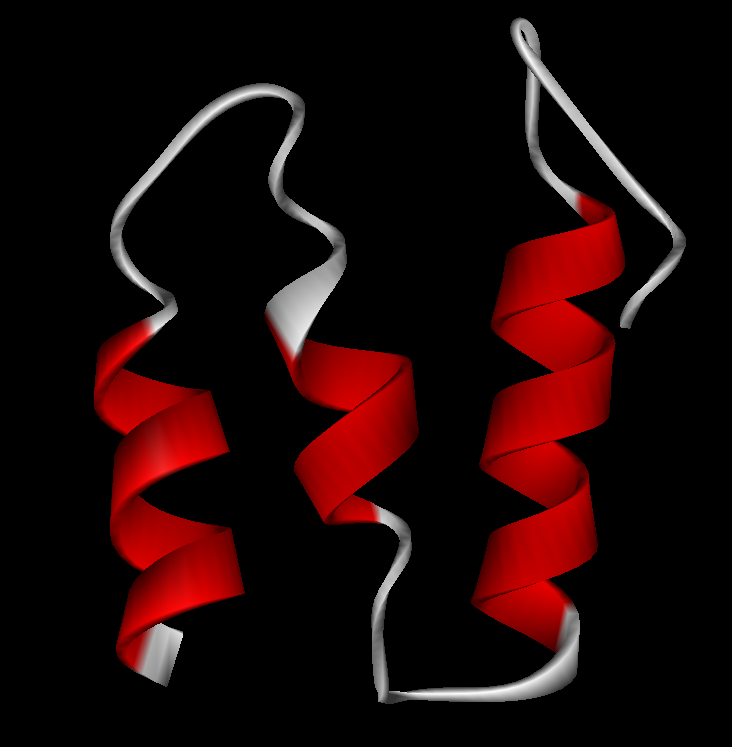


[Cyanothece sp. CCY0110](http://www.uniprot.org/taxonomy/391612) [**A3IRC0**](http://www.ebi.ac.uk/interpro/ISpy?ac=A3IRC0)

>tr|A3IRC0|A3IRC0_9CHRO Methyl-accepting chemotaxis protein OS=Cyanothece sp. CCY0110 GN=CY0110_21080 PE=4 SV=1

MASGTDYAKLYNEAYIAYGQGNYEEAATLIEPMAGAFPEDPDVLLLQGHIYFSLGRYETACQQYQSVINRADRPDLVECANQGLQQARQFLHQSGNNHKTDDNLQQPMINSESMKIEQQWSDSGEVSGDWQGEEFAVDGLDWDSGIFDDDDLGEPTIGHQGNASQSSEEQPEKQNSFEMPVDPFTTLSEVRPFDSQTDIPNTNASEEEGWTDFVDEDFPDFSFGDEQGLETEAHTGAGDEENTFVVSSSLNPPSLSNRGSTPPLQDSSHDWSNSPAQDDPTVHATPQMMSSDQVQEHNRVLDEFDAFQEEALEDLSNFDFTEMSDQLPDSDLFTQASEDGSGSIGLQTGDLLSEPGPQTVNWVKSDDMANTSLSDSEVKIVKPTAERQQGYLRWFFNAPLKIKPWMAAGSTGLVSFMAILLVSSFAWLVSPKPEATEVGEEEATTEQVEQSDTTETSSEETEENEKPAKSGNPILLLALTGGLVGFGGTLFFGLISSYQLKRTIDDLQTQFDSMYAGDYNVKATIYSEDELGQLSHSFNQLSRVILTTTSEAQKRAAEMEQAREELQRQVIRLLDDVEGAARGDLTVEAEVTADVLGAVADAFNLTIHNLREIVAQVKRTAKQVNKSSTDSELFARNNSRDALRMAEELAVTLNSVQVMTESIQRVAENAREAEEVAHTSSVTALKGGEAVERTVAGILQIRETVSETTRKVKRLAEASQEISKIVALISQIASRTNLLALNASIQAARAGEAGRGFAIVADEVRQLADRSAKSLKEIEQIVLQIQSETGSVMTAMEEGIQQVIDVTDKSEQAKRSLEDIIDVSNHINTLVRSITGDTVQQQDNSKAVSQVMQSVELTAQETSQESQRVAGALQNLVGISRDLLTSVERFHIEEAEKS

InterPro output


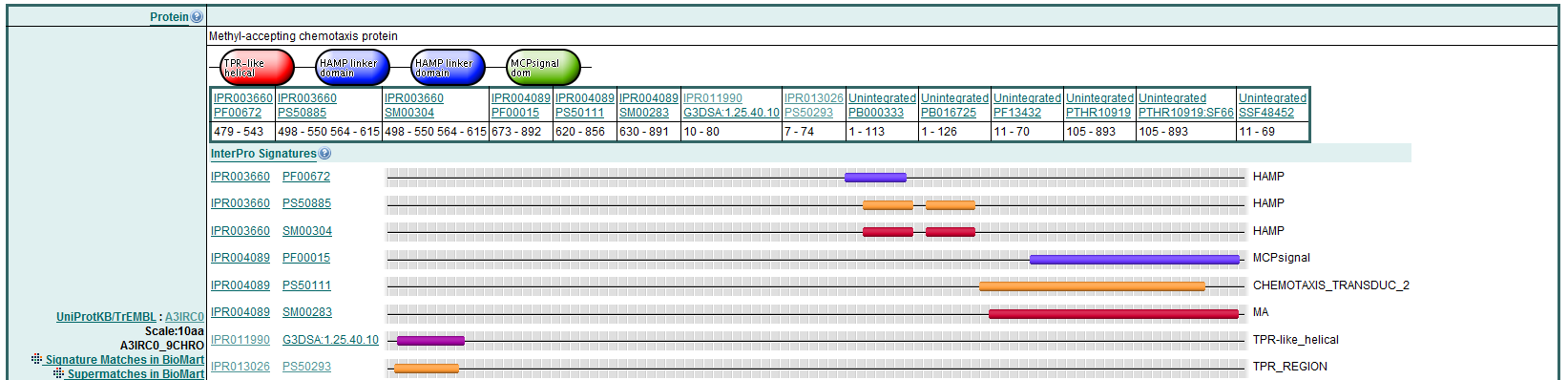


**3D model of TPR region using template pdb 2AVP**: [Kajander et al.](http://www.ncbi.nlm.nih.gov/pubmed?term=%22Kajander%20T%22%5BAuthor%5D) (2007) Structure and stability of designed TPR protein superhelices: unusual crystal packing and implications for natural TPR proteins. [Acta Crystallogr D Biol Crystallogr](http://www.ncbi.nlm.nih.gov/pubmed/17582171?dopt=Abstract) 63:800-11.


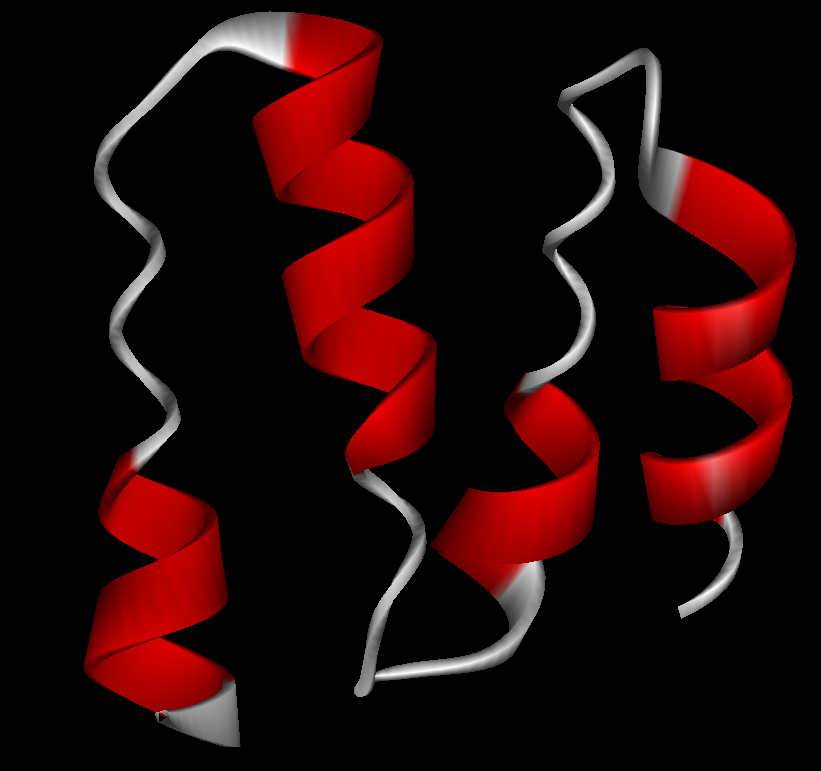


[Cyanothece sp. (strain PCC 8801)](http://www.uniprot.org/taxonomy/41431) [**B7JXP7**](http://www.ebi.ac.uk/interpro/ISpy?ac=B7JXP7)

>tr|B7JXP7|B7JXP7_CYAP8 Methyl-accepting chemotaxis sensory transducer OS=Cyanothece sp. (strain PCC 8801) GN=PCC8801_0720 PE=4 SV=1

MAPGTDYSKLYNQAYVAYGQGEYEEAATLIEPMAEAYSQDPHVLLLRGHIHLSLQQYETACADYQSVLTLTDQQDLLKLAQQGLAQAQEMLGQASPNNLNHSQKSASYLGSSSLNDEDEDEEDGLIWQDNEEGEADWDSNFQIDENLDWDFDEEDFGEPTIGHDPSQANPFDNAGQTTFRPPQADVASLAVEDDLNLETPDTWQSPFTEAMEDFPFTDDEDLRTEPHPTASVGKNTFLVTPKLDNSRENPEGTPIESFLRPSSRNLLNYNSDEDLTLPPSSSSMGNQENQLVENLDSDLSHLTEMAAELPDSDIFTRSAEVFSDNLSLDASDLMSAAPTPTTVTWVKPNEAMGGDLSVASNQGIKPTVEVAQQGWAAWFINASLKHKPWIAAGATGIASFLMVLLVSTFTSVMSPKPQSGTTPVPQTSQSTPKPQNNQKPAAKATNKPSAAKSGSPVLLMALLGGLTGFSTTLLFSFLICGQVKRTIEDLQAQFDAMYAGDFNVKATIHSSDELGQLSLSFNQLARIILTTTSEAQQRAAETEQAQEDLTRQVIRLLDDVEGAARGDLTVEAVVTADVLGAVADAFNLTIQNLREIVGQVKQAAKQVNKGSTDSESFARNNSSDALRMAEELAVTLNSVQVMTESIQRVAENAREAEEVASVSSVTALKGGEAVERTVAGILGIRETVSDTTRKVKRLAEASQEISKIVALISQIASRTNLLALNASIQAARAGEAGRGFAIVADEVRQLADRSAKSLKEIEQIVLQIQSETGSVMTAMEEGIQQVINLVDTSEQAKRSLEDIIDVSNRINGLVRSITADTVKQRENSRAVTQVMQSVELTAQEASQESQRVAGSLQTLVAISRDLLASVERFRVDKSET

InterPro output


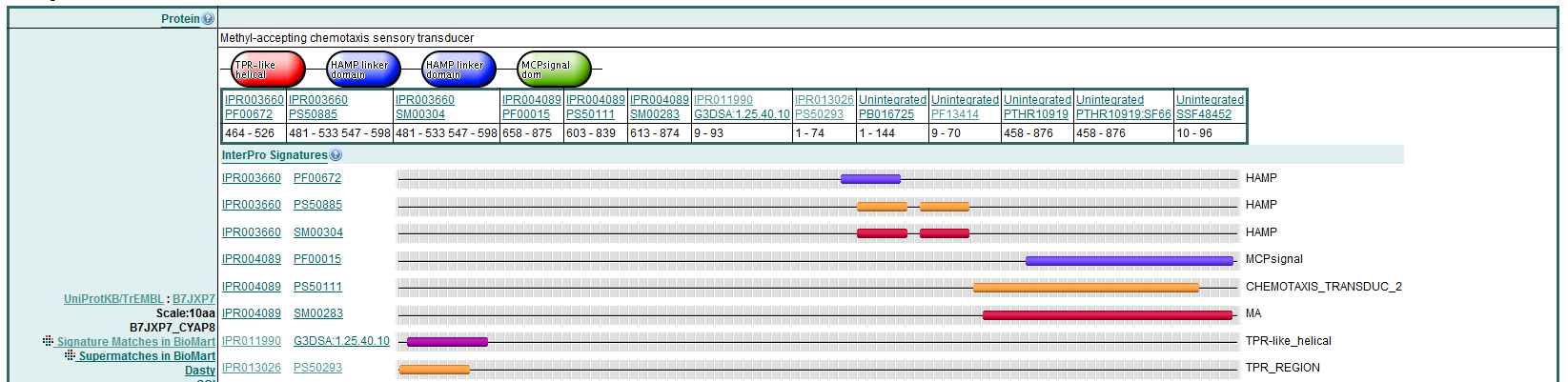


# 3D model of TPR region using template pdb 1NA3: Main et al. (2003) Design of stable alpha-helical arrays from an idealized TPR motif. [Structure](http://www.ncbi.nlm.nih.gov/pubmed/12737816?dopt=Abstract) 11:497-508.


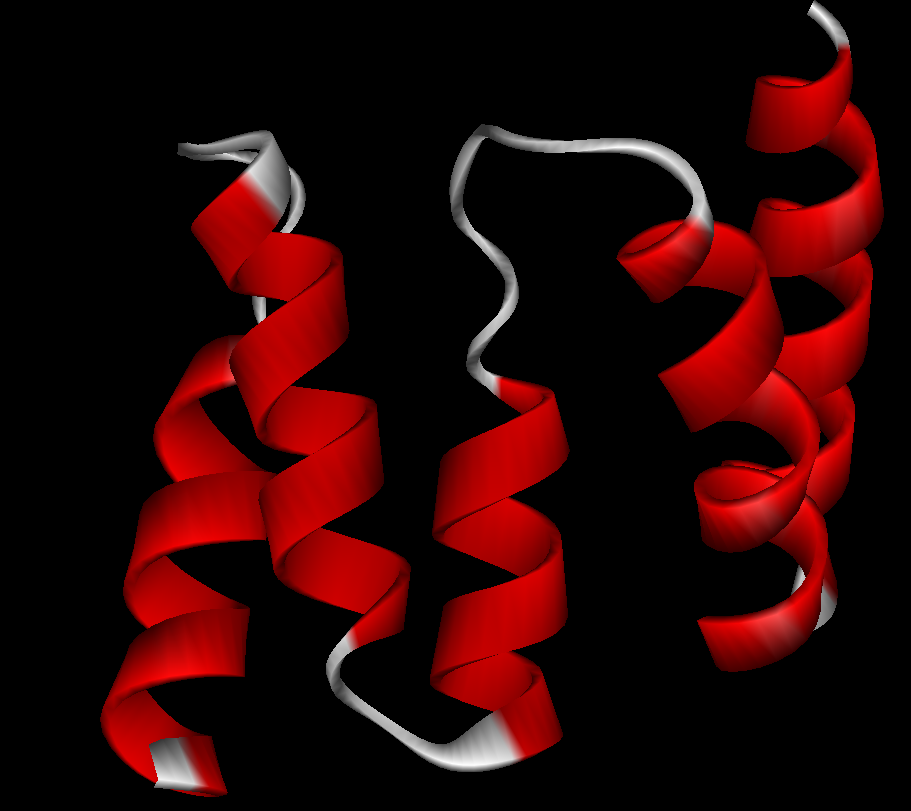


[Cyanothece sp. (strain PCC 8802)](http://www.uniprot.org/taxonomy/395962) [**C7QXD9**](http://www.ebi.ac.uk/interpro/ISpy?ac=C7QXD9)

>tr|C7QXD9|C7QXD9_CYAP0 Methyl-accepting chemotaxis sensory transducer OS=Cyanothece sp. (strain PCC 8802) GN=Cyan8802_0749 PE=4 SV=1

MAPGTDYSKLYNQAYVAYGQGEYEEAATLIEPMAEAYSQDPHVLLLRGHIHLSLQQYETACADYQSVLTLTDQQDLLKLAQQGLAQAQEMLGQASPNNLNHSQKSASYLGSSSLNDEDEDEEDGLIWQDNEEGEADWDSNFQIDENLDWDFDEEDFGEPTIGHDPSQANPFDNAGQTTFRPPQADVASLAVEDDLNLETPDTWQSPFTEAMEDFPFTDDEDLRTEPHPTASVGKNTFLVTPKLDNSRENPEGTPIESFLRPSSRNLLNYNSDEDLTLPPSSSSMGNQENQLVENLDSDLSHLTEMAAELPDSDIFTRSAEVFSDNLSLDASDLMSAAPTPTTVTWVKPNEAMGGDLSVASNQGIKPTVEVAQQGWAAWFINASLKHKPWIAAGATGIASFLMVLLVSTFTSVMSPKPQSGTTPVPQTSQSTPKPQNNQKPAAKATNKPSAAKSGSPVLLMALLGGLTGFSTTLLFSFLICGQVKRTIEDLQAQFDAMYAGDFNVKATIHSSDELGQLSLSFNQLARIILTTTSEAQQRAAETEQAQEDLTRQVIRLLDDVEGAARGDLTVEAVVTADVLGAVADAFNLTIQNLREIVGQVKQAAKQVNKGSTDSESFARNNSSDALRMAEELAVTLNSVQVMTESIQRVAENAREAEEVASVSSVTALKGGEAVERTVAGILGIRETVSDTTRKVKRLAEASQEISKIVALISQIASRTNLLALNASIQAARAGEAGRGFAIVADEVRQLADRSAKSLKEIEQIVLQIQSETGSVMTAMEEGIQQVINLVDTSEQAKRSLEDIIDVSNRINGLVRSITADTVKQRENSRAVTQVMQSVELTAQEASQESQRVAGSLQTLVAISRDLLASVERFRVDKSET

InterPro output


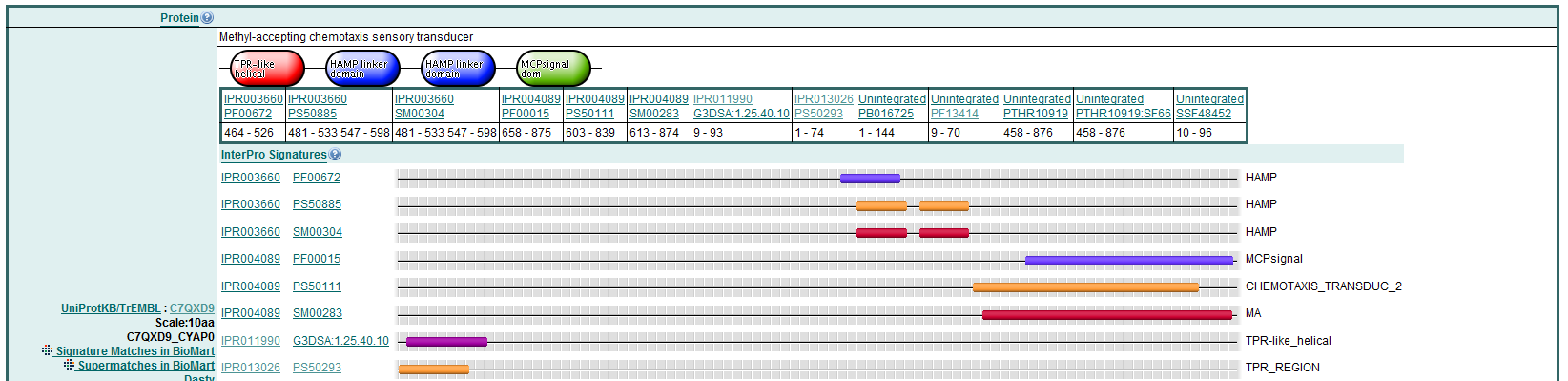


**3D model of TPR region using template pdb 2AVP**: [Kajander et al.](http://www.ncbi.nlm.nih.gov/pubmed?term=%22Kajander%20T%22%5BAuthor%5D) (2007) Structure and stability of designed TPR protein superhelices: unusual crystal packing and implications for natural TPR proteins. [Acta Crystallogr D Biol Crystallogr](http://www.ncbi.nlm.nih.gov/pubmed/17582171?dopt=Abstract)63:800-11.


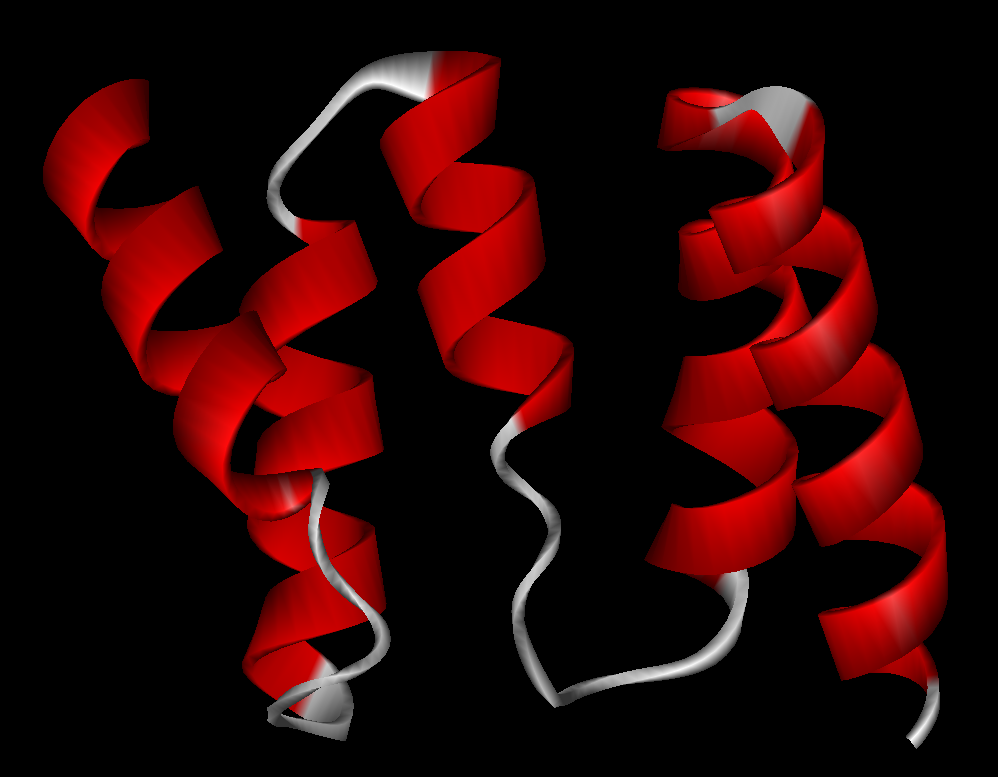


[Cyanothece sp. (strain PCC 7822)](http://www.uniprot.org/taxonomy/497965) [**E0UEC6**](http://www.ebi.ac.uk/interpro/ISpy?ac=E0UEC6)

>tr|E0UEC6|E0UEC6_CYAP2 Methyl-accepting chemotaxis sensory transducer OS=Cyanothece sp. (strain PCC 7822) GN=Cyan7822_3423 PE=4 SV=1

MTSGIDYVKQYGKANAAYCRGDLDEAVSLIDQLAQEYPDDPHTLLLRGHIFLSMQQYQIAQQEYEKVLKITQQPDLLDYAERGLEQIQLVESDFDEMDFTIDETEGFTVSQANLNTDESFEQPDFSHWSQHPDMPDLEEMDWGNQPTVHWNPDDDDMGEPTIGKEFSRHQFANPFAEDAETSFNPDLSQFEDLAETFGLPSEPSHHPELERYVNSLDQPGADDDQTNFYWHNNADEDEQRHLSFIEEDNNGFNSNGYRSNFDQFSDTGESTLVVSSDLPSNALKSNATKFLGKNPSNGKFSENLAADHSDSWNEEAFRKEPQQRKPLSSHTQQPANSDGFLNDLEVFNDDDLDGLSQFDITDVAQSLPDSGLFERHTDGLDSGLETGSGISAAAMEASSISKINWTDVGENSSANLTGASTRFIKPTVEIEQGKFSALKNLPLKRKQWITAGIAGLTSVATIFLISSAIWMFSPKQPSKKADSPANKTESVSSNPKKSDPKTDKKTTPKKNSSSSTALQAQQTAAISPFSPSMLLMMLLTGGATFGATLFFARLSTNQIKQTVDDLQTQFDAIYAGDFNVKATVYSEDELGQLSARFNQMAQAILTTTSEAQRRAAETEQQKEDLQRQVIRLLDDVEGAARGDLTVEAEVTADVLGAVADAFNLTIQNLREIVRQVKKAAEQVNKGSTDSELFARNQSSDALRMAEELAVTLNSVQMMTDSIQRVAENAREAEEVARTSSVTALKGGESVERTVAGILQIRETVSETARKVKRLAEASQEINKIVAVVSQIASRTNLLALNASIQAARAGEAGRGFAIVADEVRQLADRSAKSLKEIEQIVLQIQSETGSVMTAMEEGIQQVIDVTERSEQAKRSLEDIIQVSNRIDSLVRSITGDTVKQRENSREVAQVMQSVELTAQETSQESQRVAGSLQTLVKISRDLLESVERFKVDKNDYK

InterPro output


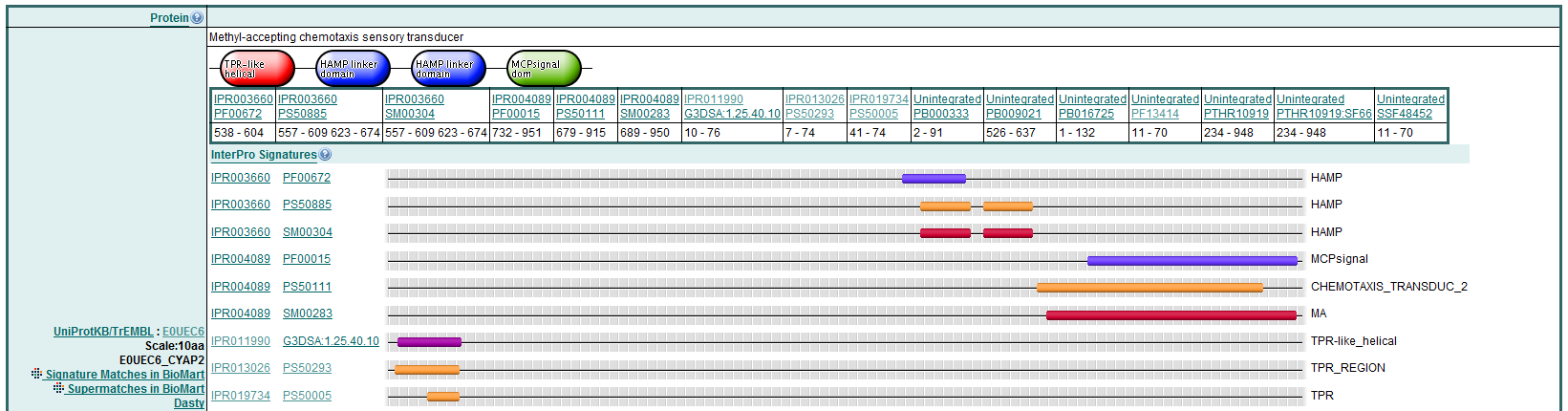


**3D model of TPR region using template pdb 2AVP**: [Kajander et al.](http://www.ncbi.nlm.nih.gov/pubmed?term=%22Kajander%20T%22%5BAuthor%5D) (2007) Structure and stability of designed TPR protein superhelices: unusual crystal packing and implications for natural TPR proteins. [Acta Crystallogr D Biol Crystallogr](http://www.ncbi.nlm.nih.gov/pubmed/17582171?dopt=Abstract)63:800-11.


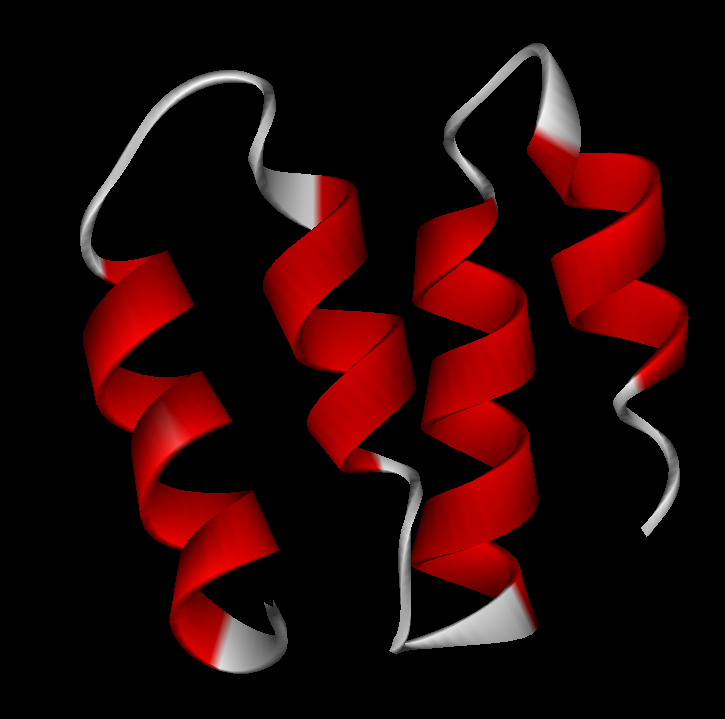


[Oscillatoria sp. PCC 6506](http://www.uniprot.org/taxonomy/272129)[**D8FZE5**](http://www.ebi.ac.uk/interpro/ISpy?ac=D8FZE5)

>tr|D8FZE5|D8FZE5_9CYAN Methyl-accepting chemotaxis protein OS=Oscillatoria sp. PCC 6506 GN=OSCI_2400001 PE=4 SV=1

MATTTDFEQEYQRAEAAYLEGNYEEAAALVYQLVEDFPEDPSARLLCGHIYCYGLQYDVAREQYMVVLSLTDDTGFVDHANNGIAYAEQFAVGAPGESLENFSEDLDLEMYPGLSGENDLEQPDLSLFGGGSDLESDLGTSSLELDNAGMMGMGLSELDFENNLENYANDAELLNPFGESPNFDSNSSGSGGMAAFADADLDSGVELGGLDFDDDAMAMADTQLTFPAPKEKPSPPAQEQRQADVFAGAASKQNGRTIGSQSLADSGLADSGIEDLGEFPEDFDDMFAPLELPDDLNFEESYPPAETNGKQKMPLQPQPTAEPGQTRSRTSGIQAKEGSDLGGSKSNYSVAEDETLLMGSHSAPEALSEIGKPSKDLNSGFKSTTQGSKAAKGEDLQNGFNIPDSFDLDSFDDDAFADSFSSYEETPSHSFGMAGPDSDFPLTPSPQTDRGDWIDDFDEFGDVGNLPDFDISDDANSSILTTGGSSGFGGMTGGVMGATTSNLDFGDNSDGSAIRDDEIFSISGTPESVPSFTPTEEPIDATVTVEQGGLAFLENAPLITKQLYTAIGTGLISLVAVALVTNFASYQALKQDKPEAIAYLRQTGWAMTAAAGFTSFLTAWGLGHLVARQVSKSTDDLQVQFDSVSQGNLEARATVYSEDEFGKMSAKFNHMAKVILTTTSDARRKADEQEQAKEDLQRQVIRLLDDVEGAARGDLTVQAEVTADVLGAVADSFNLTIQNLREIVHQVKQAARDVSTGASESATFAQGLSSDALRQAEELAATLNSVQVLTDAIQRVAESAREAEEVARGAAASAIKGGEAVERTVAGILEIRETVAETTRKVKRLAEASQEISKIVALIATIASRTNLLALNASIEAARAGEAGRGFAIVADEVRQLADRSAKSLKEIEQIVMQIQSETGAVMTAMEEGTQQVIEGTRLAEQAKRSLEDIIQVTNRIDVLVRSITADTIEQNQTASAVSHVMQAVELTAQETSQESQRVYGSLQNLVGVARDLLTSVERFRVETAERQQ

InterPro output


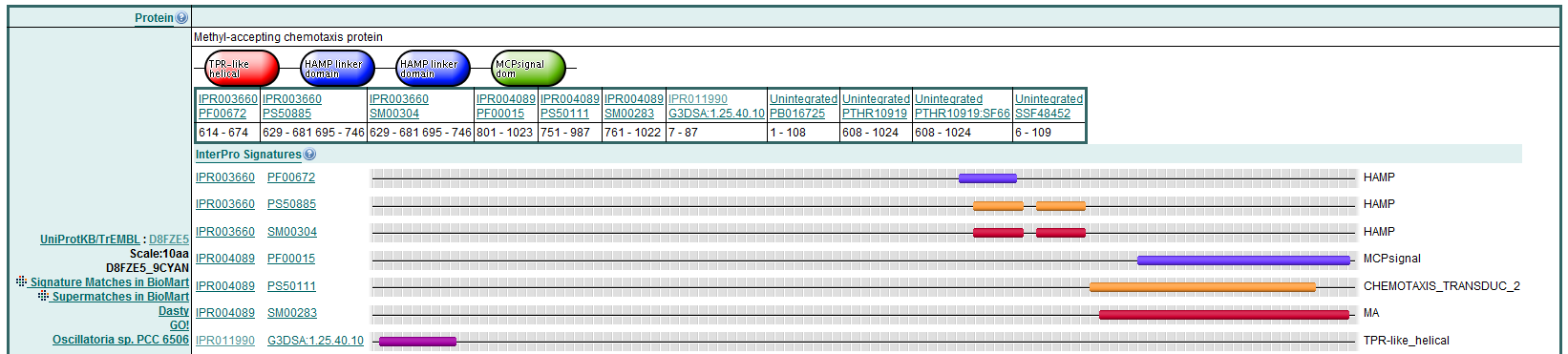


# 3D model of TPR region using template pdb 1NA3: Main et al. (2003) Design of stable alpha-helical arrays from an idealized TPR motif. [Structure](http://www.ncbi.nlm.nih.gov/pubmed/12737816?dopt=Abstract) 11:497-508.


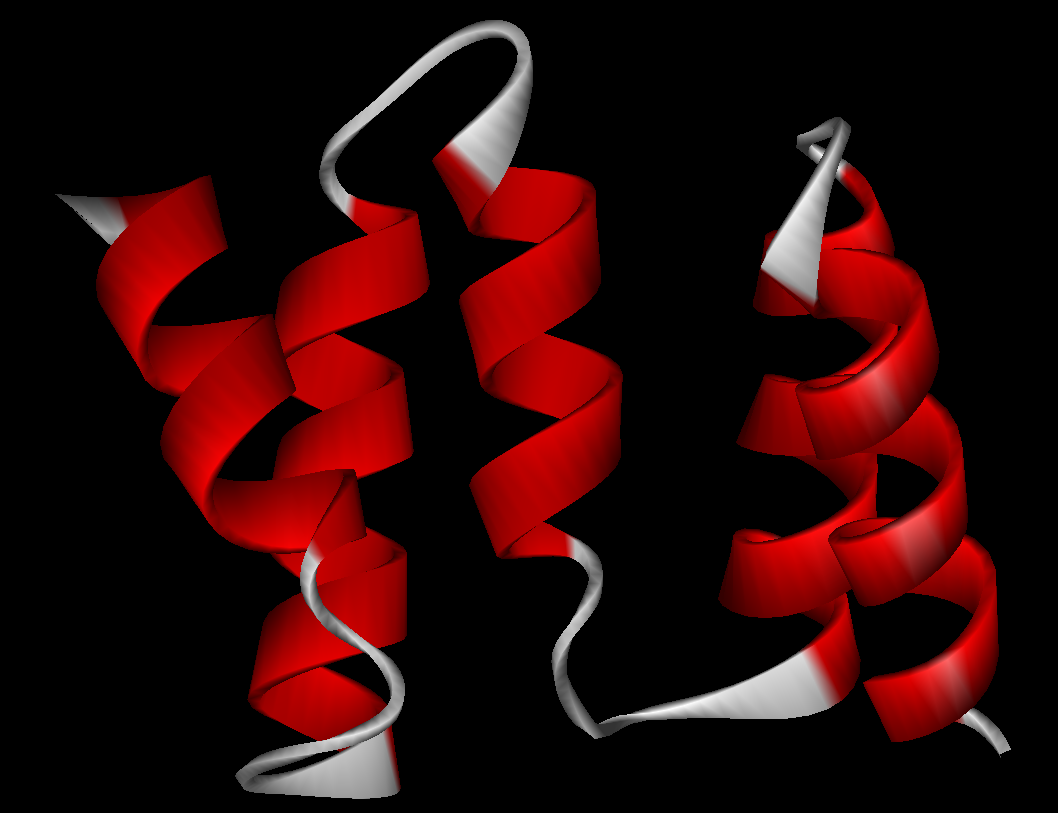


Lyngbya sp. (strain PCC 8106) (Lyngbya aestuarii (strain CCY9616) **[A0YZI7](http://www.ebi.ac.uk/interpro/ISpy?ac=A0YZI7)**

>tr|A0YZI7|A0YZI7_LYNSP Chemotaxis sensory transducer OS=Lyngbya sp. (strain PCC 8106) GN=L8106_12305 PE=4 SV=1

MQASTDFLQEYQQTEAAYNQGNYEEAAALVYQLVENYPEDPSARLLCGYIYGYGLQQFEVAREQYFAVLELTDDPELIDQAHEAMTQADQYIADSAAYAVSGDLGSDIALDDPDDLGTADTQLEDEELMAETAISHADLDQFANHSETQLDSELNLDELEELSVSDLDSSPLNAEIDLDKVDLSKSQRQAGGIEAEEDLNEISIDLSDDDLDLEDLSGDLQFEDLMNSGNLDSESQQEMLNPNPITAENPFAQGGEGLSDQIDEDAIATQLESDPFELDDEQISDSVEQEIRQPDEDWSGNFLAEEPPENYDTLPQSETSETDSEEAFEIDDIMELGDFEDDEPASKVATQKNGRPQTQPTVAHSTTRDVSENAEGIDLDEFNSDLDDIFQSLQFAPDSENEDISDDLHFSSSDQPATTSATLTEQANGKSYIPPTEEIEEFDEELHGGDAEAETLLMEPSGSTEDDFQLDAQSGFTLDDFSADPDLESDDDEAHTPSFFNQSSWSNGSSVNNGNTEHEEMFDASEIPDSFGLDAFDDETFAEEADFSDANGTSHVPEDEFSDGFTESEDFNEDLSTTSSNLDEDDFLDNFDEFDDLGNLPDFEATREEEAAFISDSSGFNEEVDSDFTVTASSALDRDNSAISNDELFNVPLDQEVVTTFTSQIDDPAEAAVTVEQGAFAFLENKTFKVKFLYTAIGTGVATLILVATATNIITQTAALNQKREVVNSLRTSGWIVTMVAGVTSFLSAWGLGNIASRQMSKASQDLQHQFDSISRNNLKARATVYAIDELGQMSAKFNRMAQFIETTTVEAQRKAEEQEEAKENLQRQVIRLLDDVEGAARGDLTVTAEVTADVLGAVADSFNLTIQNLREIVVQVKQAARQVSKGATDSATFAKDVAGDALRQAEELAATLNSVQVLTDAIQRVADSAREAEEVARTAADVATRGGEAVQMTVAGILKIRETVAETTREVKRLAESSQEISKIVAIISTIASRTNLLALNASIEAARAGEAGRGFAIVADEVRQLADRSAKSLKDIEQIVMQIQSQTNSVMMAMEEGNQQVIDGTRLAEKAKQSLDNIIQVTNRIDVLVRSITADTVEQNETARAVAQVMQAVEHSAQETSQEAHRVSNALSNLVGVARDLLTSVERFRVDPSERK

InterPro output


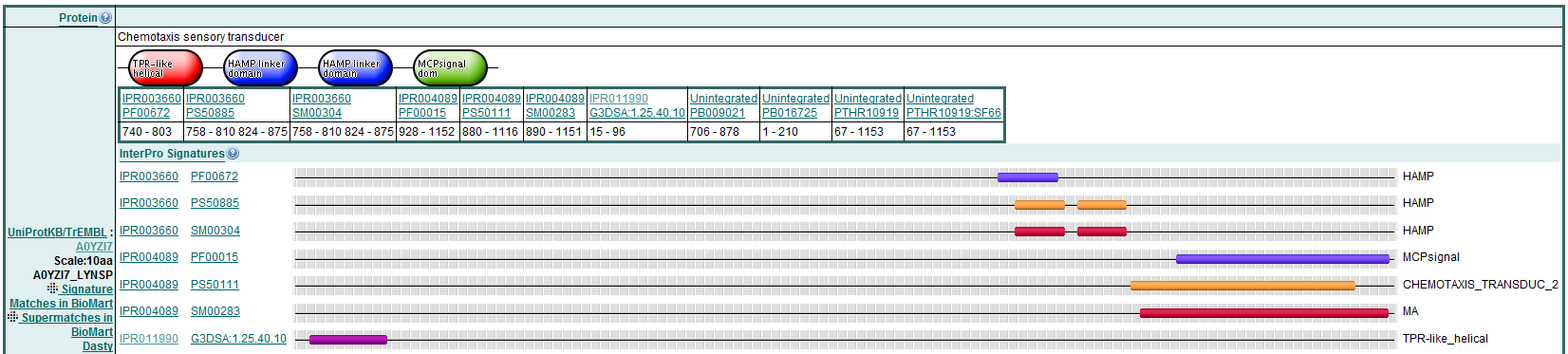


# 3D model of TPR region using template pdb 1NA3: Main et al. (2003) Design of stable alpha-helical arrays from an idealized TPR motif. [Structure](http://www.ncbi.nlm.nih.gov/pubmed/12737816?dopt=Abstract) 11:497-508.


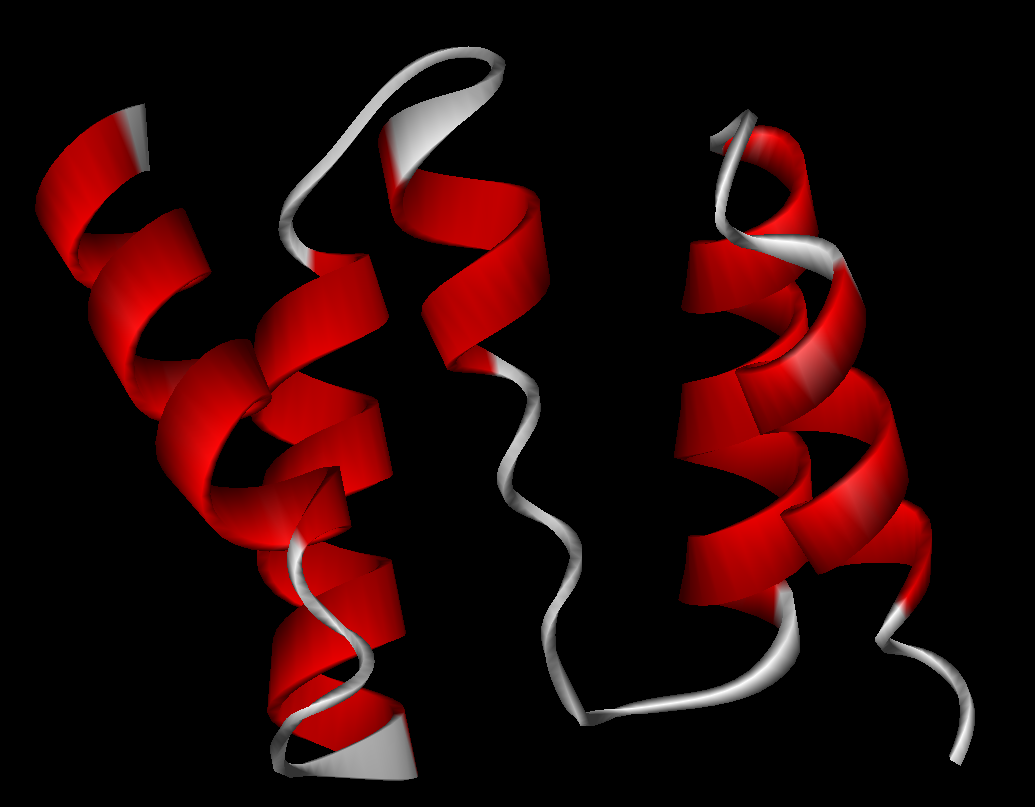


Sequence alignment of chemoreceptor – TPR fusions. Sequence fragments predicted to form a TPR are shaded in yellow.

10 20 30 40 50 60 70 80

| | | | | | | |

B5W0C4_SPIMA ------MTASAE-YLQEYQKTEAAYTDGKLDEAATLVYKLVEDYPEDPFARLLCGHIY-YGLQQYDVAREQYEVVLSLTE

D4ZR05_SPIPL ------MTASAE-YLQEYQKTEAAYTDGKLDEAATLVYKLVEDYPEDPFARLLCGHIY-YGLQQYDVAREQYEVVLGLTD

A0YZI7_LYNSP ------MQASTD-FLQEYQQTEAAYNQGNYEEAAALVYQLVENYPEDPSARLLCGYIYGYGLQQFEVAREQYFAVLELTD

D8FZE5_9CYAN ------MATTTD-FEQEYQRAEAAYLEGNYEEAAALVYQLVEDFPEDPSARLLCGHIY-CYGLQYDVAREQYMVVLSLTD

B2ITW6_NOSP7 MKLEEEMAASIDNYEPTYQQAMTAYVQRNYEVAATLVDQVVQNLPNDPNTHLLRGHIY-YVLQQYDVAKEEYQQVLSLTN

B7JXP7_CYAP8 ------MAPGTD-YSKLYNQAYVAYGQGEYEEAATLIEPMAEAYSQDPHVLLLRGHIH-LSLQQYETACADYQSVLTLTD

C7QXD9_CYAP0 ------MAPGTD-YSKLYNQAYVAYGQGEYEEAATLIEPMAEAYSQDPHVLLLRGHIH-LSLQQYETACADYQSVLTLTD

A3IRC0_9CHRO ------MASGTD-YAKLYNEAYIAYGQGNYEEAATLIEPMAGAFPEDPDVLLLQGHIY-FSLGRYETACQQYQSVINRAD

E0UEC6_CYAP2 ------MTSGID-YVKQYGKANAAYCRGDLDEAVSLIDQLAQEYPDDPHTLLLRGHIF-LSMQQYQIAQQEYEKVLKITQ

90 100 110 120 130 140 150 160

| | | | | | | |

B5W0C4_SPIMA DPALVEQAEDYLTEASQFCEDSETGSSLGDISLDDEFD-DEFDDAQETILEDLSSVGDDANGSLDFDLEEELD-LNAIDE

D4ZR05_SPIPL DPELVQQAEDYLTEASQFCEDSETGSSLGDISLD-----DEFDDAQETILEDLSSVGDDANGSLDLDLEEELD-LNAIDE

A0YZI7_LYNSP DPELIDQAHEAMTQADQYIADSAAYAVSGDLGSDIALDDPDDLGTADTQLEDEELMAETAISHADLDQFANHS-ETQLDS

D8FZE5_9CYAN DTGFVDHANNGIAYAEQFAVG-APGESLENFSED-----------------------------LDLEMYPGLSGENDLEQ

B2ITW6_NOSP7 EQEIIGFANNGIENINQYLQS--FGGQIDTSGSQ------------------------------EQIHSSEMPDPLAYSE

B7JXP7_CYAP8 QQDLLKLAQQGLAQAQEMLGQ----ASPNNLNHS----------------------------------QKSAS-------

C7QXD9_CYAP0 QQDLLKLAQQGLAQAQEMLGQ----ASPNNLNHS----------------------------------QKSAS-------

A3IRC0_9CHRO RPDLVECANQGLQQARQFLHQ----SGNNHKTDD----------------------------------NLQQP-------

E0UEC6_CYAP2 QPDLLDYAERGLEQIQLVESD----FDEMDFTID----------------------------------ETEGF-------

170 180 190 200 210 220 230 240

| | | | | | | |

B5W0C4_SPIMA QL--DELEELTAASQQLGKYPQKPSATIETLD------DALDLYDDEDMGMEEIDISSALDDLDELS-NLEFEEGTEENL

D4ZR05_SPIPL QL--DELEELTAASQQLGKYPQKPSATIETLD------DALDLYDDEDMGMEEIDISSALDDLDELS-NLEFEEGTEENL

A0YZI7_LYNSP ELNLDELEELSVS--DLDSSPLNAEIDLDKVDLSKSQRQAGGIEAEEDLNEISIDLSDDDLDLEDLSGDLQFED--LMNS

D8FZE5_9CYAN P-------------------------DLSLFG-------GG-SDLESDLGTSSLELDNAGMMGMGLS-ELDFEN----NL

B2ITW6_NOSP7 P-------------------------ELEDLD------------PSEEFDSNNLDLN--------------FFG---EHQ

B7JXP7_CYAP8 -----------------------------------------------YLGSSSLNDEDED------------EE---DGL

C7QXD9_CYAP0 -----------------------------------------------YLGSSSLNDEDED------------EE---DGL

A3IRC0_9CHRO -----------------------------------------------MINSESMKIEQQ---------------------

E0UEC6_CYAP2 -----------------------------------------------TVSQANLNTDESF------------EQ---PDF

250 260 270 280 290 300 310 320

| | | | | | | |

B5W0C4_SPIMA EAIDDDLGDLDRSADELEPENLNGKASVGGEELNDSLEDEFDLEEDNFNPLELEANPLVDDSNFDLDDEVDPDLTNPLN-

D4ZR05_SPIPL EAIDDVLGDFDISADELEPENLNGKASVGGEELNDSLEDEFALEEDNFNPLELEANPLVDDSNFDLDDEVDPDLTNPLN-

A0YZI7_LYNSP GNLDSESQQEMLNPNPITAENP---FAQGGEGLSDQIDEDAIATQLESDPFELDDEQISDSVEQEIRQPDEDWSGNFLAE

D8FZE5_9CYAN ENYANDAELLNPFGESPNFDSN----SSGSGGMAAFADADLDSG------VELGG--------LDFDDDAMAMADTQLT-

B2ITW6_NOSP7 ETVDEVKELSSNSPFDIPTEDS--------IGMGKIPDSSITFGD---DPFALDEE----SEEQDFNSSSYEEKTELDLP

B7JXP7_CYAP8 IWQDNEEGEADWDSN-FQIDEN----------------------------LDWD---------FDEEDFGEPTIGHD---

C7QXD9_CYAP0 IWQDNEEGEADWDSN-FQIDEN----------------------------LDWD---------FDEEDFGEPTIGHD---

A3IRC0_9CHRO -WSDSGEVSGDWQGEEFAVDG-----------------------------LDWDSG------IFDDDDLGEPTIGHQGN-

E0UEC6_CYAP2 SHWSQHPDMPDLEEMDWGNQPT----------------------------VHWN---------PDDDDMGEPTIGKEFS-

330 340 350 360 370 380 390 400

| | | | | | | |

B5W0C4_SPIMA ------NPFAQGQETDEEEEDPDLNFDLETSDPFVMDEEDELVLTEGL--DPEDPMSGSLEQLSEDLFE-EDLIDDSHKD

D4ZR05_SPIPL ------NPFAQGQETDEEEEDPDLNFDLETSDPFVMDEEDELVLTEGL--DPEDPMSGSLEQLPEELFE-EDLIDAPHKD

A0YZI7_LYNSP EPPENYDTLPQSETSETDSEEAFEIDDIMELGDFEDDEPASKVATQKN--GRPQTQPTVAHSTTRDVSENAEGIDLDEFN

D8FZE5_9CYAN ------FPAPKEKPSPPAQEQ-------RQADVFAGAASKQNGRTIG---SQSLADSGLADSGIEDLGEFPEDFDDMFAP

B2ITW6_NOSP7 -------AFWQEDMSEEIHEES------LVNSHFSENEINHDRGNSAI--DNNNSSSSNSQ-TSSSKNNFSDLLIEPKSP

B7JXP7_CYAP8 --------PSQANPFDNAGQTTFR----PPQADVASLAVEDDLN---------------LETPDT---------WQSPFT

C7QXD9_CYAP0 --------PSQANPFDNAGQTTFR----PPQADVASLAVEDDLN---------------LETPDT---------WQSPFT

A3IRC0_9CHRO --------ASQSSEEQPEKQNSFE----MPVDPFTTLSEVRPFDSQ-------------TDIPNTNASE-E-EGWTDFVD

E0UEC6_CYAP2 -------RHQFANPFAEDAETSFN----PDLSQFEDLAETFGLPSEPSHHPELERYVNSLDQPGADDDQ-TNFYWHNNAD

410 420 430 440 450 460 470 480

| | | | | | | |

B5W0C4_SPIMA SPGTNTPGVTQEHQDS-LDDLDDDFSLTDLPLTDGPITNEPIVDNVAEEIKP-PDLGDVQDELDIGELELE---------

D4ZR05_SPIPL SPATNTPGVTQEHQDS-WDDLGDDFSLTDLPLTDEPITNETIVDHVALEIKP-PDLGDVQDELDIGELELE---------

A0YZI7_LYNSP SDLDDIFQSLQFAPDSENEDISDDLHFSSSDQPATTSATLTEQANGKSYIPPTEEIEEFDEELHGGDAEAETLLMEPSGS

D8FZE5_9CYAN LELPDDLNFEESYPPA----ET--NGKQKMPLQPQPTAEPGQTRSRTSGIQA-------KEGSDLGGSKSN---------

B2ITW6_NOSP7 EPKIGNLEYNKSNFGG---------ETLLIVVEEEPINSSATTNNNSRYDLP------ETEAHDWLKSK-----------

B7JXP7_CYAP8 EAME-DFPFTDDE---------------DLRTEPHPTASVGKNTFLVTPKLD-------NSRENPEGTPI----------

C7QXD9_CYAP0 EAME-DFPFTDDE---------------DLRTEPHPTASVGKNTFLVTPKLD-------NSRENPEGTPI----------

A3IRC0_9CHRO EDFP-DFSFGDEQ---------------GLETEAHTGAGDEENTFVVSSSLN-------PPSLSNRGS------------

E0UEC6_CYAP2 EDEQRHLSFIEEDNNG--------FNSNGYRSNFDQFSDTGESTLVVSSDLP-------SNALKSNATKFL---------

490 500 510 520 530 540 550 560

| | | | | | | |

B5W0C4_SPIMA -EDDIFSPLED---MADARHQTVAETNGN-GNGKGLVKPDQISLNKTPNNNL-DEEEENFSDFNLDG-DKINDSVSEELE

D4ZR05_SPIPL -EDDIFSPLED---MADARHQTVAETNGN-GNGKGLVKPDQISLNKTPNNNL-DEEEENFSDLNLDEFDQVDDSVSDEFE

A0YZI7_LYNSP TEDDFQLDAQSGFTLDDFSADPDLESDDDEAHTPSFFNQSSWSNGSSVNNGN-TEHEEMFDASEIPDSFGLDAFDDETFA

D8FZE5_9CYAN ----YSVAEDET-LLMGSHSAPEALSEIG---KP-SKDLNSGFKSTTQGSKA-AKGEDLQNGFNIPDSFDLDSFDDDAFA

B2ITW6_NOSP7 -----DLEVEQ-----------EFQTESS-------------DNYSSFKSDI-PLEEKQMKSSEIIS---KNSFDDENFD

B7JXP7_CYAP8 -------------------ESFLRPSSRNLLNYNSDEDLTLPPSSSSMGN----QENQLVENLDS------------DLS

C7QXD9_CYAP0 -------------------ESFLRPSSRNLLNYNSDEDLTLPPSSSSMGN----QENQLVENLDS------------DLS

A3IRC0_9CHRO -------------------TPPLQDSSHDWSNSPAQDDPTVHATPQMMSSDQVQEHNRVLDEFDAFQ-----EEALEDLS

E0UEC6_CYAP2 -G---KNPSNG----K-FSENLAADHSDSWNEEAFRKEPQQRKPLSSHTQQP-ANSDGFLNDLEVFN-----DDDLDGLS

570 580 590 600 610 620 630 640

| | | | | | | |

B5W0C4_SPIMA LD-DIPDTFDLDSLEESTVSNG-TANGAIAHNKTNQTSGSQVNEFLEDFEEFDDVEGFGIPDAAG---YAFMPDSADLDD

D4ZR05_SPIPL LD-DIPDTFDLDSLEESTVSNG-TANGAIAHNKTNQTSGSQVNEFLEDFEEFDDVEGFGIPDAAG---YAFMPDSADLDD

A0YZI7_LYNSP EEADFSDANGTSHVPEDEFSDGFTESEDFNEDLSTTSSNLDEDDFLDNFDEFDDLGNLPDFEATREEEAAFISDSSGFN-

D8FZE5_9CYAN DS---------FSSYEETPSHSFGMAGPDSDFPLTPSPQTDRGDWIDDFDEFGDVGNLPDFDISDDANSSILTTGGSSGF

B2ITW6_NOSP7 ME----------AFESAFGSDGLSSYEDSSNILNGENSKSN-IDFLDDFEEFDDLGNIPGFDLIEE--DSNFGDAAMYS-

B7JXP7_CYAP8 ----------HLTEMAAELPDSDIFTRSAEVFSD--NLSLDASDLMSAAPTPTTVTWVKPNEAMG----GDLSVASNQGI

C7QXD9_CYAP0 ----------HLTEMAAELPDSDIFTRSAEVFSD--NLSLDASDLMSAAPTPTTVTWVKPNEAMG----GDLSVASNQGI

A3IRC0_9CHRO NF--------DFTEMSDQLPDSDLFTQASEDGSG--SIGLQTGDLLSE-PGPQTVNWVKSDDMAN----TSLSDSEVKIV

E0UEC6_CYAP2 QF--------DITDVAQSLPDSGLFERHTDGLDSGLETGSGISAAAMEASSISKINWTDVGENSS----ANLTGASTRFI

650 660 670 680 690 700 710 720

| | | | | | | |

B5W0C4_SPIMA DDLDSNFLGRSSSIPDN----DSSAIYEDDVFNTPTEREAITAFSNLSEDSVDTNISVEQGSFAFLENKPLRSKSFYIAL

D4ZR05_SPIPL DDLDSNFLGRSSSIPDN----DSSAIYEDDVFNTPTEREAITAFSNLSEDSVDTNISVEQGSFAFLENKPLRSKSFYIAL

A0YZI7_LYNSP EEVDSDFTVTASSALDR----DNSAISNDELFNVPLDQEVVTTFTSQIDDPAEAAVTVEQGAFAFLENKTFKVKFLYTAI

D8FZE5_9CYAN GGMTGGVMGATTSNLDFGDNSDGSAIRDDEIFSISGTPESVPSFT-PTEEPIDATVTVEQGGLAFLENAPLITKQLYTAI

B2ITW6_NOSP7 APAEASGSGRSQSTDTSG---SNAADREEELFSMTGSHEGVPVFSQTDVSKLEPNVSVEQGWLAPLENASIERKQWLIAG

B7JXP7_CYAP8 KPTVEVAQQGWAAWFIN-------ASLKHKPWIAAGATGIASFLMVLLVSTFTSVMSPKPQSGTTPVP-QTSQSTPKPQN

C7QXD9_CYAP0 KPTVEVAQQGWAAWFIN-------ASLKHKPWIAAGATGIASFLMVLLVSTFTSVMSPKPQSGTTPVP-QTSQSTPKPQN

A3IRC0_9CHRO KPTAE-RQQGYLRWFFN-------APLKIKPWMAAGSTGLVSFMAILLVSSFAWLVSPKPEATEVGEEEATTEQVEQSDT

E0UEC6_CYAP2 KPTVE-IEQGKFSALKN-------LPLKRKQWITAGIAGLTSVATIFLISSAIWMFSPKQPSKKADSPANKTESVSSNPK

730 740 750 760 770 780 790 800

| | | | | | | |

B5W0C4_SPIMA GSGLVTLIAVAVATNIATKVAASSYQGEVVNYLRRSGWLMTIVAGASSFGTAFAMGRITSQQLEKATGDLQKQFDAIARG

D4ZR05_SPIPL GSGLVTLIAVAVATNIATKVAASSYQGEVVNYLRRSGWLMTIVAGASSFGTAFAMGRITSQQLEKATGDLQKQFDAIARG

A0YZI7_LYNSP GTGVATLILVATATNIITQTAALNQKREVVNSLRTSGWIVTMVAGVTSFLSAWGLGNIASRQMSKASQDLQHQFDSISRN

D8FZE5_9CYAN GTGLISLVAVALVTNFASYQALKQDKPEAIAYLRQTGWAMTAAAGFTSFLTAWGLGHLVARQVSKSTDDLQVQFDSVSQG

B2ITW6_NOSP7 SVGIVSALVVATVSFVATTFSPVQQR----ESVRNTGWAMSLAAGIAGFATAGFMGNLTLKQIRRTTNDLQAQFEAVRQG

B7JXP7_CYAP8 NQKPAAKATNKPSAAKSGS----------------PVLLMALLGGLTGFSTTLLFSFLICGQVKRTIEDLQAQFDAMYAG

C7QXD9_CYAP0 NQKPAAKATNKPSAAKSGS----------------PVLLMALLGGLTGFSTTLLFSFLICGQVKRTIEDLQAQFDAMYAG

A3IRC0_9CHRO TETSSEETEENEKPAKSGN----------------PILLLALTGGLVGFGGTLFFGLISSYQLKRTIDDLQTQFDSMYAG

E0UEC6_CYAP2 KSDPKTDKKTTPKKNSSSSTALQAQQTAAISPFSPSMLLMMLLTGGATFGATLFFARLSTNQIKQTVDDLQTQFDAIYAG

810 820 830 840 850 860 870 880

| | | | | | | |

B5W0C4_SPIMA NLNARVNVYAEDELGQMCAKFNYMAQFIESTTREAQRKAEEQEEAKENLQRQVIRLLDDVEGAARGDLTVSAEVTADVLG

D4ZR05_SPIPL NLNARVNVYAEDELGQMCAKFNYMAQFIESTTREAQRKAEEQEEAKENLQRQVIRLLDDVEGAARGDLTVSAEVTADVLG

A0YZI7_LYNSP NLKARATVYAIDELGQMSAKFNRMAQFIETTTVEAQRKAEEQEEAKENLQRQVIRLLDDVEGAARGDLTVTAEVTADVLG

D8FZE5_9CYAN NLEARATVYSEDEFGKMSAKFNHMAKVILTTTSDARRKADEQEQAKEDLQRQVIRLLDDVEGAARGDLTVQAEVTADVLG

B2ITW6_NOSP7 NLNAQATVFSEDELGHLSTGFNEMARVIFTTTSEAQRKADEQEEAKENLQRQVIRLLDDVEGAARGDLTVQAEVTADVLG

B7JXP7_CYAP8 DFNVKATIHSSDELGQLSLSFNQLARIILTTTSEAQQRAAETEQAQEDLTRQVIRLLDDVEGAARGDLTVEAVVTADVLG

C7QXD9_CYAP0 DFNVKATIHSSDELGQLSLSFNQLARIILTTTSEAQQRAAETEQAQEDLTRQVIRLLDDVEGAARGDLTVEAVVTADVLG

A3IRC0_9CHRO DYNVKATIYSEDELGQLSHSFNQLSRVILTTTSEAQKRAAEMEQAREELQRQVIRLLDDVEGAARGDLTVEAEVTADVLG

E0UEC6_CYAP2 DFNVKATVYSEDELGQLSARFNQMAQAILTTTSEAQRRAAETEQQKEDLQRQVIRLLDDVEGAARGDLTVEAEVTADVLG

890 900 910 920 930 940 950 960

| | | | | | | |

B5W0C4_SPIMA AVADSFNLTIQNLREIVVQVKQAARQVSRGATDSASFAKDVAGDALRQAEELAATLNSVQLLTDAIQRVADSAKEAEEVA

D4ZR05_SPIPL AVADSFNLTIQNLREIVVQVKQAARQVSRGATDSASFAKDVAGDALRQAEELAATLNSVQLLTDAIQRVADSAKEAEEVA

A0YZI7_LYNSP AVADSFNLTIQNLREIVVQVKQAARQVSKGATDSATFAKDVAGDALRQAEELAATLNSVQVLTDAIQRVADSAREAEEVA

D8FZE5_9CYAN AVADSFNLTIQNLREIVHQVKQAARDVSTGASESATFAQGLSSDALRQAEELAATLNSVQVLTDAIQRVAESAREAEEVA

B2ITW6_NOSP7 AVADAFNLTIQNLRDIVQQVKVAAKDVTKGATNSETFARALSSDALRQAEELAVTLNSVQVMTDSIQRVAEAAREAETVA

B7JXP7_CYAP8 AVADAFNLTIQNLREIVGQVKQAAKQVNKGSTDSESFARNNSSDALRMAEELAVTLNSVQVMTESIQRVAENAREAEEVA

C7QXD9_CYAP0 AVADAFNLTIQNLREIVGQVKQAAKQVNKGSTDSESFARNNSSDALRMAEELAVTLNSVQVMTESIQRVAENAREAEEVA

A3IRC0_9CHRO AVADAFNLTIHNLREIVAQVKRTAKQVNKSSTDSELFARNNSRDALRMAEELAVTLNSVQVMTESIQRVAENAREAEEVA

E0UEC6_CYAP2 AVADAFNLTIQNLREIVRQVKKAAEQVNKGSTDSELFARNQSSDALRMAEELAVTLNSVQMMTDSIQRVAENAREAEEVA

970 980 990 1000 1010 1020 1030 1040

| | | | | | | |

B5W0C4_SPIMA RTAAAVATKGGEAVEMTVAGILKIRETVAETTRDVKRLAESSQEISKIVAIISNIASRTNLLALNASIEAARAGEAGRGF

D4ZR05_SPIPL RTAAAVATKGGEAVEMTVAGILKIRETVAETTRDVKRLAESSQEISKIVAIISNIASRTNLLALNASIEAARAGEAGRGF

A0YZI7_LYNSP RTAADVATRGGEAVQMTVAGILKIRETVAETTREVKRLAESSQEISKIVAIISTIASRTNLLALNASIEAARAGEAGRGF

D8FZE5_9CYAN RGAAASAIKGGEAVERTVAGILEIRETVAETTRKVKRLAEASQEISKIVALIATIASRTNLLALNASIEAARAGEAGRGF

B2ITW6_NOSP7 RDASTIALKGGEAVENTVAGILEIRETVAETTRKVKRLAESSQEISKIVALISQIASRTNLLALNASIEAARAGEAGRGF

B7JXP7_CYAP8 SVSSVTALKGGEAVERTVAGILGIRETVSDTTRKVKRLAEASQEISKIVALISQIASRTNLLALNASIQAARAGEAGRGF

C7QXD9_CYAP0 SVSSVTALKGGEAVERTVAGILGIRETVSDTTRKVKRLAEASQEISKIVALISQIASRTNLLALNASIQAARAGEAGRGF

A3IRC0_9CHRO HTSSVTALKGGEAVERTVAGILQIRETVSETTRKVKRLAEASQEISKIVALISQIASRTNLLALNASIQAARAGEAGRGF

E0UEC6_CYAP2 RTSSVTALKGGESVERTVAGILQIRETVSETARKVKRLAEASQEINKIVAVVSQIASRTNLLALNASIQAARAGEAGRGF

1050 1060 1070 1080 1090 1100 1110 1120

| | | | | | | |

B5W0C4_SPIMA AIVADEVRQLADKSAKSLKEIEQIVMQIQSQTSSVMMAMEEGNQQVIEGTRLAEQAKRSLDDIIQVTNRIDVLVRSITAD

D4ZR05_SPIPL AIVADEVRQLADKSAKSLKEIEQIVMQIQSQTSSVMMAMEEGNQQVIEGTRLAEQAKRSLDDIIQVTNRIDVLVRSITAD

A0YZI7_LYNSP AIVADEVRQLADRSAKSLKDIEQIVMQIQSQTNSVMMAMEEGNQQVIDGTRLAEKAKQSLDNIIQVTNRIDVLVRSITAD

D8FZE5_9CYAN AIVADEVRQLADRSAKSLKEIEQIVMQIQSETGAVMTAMEEGTQQVIEGTRLAEQAKRSLEDIIQVTNRIDVLVRSITAD

B2ITW6_NOSP7 AIVADEVRQLADKSAKSLKEIEQIVMQIQSETGSVMTAMEEGTQQVIKGTKLAEEAKRSLENIIQVANRIDILVRSITSD

B7JXP7_CYAP8 AIVADEVRQLADRSAKSLKEIEQIVLQIQSETGSVMTAMEEGIQQVINLVDTSEQAKRSLEDIIDVSNRINGLVRSITAD

C7QXD9_CYAP0 AIVADEVRQLADRSAKSLKEIEQIVLQIQSETGSVMTAMEEGIQQVINLVDTSEQAKRSLEDIIDVSNRINGLVRSITAD

A3IRC0_9CHRO AIVADEVRQLADRSAKSLKEIEQIVLQIQSETGSVMTAMEEGIQQVIDVTDKSEQAKRSLEDIIDVSNHINTLVRSITGD

E0UEC6_CYAP2 AIVADEVRQLADRSAKSLKEIEQIVLQIQSETGSVMTAMEEGIQQVIDVTERSEQAKRSLEDIIQVSNRIDSLVRSITGD

1130 1140 1150 1160 1170 1180

| | | | | |

B5W0C4_SPIMA TVEQNETARAVAEVMQAVELSAQDTSQEAQRVASALSNLVGVARDLLTSVERFRVDPSEH--

D4ZR05_SPIPL TVEQNETARAVAEVMQAVELSAQDTSQEAQRVASALSNLVGVARDLLTSVERFRVDPSER--

A0YZI7_LYNSP TVEQNETARAVAQVMQAVEHSAQETSQEAHRVSNALSNLVGVARDLLTSVERFRVDPSERK-

D8FZE5_9CYAN TIEQNQTASAVSHVMQAVELTAQETSQESQRVYGSLQNLVGVARDLLTSVERFRVETAERQQ

B2ITW6_NOSP7 TVEQTETSRAVAHVMQSVELTAQETSQEAQRVSGALQHLVGVSRDLIASVERFRVETMETR-

B7JXP7_CYAP8 TVKQRENSRAVTQVMQSVELTAQEASQESQRVAGSLQTLVAISRDLLASVERFRVDKSET--

C7QXD9_CYAP0 TVKQRENSRAVTQVMQSVELTAQEASQESQRVAGSLQTLVAISRDLLASVERFRVDKSET--

A3IRC0_9CHRO TVQQQDNSKAVSQVMQSVELTAQETSQESQRVAGALQNLVGISRDLLTSVERFHIEEAEKS-

E0UEC6_CYAP2 TVKQRENSREVAQVMQSVELTAQETSQESQRVAGSLQTLVKISRDLLESVERFKVDKNDYK-
