# Supplementary material for: Genes Encoding Cher-TPR Fusion Proteins Are Predominantly Found in Gene Clusters Encoding Chemosensory Pathways with Alternative Cellular Functions
Source: PLoS One. 2012 Sep 20;7(9):e45810. doi: 10.1371/journal.pone.0045810 (PMC3447774; doi:10.1371/journal.pone.0045810)

**Analysis S2)**

AglT (*M. xanthus*)


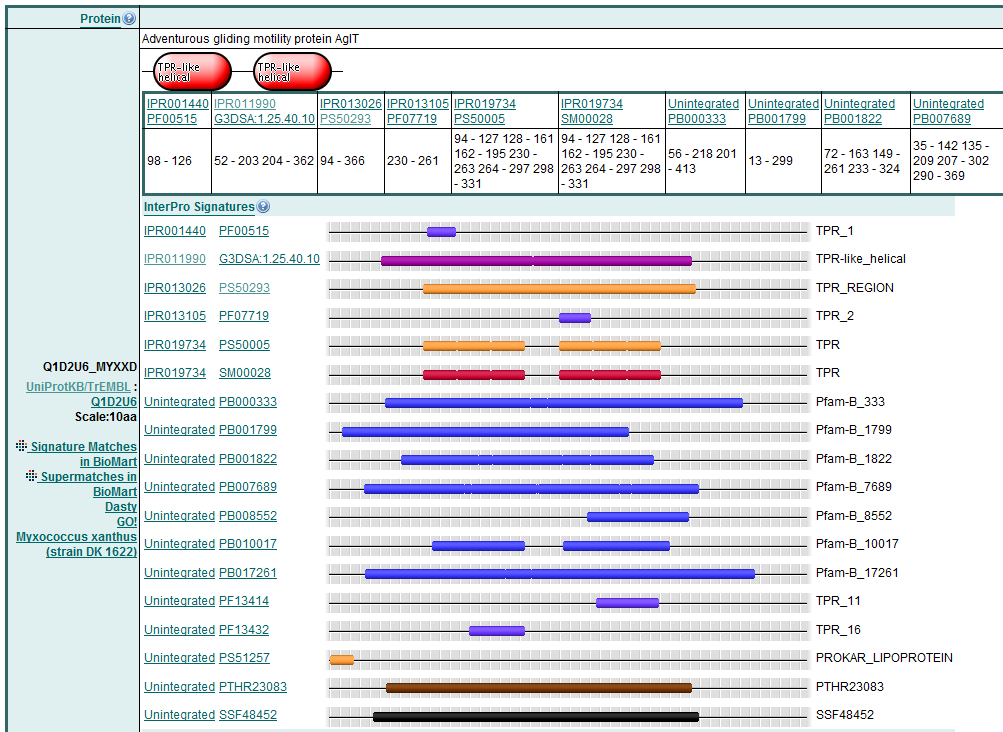


3D model of a fragment of AglT based on pdb entry 1w3b


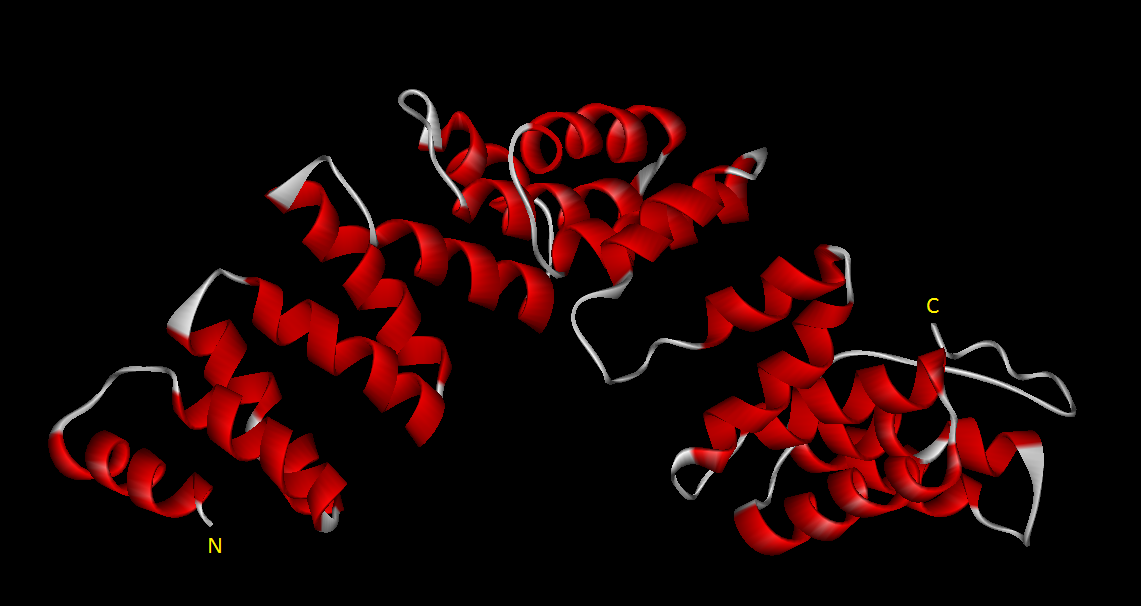


AgmK (*M. xanthus*)


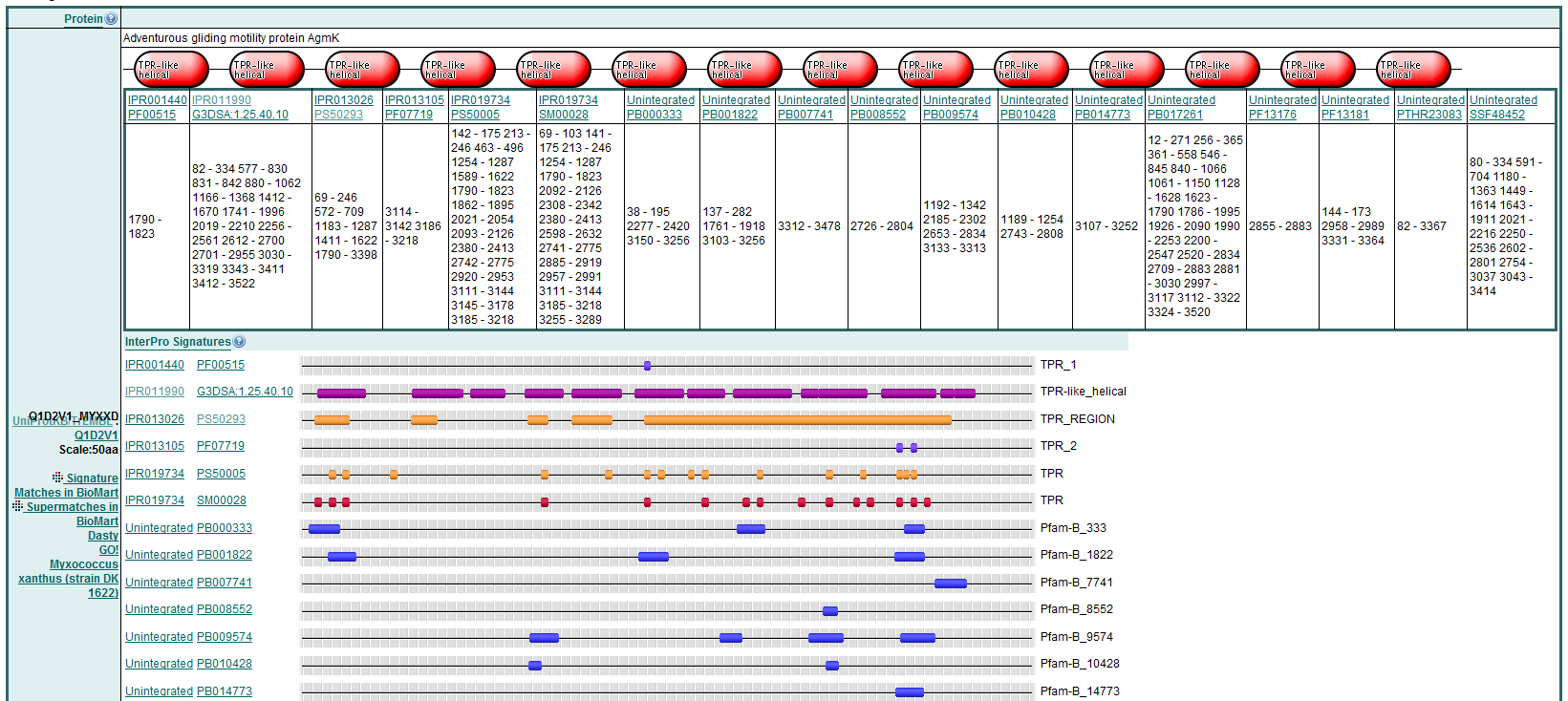


3D model of a fragment of AgmK based on pdb entry 1w3b


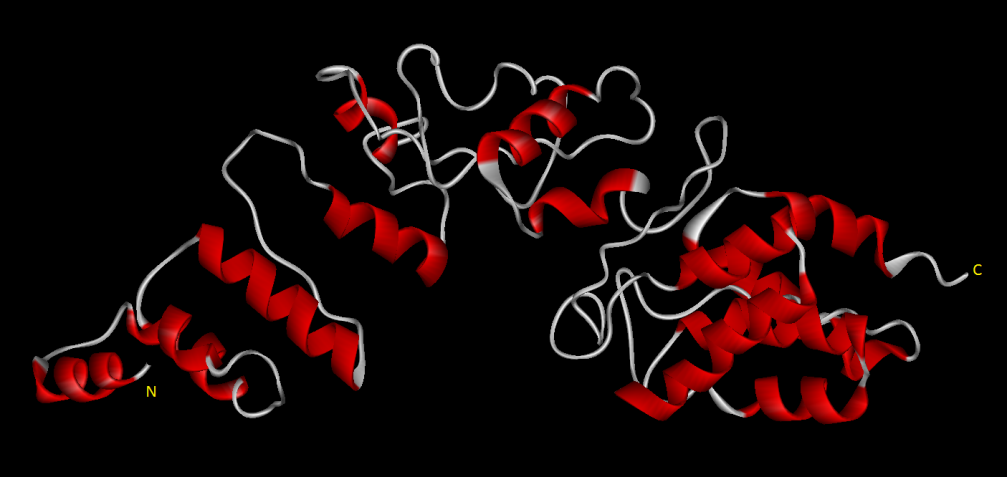


AgmU (*Myxococcus xanthus*)


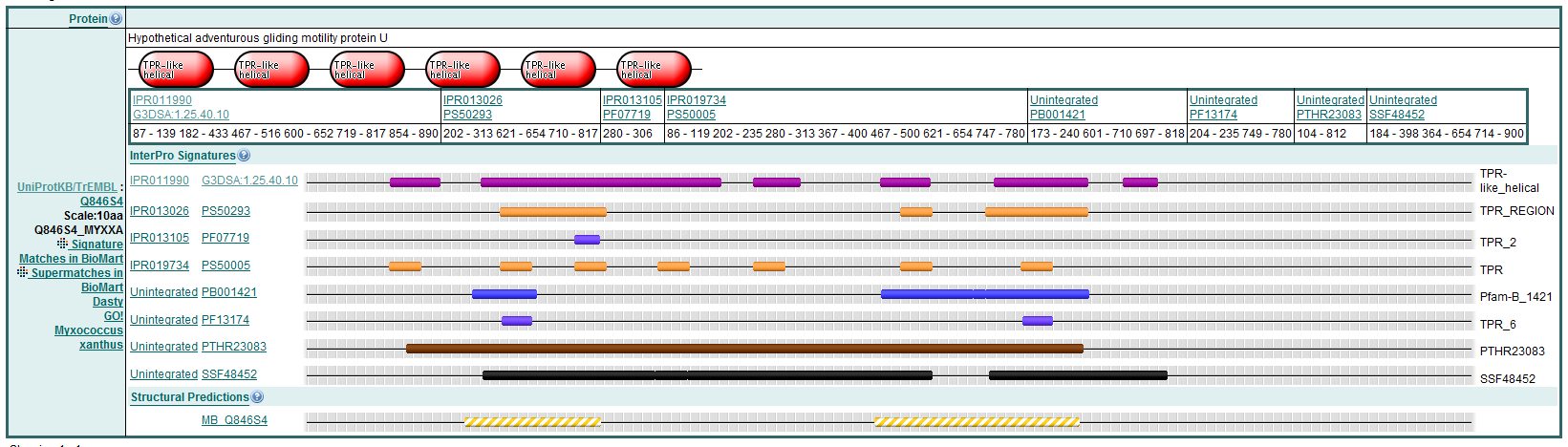


3D model


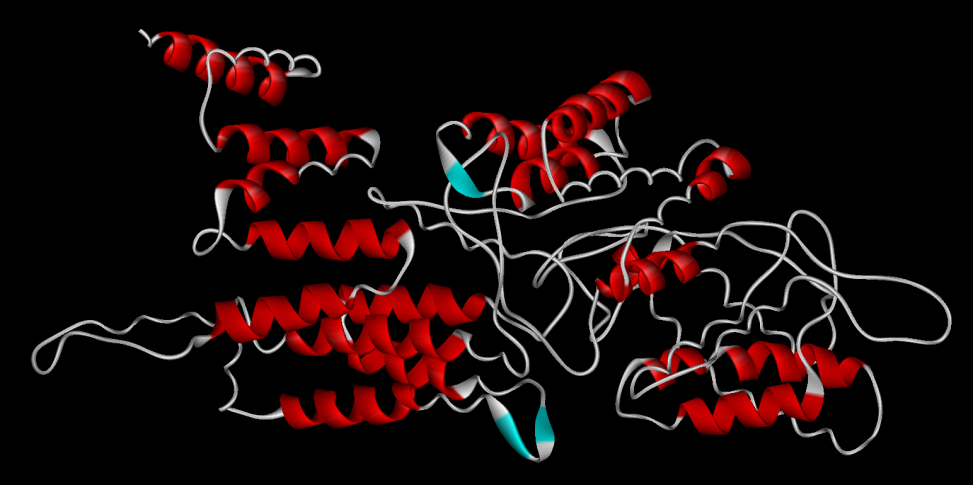


AgnA (*M. xanthus*)


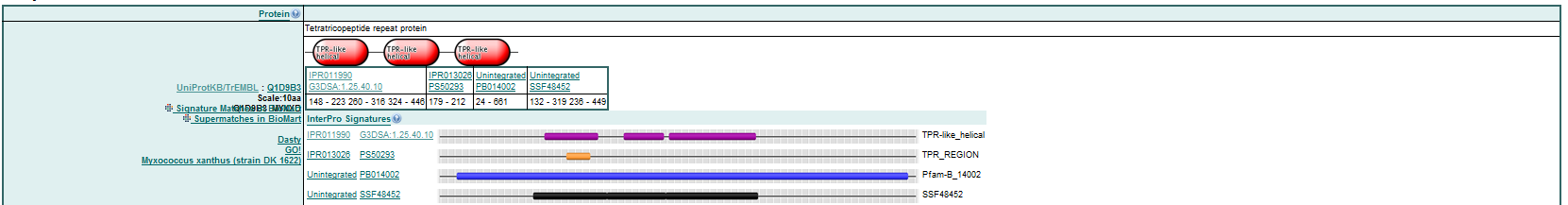


3D model


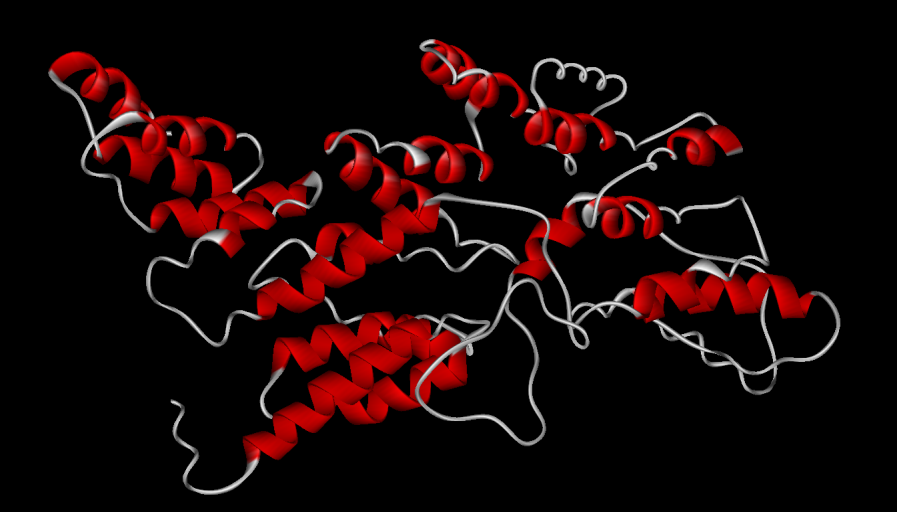


protein T (*M. xanthus*)


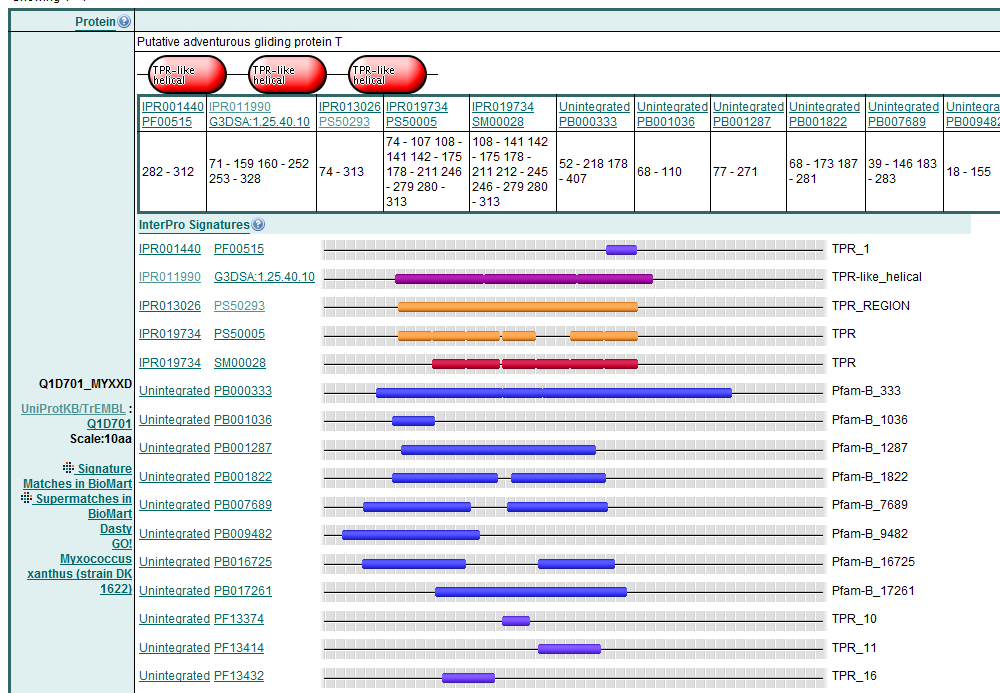


3D model of protein T based on pdb entry 1w3b


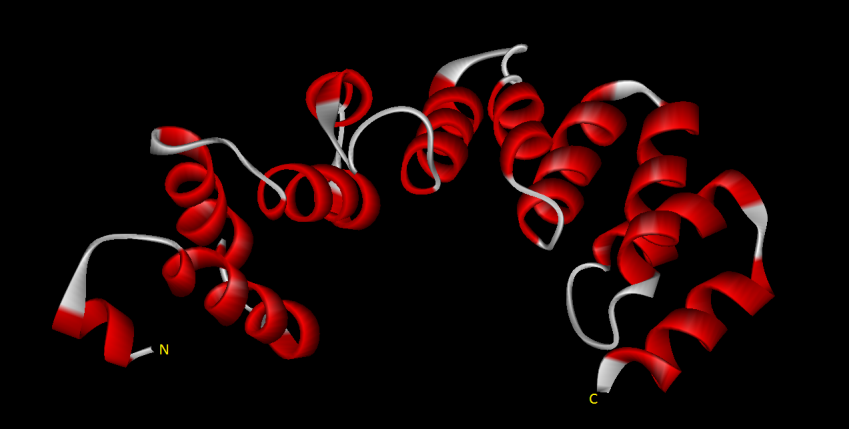


ORF Q1D897- Putative adventurous gliding motility protein (*M. xanthus*)


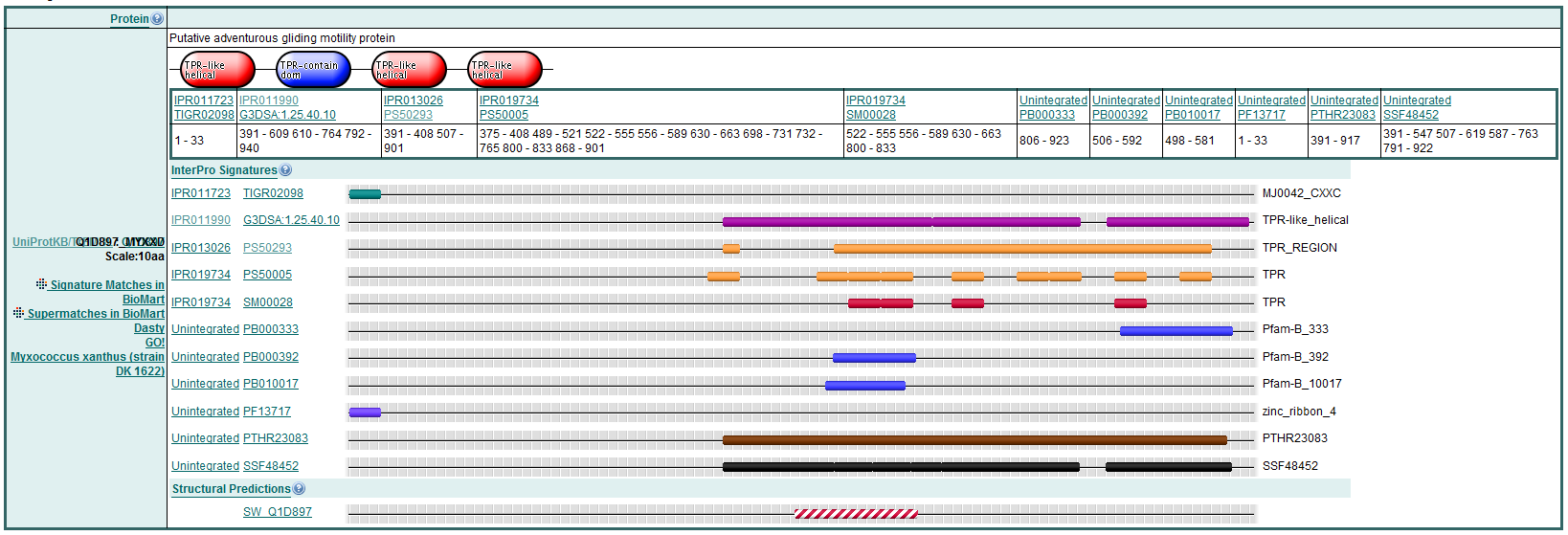


3D model of Q1D897 based on pdb entry 2GW1


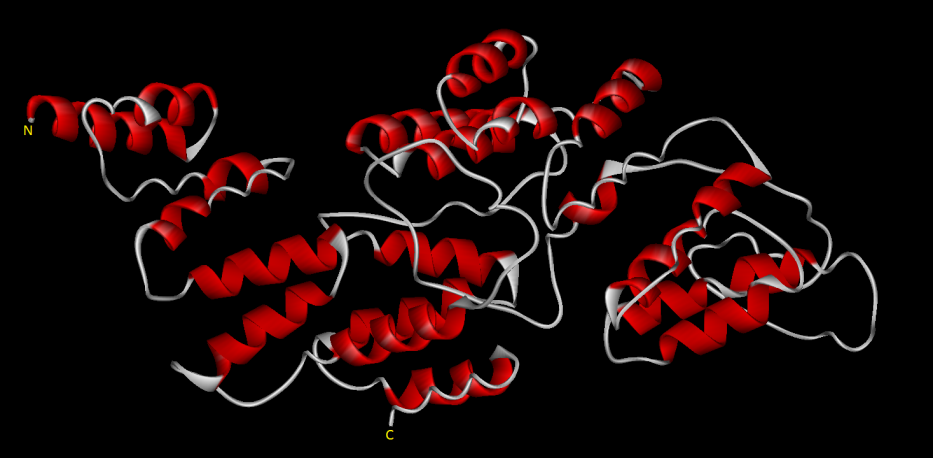


Tgl (*M. xanthus*)


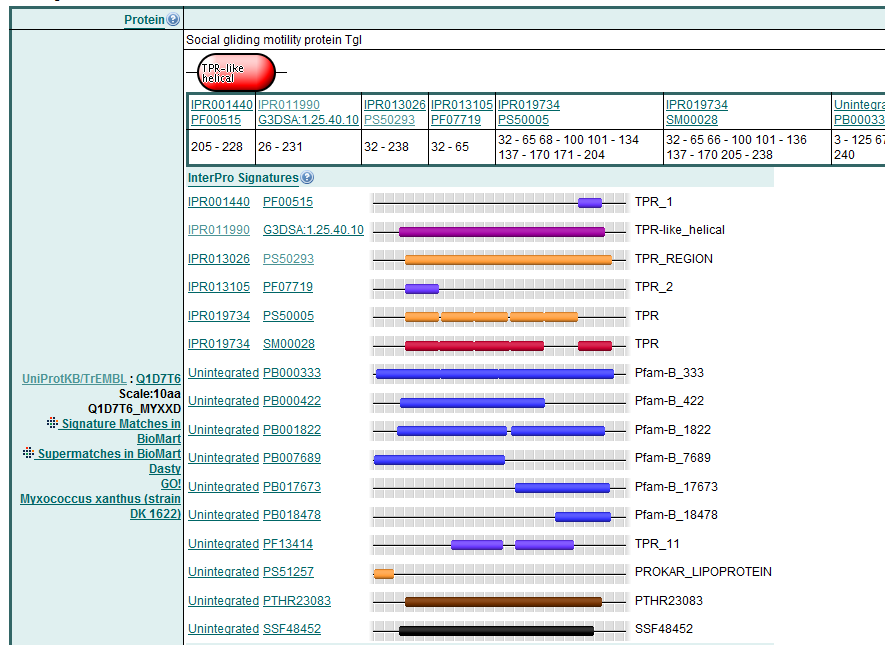


3D model of Tgl based on pdb entry 1w3b


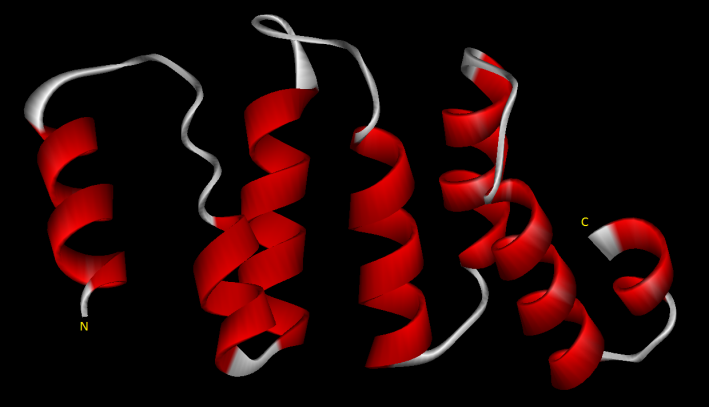


PilF (*P. aeruginosa*)


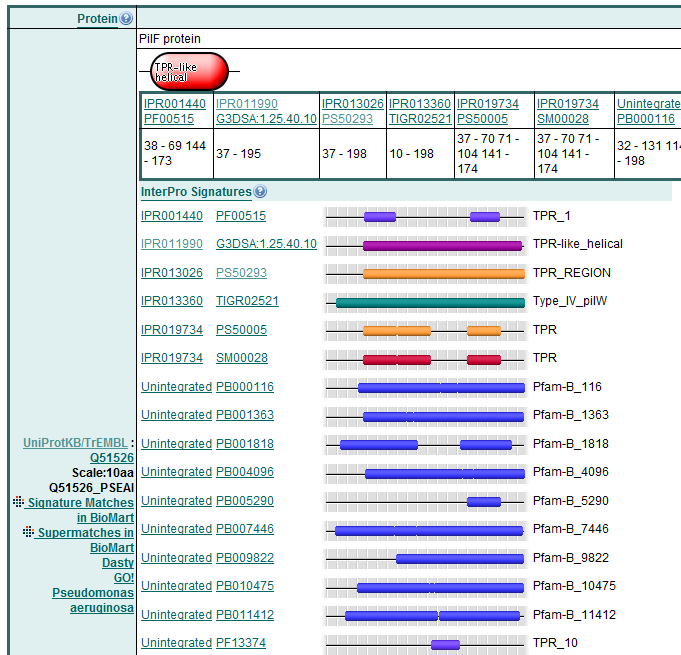


3D model of PilF based on pdb entry Pdb 3pe3


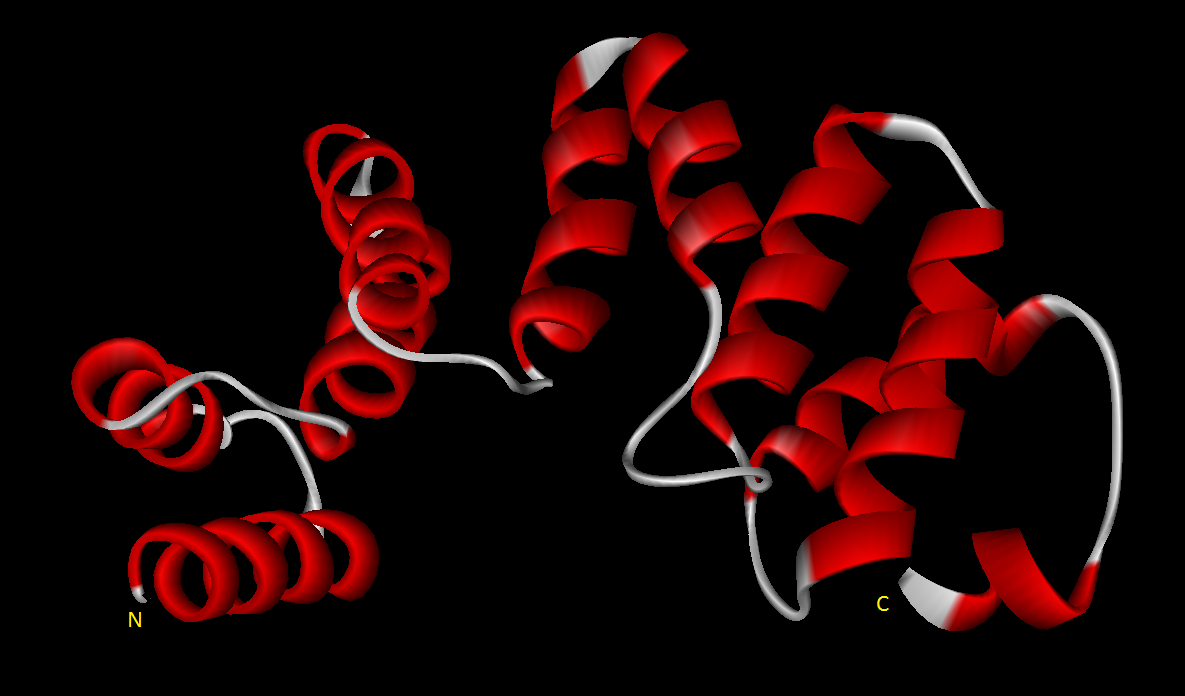

Supplement: Analysis S2 — TPR proteins shown or proposed to be involved in non-flagellum mediated motility. Shown are domain annotations in Interpro and homology models created using CPHmodels 3.2 (Nielsen et al. (2010) CPHmodels-3.0 Nucleic Acids Research 38, doi:10.1093/nar/gkq535). (DOCX) [file pone.0045810.s010.docx]
